# Traditional medicinal plant use in Northern Peru: tracking two thousand years of healing culture

**DOI:** 10.1186/1746-4269-2-47

**Published:** 2006-11-07

**Authors:** Rainer W Bussmann, Douglas Sharon

**Affiliations:** 1 University of Hawaii, Lyon Arboretum, 3860 Manoa Rd., Honolulu, HI 96822, USA; 2 San Diego Museum of Man, 1350 El Prado, San Diego, CA 94804, USA

## Abstract

This paper examines the traditional use of medicinal plants in Northern Peru, with special focus on the Departments of Piura, Lambayeque, La Libertad, Cajamarca, and San Martin.

Northern Peru represents the center of the old Central Andean "Health Axis," stretching from Ecuador to Bolivia. The roots of traditional healing practices in this region go at least as far back as the Moche period (AC 100–800).

Although about 50% of the plants in use reported in the colonial period have disappeared from the popular pharmacopoeia, the plant knowledge of the population is much more extensive than in other parts of the Andean region.

510 plant species used for medicinal purposes were collected, identified and their vernacular names, traditional uses and applications recorded. The families best represented were Asteraceae with 69 species, Fabaceae (35), Lamiaceae (25), and Solanaceae (21). Euphorbiaceae had twelve species, and Apiaceae and Poaceae 11 species.

The highest number of species was used for the treatment of "magical/ritual" ailments (207 species), followed by respiratory disorders (95), problems of the urinary tract (85), infections of female organs (66), liver ailments (61), inflammations (59), stomach problems (51) and rheumatism (45).

Most of the plants used (83%) were native to Peru. Fresh plants, often collected wild, were used in two thirds of all cases, and the most common applications included the ingestion of herb decoctions or the application of plant material as poultices.

## Background

Traditional Medicine is used globally and has a rapidly growing economic importance. In developing countries, Traditional Medicine is often the only accessible and affordable treatment available. In Africa up to 80% of the population uses Traditional Medicine as the primary healthcare system. In Latin America the WHO Regional Office for the Americas (AMRO/PAHO) reports that 71% of the population in Chile and 40% of the population in Colombia use Traditional Medicine. In many Asian countries Traditional Medicine is widely used, even though Western medicine is often readily available. In Japan, 60–70% of allopathic doctors prescribe traditional medicines for their patients, and in China, Traditional Medicine accounts for about 40% of all health care. The number of visits to providers of Traditional Medicine now exceeds by far the number of visits to all primary care physicians in the US [1-3]. Forty-eight percent of the population in Australia, 70% in Canada, 42% in the US, 38% in Belgium and 75% in France, have used Traditional Medicine at least once [4-6]. A survey of 610 Swiss doctors showed that 46% had used some form of Traditional Medicine, mainly homeopathy and acupuncture [7]. In the United Kingdom, almost 40% of all general allopathic practitioners offer some form of Traditional Medicine referral or access [8]. In the USA, a national survey reported the use of at least 1 of 16 alternative therapies increased from 34% in 1990 to 42% in 1997 [9,10].

The expenses for use of Traditional and Traditional Medicine are exponentially growing in many parts of the world. The 1997 out-of-pocket Traditional Medicine expenditure was estimated at US$ 2700 million in the USA. The world market for herbal medicines based on traditional knowledge is now estimated at US$ 60000 million [11]. The sales of herbs and herbal nutritional supplements in the US increased 101% between May 1996 and May 1998 [12].

Traditional Medicine is also gaining more respect by national governments and health providers. Peru's National Program in Complementary Medicine and the Pan American Health Organization recently compared Complementary Medicine to allopathic medicine in clinics and hospitals within the Peruvian Social Security System [13]. Treatments for osteoarthritis; back pain; neuroses; asthma; peptic acid disease; tension and migraine headache; and obesity were analyzed. The results showed that the cost of using Traditional Medicine was less than the cost of Western therapy. In addition, for each of the criteria evaluated – clinical efficacy, user satisfaction, and future risk reduction – Traditional Medicine 's efficacy was higher than that of conventional treatments, including fewer side effects, higher perception of efficacy by both the patients and the clinics, and a 53–63% higher cost efficiency of Traditional Medicine over that of conventional treatments for the selected conditions [13].

### Antecedents – medicinal plant research and traditional medicine in Peru

The primary focus of this project has been the ethnobotany of medicinal plants used on the north coast of Peru.

Fieldwork for the present study started in the markets of Trujillo (Mayorista and Hermelindas) and Chiclayo (Modelo and Moshoqueque) in 2001.

Precedents for this study have been established by the late 17^th^-century plant collections of Bishop Baltasar Jaime Martinez de Compañón [14], ethnoarchaeological analysis of the psychedelic San Pedro cactus [15], *curandera *depictions in Moche ceramics [16], and research on the medicinal plants of Southern Ecuador [17,18] used in a field guide on the medicinal plants of the region [19].

Considerable progress has been made in the overall taxonomic treatment of the flora of Peru over the last few decades [20]. However, while the Amazon rainforests have received a great deal of scientific attention, the mountain forests and remote highland areas are still relatively unexplored. The first floristic studies were conducted in the 1920's [21], followed by decades without any further research activity. Until the late 1990s little work had been done on vegetation structure, ecology, and ethnobotany in the mountain forests and coastal areas of the North.

**Figure 1:**
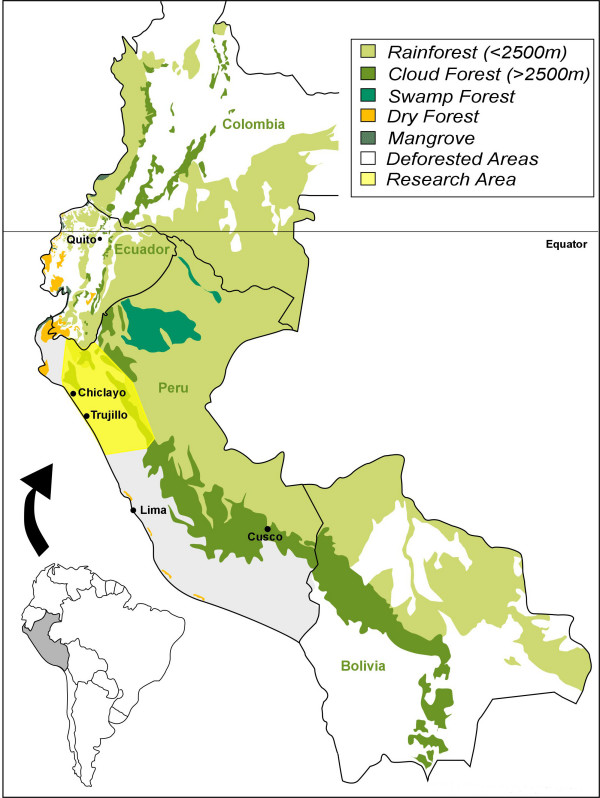
Study Area.

**Figure 2:**
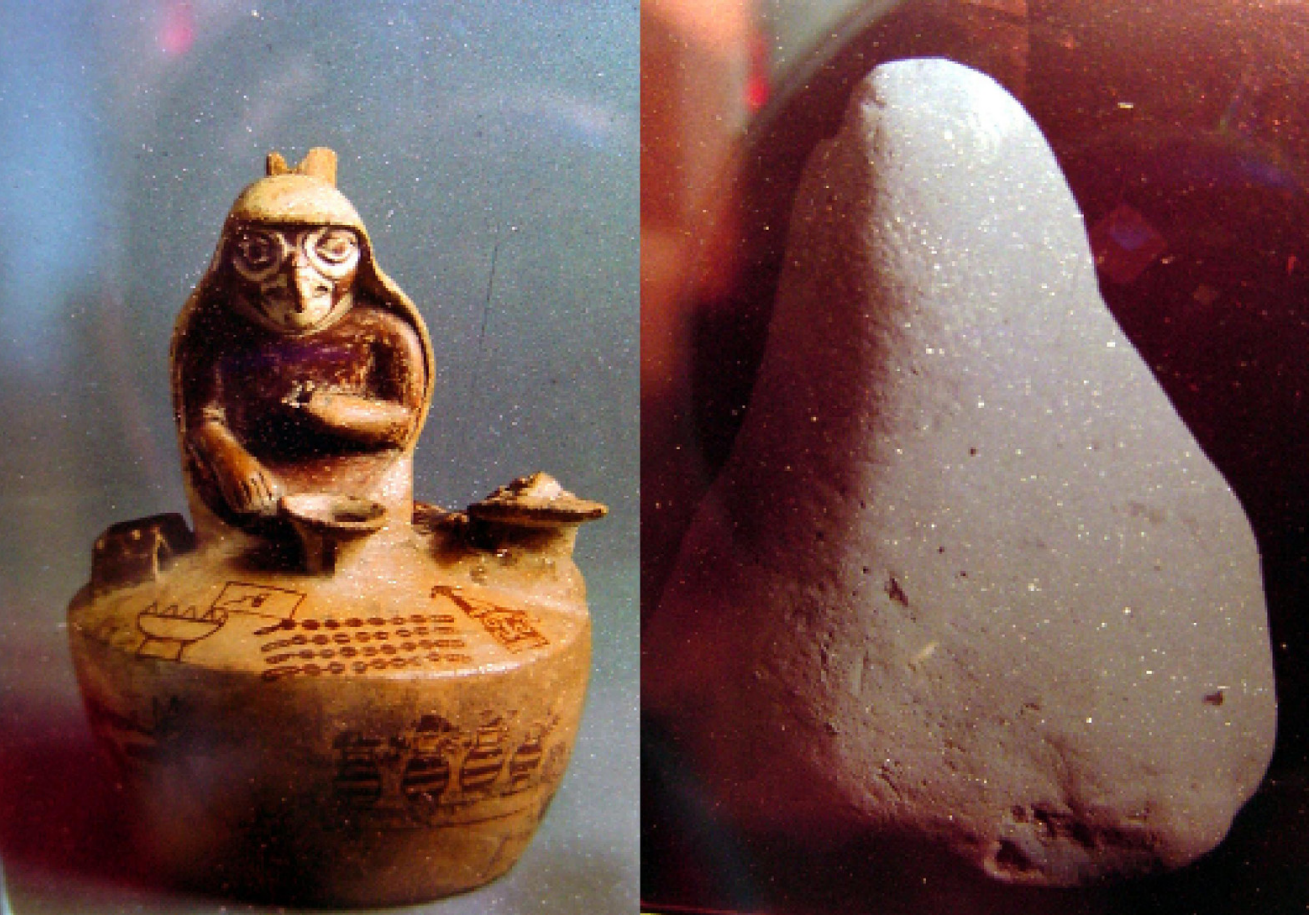
Cultural history of curanderismo: Moche ceramic showing a *curandera *in the form of an owl (left) and a pre-Columbian limestone cone used by contemporary *curanderos *(right).

In spite of the fact that Northern Peru is what Peruvian anthropologist Lupe Camino calls the "health axis," of the old Central Andean culture area stretching from Ecuador to Bolivia [22], little ethnobotanical and ethnomedical research has been published on the rich shamanic lore found here. The traditional use of medicinal plants in this region, which encompasses in particular the Departments of Piura, Lambayeque, La Libertad, Cajamarca, and San Martin (Fig. 1) dates as far back as the first millennium B.C. (north coastal Cupisnique culture) or at least to the Moche period (AC 100–800, Fig. 2), with healing scenes and healers frequently depicted in ceramics.

**Figure 3:**
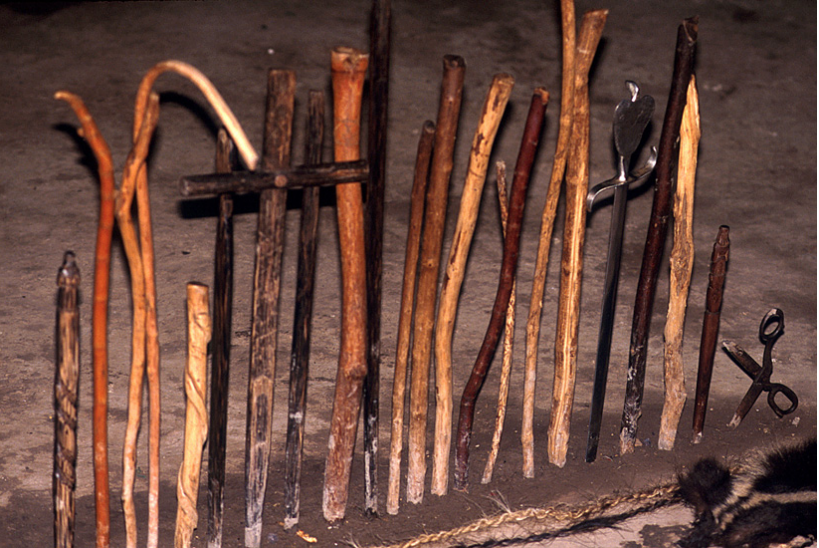
Section of a traditional Peruvian *mesa*.

**Figure 4:**
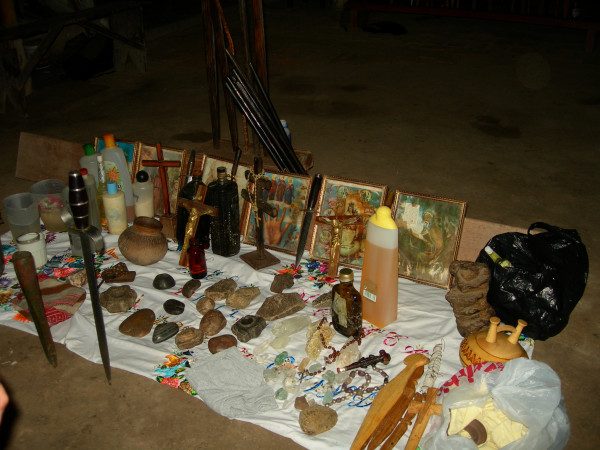
Section of a traditional Peruvian *mesa*.

Healing altars (*mesas*) in Northern Peru often follow the old tradition by including all kinds of "power objects," frequently with a "pagan" background. Objects such as seashells, pre-Columbian ceramics, staffs, stones, etc. are very common on Peruvian *mesas*, and are blended with Christian symbols such as crosses and images of saints (Figs. 2, 3 and 4).

Treatments are most often performed in the homes of the individual healers, who normally have their *mesas *(healing altars) set up in their backyards. Healers also treat patients at altars and consultation chambers (*consultorios*) at their homes, at sacred sites in the countryside, or at sacred lagoons high up in the mountains. A curing ceremony normally involves purification of the patient by orally spraying blessed and enchanted herbal extracts on the whole body to fend off evil spirits and by nasal ingestion of tobacco juice and perfumes.

Patients are cleansed by spraying them with holy water and perfumes, and baths or "Spiritual Flowerings" (*baños de florecimiento*) are very important components of the healing tradition. In most cases the cleansing of the patients involves the nasal ingestion of tobacco juice and perfumes, and extracts of Jimson weed (*Datura ferox*), *Brugmansia *spp., and tobacco are used to purify the patients.

While the incantations used by healers during their curing sessions include Christian components (e.g. the invocation of Christ, the Virgin Mary, and any number of saints), references to Andean cosmology (e.g., to the *apus *or the spirits of the mountains) are very common. The use of guinea pigs as diagnostic instruments is standard in Northern Peru.

Early ethnobotanically oriented studies focused mainly on the famous "magical" and "mind altering" flora of Peru. A first study on "*cimora*" – another vernacular name for the San Pedro cactus (*Echinopsis pachanoi*), dates back to the 1940's [23]. The first detailed study on a hallucinogen in Peru focused also on San Pedro, and tree datura (*Brugmansia *spp.) [24-26]. A variety of works on these species followed [27-30]. Coca (*Erythroxylon coca*) also attracted early scientific attention [31-35], as did the Amazonian Ayahuasca (*Banisteriopsis caapi*) [36-39]. Chiappe and Millones [40] were the first to attempt an overview on the use of hallucinogens in shamanistic practices in Peru. More comprehensive accounts are provided by [41-43].

In his classical study of "Uña de Gato", Peru's leading advocate for traditional medicine, and former director of the Instituto Nacional de Medicina Tradicional del Ministerio de Salud, Fernando Cabieses [44, p 34, 45–47] points out that the work of the Peruvian scholars Hermilio Valdizán and Angel Maldonado [21] was the pioneering effort in studying traditional medicine, leading to the emergence of medical anthropology nearly five decades later. In the interim the botanical exploration of the Peruvian flora and medicinal plants in particular included studies by Yakovleff [45] Weberbauer [46], Towle [47] and Valdivia [48]. Most authors [49-55] focused on Quechua herbalism of the Cusco area. Other comprehensive studies centered on the border region of Peru and Bolivia around Lake Titicaca [56-59] and the Amazon [60-64]. Northern Peru, in contrast, has always been in the shadow of these more touristically important regions, and very few studies have been conducted to date [65-68].

During the 1970s the World Health Organization (WHO) was very proactive in advocating the integration of traditional medicine into public health programs in Third World countries. This culminated in the Alma Ata Declaration of 1978, which proclaimed "health for all in the year 2000" [69]. Cabieses [44] describes his struggles to implement the UN tenets in Peru, together with Carlos Alberto Seguin [70,71] who advocated the incorporation of traditional folk psychotherapy into the modern institutional framework. In 1979 they organized the First World Congress of Traditional Medicine. As a result, Cabieses and Seguin were nearly expelled from the prestigious Colegio Médico del Perú, the counterpart of the American Medical Association, and Peru's Minister of Public Health declined the invitation to participate in the inaugural ceremonies of the event.

In 1988, 4000 participants from 41 countries attended the Second Congress, and the Minister of Public Health, the Dean of the Colegio Médico, and the Mayor of Lima all participated in the inauguration ceremony, along with a long list of university authorities. Published acts of the congress included important contributions on the medicinal flora of Peru [72,73]; and [74] for the Southern Andes. Subsequent publications of note include [53,56] for the southern highlands and [62,63] for the Peruvian Amazon region.

While he was director of the National Institute of Traditional Medicine, Dr. Cabieses was instrumental in coordinating a network of 16 ethnobotanical gardens in Peru, which included the cultivation of medicinal plants used by traditional herbalists. He also facilitated scientific research on traditional medicine building a large database of herbal plant uses. The subsequent administration discontinued these innovative programs and erased the database.

### Issues in ethnobotany

Moran, King, and Carlson [75] trace the emergence of biodiversity prospecting in the decade after the Convention on Biological Diversity (CBD) was signed.

For biodiversity-rich developing countries the most critical element in the CBD is sovereignty over bioresources, since the treaty recognizes right of nation states to regulate and charge outsiders for access to their biodiversity. The sovereignty component is meant to replace the "common heritage" paradigm, which provides unrestricted access to biological resources. Ideally this paradigm shift is supposed to balance the way in which all involved interest groups can gain from biodiversity use by recognizing the economic, sociocultural, and environmental values of bioresources and the cost of their preservation.

In the time since the CBD was initiated, few of the 178 signatory nations have introduced legislation requiring benefit sharing for outside commercial access to their national bioresources, although some suggestions for implementation of the CBD have been brought forward [76,77]. The U.S. and Peru are two non-activist countries. The U.S. never even signed the treaty while Peru's legislation does not enable mechanisms to conserve biodiversity. Recent environmental laws are just as toothless.

Despite the lukewarm response to the CBD, the global shift in awareness concerning tropical deforestation provided an opportunity for ethnobotanists to assert that everyone has an interest in preserving rainforests because they might contain compounds that could cure cancer, HIV-AIDS, and other diseases, as documented by [78-83]. Income derived from the marketing of traditional medicinal knowledge was seen as an instrument to alleviate poverty and to finance conservation efforts [78,84-86]. However, within a few years, ethnobotany – initially seen as an instrument that could help to salvage declining traditional knowledge and biodiversity – had simply become an instrument of theft and "biopiracy" for its critics [78].

Moran, King, and Carlson [75, p. 508–509] discuss the irony in this situation, indicating the fact that the majority of the biotech industry is not involved in bioprospecting, since most companies favor the use of cheaper and faster synthetic technologies over exploring for natural products. Nonetheless, biotechnology spawns ethical, social, and legal debates at the margins of pharmaceutical bioprospecting, including the collaboration between big business and big science, the ethics of genetic engineering, and the patentability of life forms as well as ideas about genetics and racism, culture and ethnicity. However, it is significant to note that, since the inauguration of the CBD, no pharmaceutical bioprospecting product developed by using traditional knowledge has generated an economic profit. Also, only a small number of bioprospecting research expeditions begin by using ethnobotany as a discovery methodology, with the work soon evolving into economic botany as the laboratory focus shifts to the plant's chemistry, biological activity, and pharmacology/toxicology. During drug discovery, active chemical components are isolated, often modified, and patented. Patented information then becomes a commodity in itself.

Manek and Lettington in *Cultural Survival *[87] point out that by focusing on indigenous knowledge as it relates to the environment, the CBD managed to sidestep some of the more politically charged aspects of the intellectual property rights (IPR) issue. The greatest impact on concerns over indigenous and local-community rights can be traced to the mercurial rise of biotechnology on the international trade front and the 1995 version of the Word Trade Organization (WTO) Agreement on Trade Related Aspects of Intellectual Property Rights (TRIPS). These two factors have created a large potential market for indigenous and local knowledge and resources, while at the same time raising concerns about the risk that these resources will be misappropriated. Thus this knowledge is receiving increasing international attention in terms of its relationship to human rights as well as its relevance to modern science. The situation has created opposing pressures calling for the rights of local and indigenous peoples on the one hand and further exploitation of their knowledge on the other. [75,87,88] indicate that the biggest problem with the orthodox intellectual property system is its focus on material aspects of knowledge at the expense of the cultural. They advocate recognition of alternative worldviews in the formulation of new indigenous knowledge rights that are localized, relevant, pertinent, and effective.

In their article in *Cultural Survival *Bannister and Barrett [89] contend that bioprospecting is a form of economic botany that can run contrary to the ethnobotanical objectives of protecting biological and cultural diversity. The economic focus of this activity highlights issues concerning indigenous rights, cultural knowledge, and traditional resources – areas in which current intellectual property protection regimes are inadequate and inappropriate. However, indigenous communities are increasingly forced to employ intellectual property rights to protect these resources. Protection issues ought to be addressed well before the point at which employing intellectual property mechanisms seems to be the only alternative. Significant control lies at the point of decision about publication and dissemination of knowledge to the wider community, which raises important questions about facilitating the appropriation of cultural knowledge. The authors [89, p. 10] advocate a more "precautionary" approach to ethnobotanical inquiry in assisting indigenous communities in protecting cultural heritage and intellectual property rights.

Probably the major concern in many traditional communities is that their spiritual legacies will be profaned by a secularized and consumer-driven outside world. Often, however, legitimate economic considerations also play a role in the defensive reactions of these societies to the well-intended but naïve desire of the academic world to place its findings in the public domain. Greaves [88] and others [89] warned that the downside in this approach is that a "colonialized archive" can become easily mined for clues in the search for new drugs without the inconvenience of fieldwork or benefit sharing.

Although acknowledging genuine concerns about neocolonialism and biopiracy, we would submit that each situation has to be considered on its own merits, especially with regard to its specific cultural context. A first step in the evaluation process should involve the important distinction between "indigenous peoples" and "local communities" [75, p. 518–519]. The latter for the most part are farmers who speak the national language, practice the majority religion, and identify with the nation-state, especially with regard to their socioeconomic aspirations, whereas the former tend to be tribal and/or ethnic minorities, who seek collective rights and self-determination for their biological and cultural resources. It is often the case that in local communities traditional knowledge and resources are undocumented and in danger of disappearing, as their members continue to adapt to modernization and globalization. In cases such as these successful ethnobotanical intervention requires a methodology that combines "salvage ethnography" with "rapid assessment". This is the methodology that we are applying in Peru, which provides the rationale for the present paper.

India provides a positive example of the proactive application of this rationale. By taking advantage of the "novelty" criterion in international patent law with regard to the documentation of Ayurvedic medicine and other traditional practices, millennial Sanskrit texts, as well as modern publications are included in a traditional knowledge database, which is subsequently provided to patent agencies. The expectation is that, by placing the knowledge about long-term cultural precedents for traditional uses in the public domain, this research will prove that contemporary patent applications derived from local medicinal knowledge lack originality, i.e., they are not "novel" enough to qualify as inventions warranting protection under international patent law, and are thus not patentable.

## Materials and methods

### Plant collections

Plants were collected in the field, in markets, and at the homes of traditional healers (*curanderos*) visited in August-September 2001, July-August 2002, July-August 2003, June-August 2004, July-August 2005 and July-August 2006. The specimens are registered under the collection series "RBU/PL," "ISA," "GER," "JULS," "EHCHL," "VFCHL," "TRUBH," and "TRUVANERICA," depending on the year of fieldwork and collection location (see Additional file 1).

Vouchers of all specimens were deposited at the Herbario Truxilliensis (HUT, Universidad Nacional de Trujillo), and Herbario Antenor Orrego (HAO, Universidad Privada Antenor Orrego Trujillo). In order to recognize Peru's rights under the Convention on Biological Diversity, especially with regard to the conservation of genetic resources in the framework of a study treating medicinal plants, the identification of the plant material was conducted entirely in Peru. No plant material was exported in any form whatsoever.

### Nomenclature

The nomenclature of plant families, genera, and species follows the Catalogue of the Flowering Plants and Gymnosperms of Peru [20]. The nomenclature was compared to the TROPICOS database. Species were identified using the available volumes of the Flora of Peru [90], as well as [91-93], and reference material in the herbaria HUT and HAO. Complete species names with author names for all species are given in Additional File 1.

### Ethnobotany

Ethnobotanical data were collected from plant sellers while purchasing plant materials in local markets (mostly Mercado Mayorista and Mercado Hermelindas in Trujillo and Mercado Moshoqueque and Mercado Modelo in Chiclayo), by accompanying local healers (*curanderos*) to the markets when they purchased plants for curing sessions and into the field when they were harvesting. In addition, plants were collected by the project members in the field, and – together with the material purchased in the markets – taken to the homes of *curanderos *to discuss the plants' healing properties, applications, harvesting methodology, and origins. At the homes of *curanderos *the authors also observed the preparation of remedies and participated in healing rituals. Plant uses were discussed in detail with informants, after seeking prior informed consent from each respondent. Following a semi-structured interview technique [94,95], respondents were asked to provide detailed information about the vernacular plant name in Spanish or Quechua; plant properties (hot/cold); harvesting region; ailments for which a plant was used; best harvesting time and season; plant parts use, as well as mode of preparation and application; and specific instructions for the preparation of remedies, including the addition of other plant species. All interviews were carried out in Spanish, with at least one of the authors present. Both authors are fluent in Spanish, and no interpreter was needed to conduct the interviews.

Data on plant species, families, vernacular names, plant parts used, traditional uses and modalities of use were recorded and are given in Additional File 1.

### Informant consensus

Many of the species reported in this paper are widely known by *curanderos*, herb vendors, as well as the general population of the region, and are employed for a large number of medical conditions. One hundred fifty to two hundred plant species, including most of the introductions, are commonly sold in the local markets. Rare indigenous species are either collected by the healers themselves, or are ordered from special collectors or herb vendors. The same plants are frequently used by a variety of healers for the same purposes, with only slight variations in recipes. However, different healers might give preference to different species for the treatment of the same medical condition. All species found were well known to the healers and herb vendors involved in the study, even if they themselves did not use or carry the species in question. Many species were often easily recognized by their vernacular names by other members of the population. This indicates that these remedies have been in use for a long time by many people. The use of some species, most prominently "*San Pedro*" (*Echinopsis pachanoi*), "*Maichil*" (*Thevetia peruviana*) and "*Ishpingo*" (various species of *Nectandra*), can be traced back to the Moche culture (AC 100–800). Representations of these plants are frequently found on Moche ceramics, and the remains of some were found in a variety of burials of high-ranking individuals of the Moche elite, e.g. the tomb of the "Lord of Sipan."

## Results

### Indigenous nomenclature

The healers interviewed in Northern Peru belonged entirely to the Mestizo community. The naming of plant species follows three general patterns. Plant names already used by original indigenous populations are often maintained, although slightly modified. Plants similar to species already known, or with similar habitus, often receive the same name (transposition). In other cases, completely new names are created (neology) [96].

The vernacular names of the plants used in Northern Peru reflect the historical development of plant use in the region. Introduced species (e.g. *Apium graveolens *– Apio, *Foeniculum vulgare *– Hinojo), native species similar to species found in Spain (e.g. *Adiantum concinnum *– Culantrillo, *Matricaria frigidum *– Manzanilla), as well as species growing mostly in the coastal regions of the area (e.g. *Alternanthera porrigens *– Sanguinaria), are often addressed with names derived from Spanish roots. Plants from the mountain forests and especially the Andean highlands or the Amazon are often known by their Quechua names (e.g. *Pellaea ternifolia *– Cuti Cuti, *Amaranthus caudatus *– Quihuicha, *Banisteriopsis caapi *– Ayahuasca), and a few plant names can be traced back to Mochica roots (e.g. *Nectandra *spp. – Espingo). Van den Eynden observed similar patterns in Southern Ecuador [96], although her study focused only on edible species. Ninehundred thirty-eight vernacular names were recorded for 510 plant species. About one third if all names represented Quechua names or had Mochica roots, while 66.5% of all names were of Spanish origin or had at least Spanish components. In comparison, 41% of the verbacular names of edible plants in Southern Ecuador were found to be of Mestizo origin. More than half of the indigenous species carry only one vernacular name, with the remaining species carrying a variety of indigenous names, often derived from the same root. In comparison, almost 75% of the introductions are known by one name only. The slight differences in plant names indicate that the species have been used in the region for a long time, and that their names reflect small variations in the local dialects.

### Plant uses

A total of 510 taxa belonging to 250 genera and 126 families are now on record. Of these, 504 could be identified, most of them to the species level. A detailed overview of all plants encountered, their scientific and vernacular names, and all uses, is given in Additional File 1.

Four hundred thirty-three species (85%) were Dicotyledons, 46 (9%) Monocotyledons, 21 (4%) Pteridophytes and 5 (1%) Gymnosperms. Three species of *Giartina *(Algae) and one species of the Lichen genus *Siphula *were used. Four hundred twenty-two species (83%) were indigenous to Northern Peru, while 87 species (17%) were introductions. Many of the introduced species were medicinal plants that were brought in for the treatment of European diseases during colonial times (Table 1).

**Table 1:** Main plant groups used in Northern Peru and plant origin

	**Number of species**	
		%
**Dicotyledoneae**	434	85
**Monocotyledoneae**	46	9
**Pteridophyta**	21	4
**Gymnospermae**	5	1
**Algae**	3	0.7
**Lichenes**	1	0.3
**Total**	**510**	**100**
		
**Indigenous**	**424**	**83**
**Introduced**	**86**	**17**

The families best represented were Asteraceae with 69 species, Fabaceae (35), Lamiaceae (25), and Solanaceae (21). Euphorbiaceae had 12 species, and Poaceae and Apiaceae 11 species (Table 2).

**Table 2:** Plant families used in Northern Peru

**Family**	**Number of species**		**Family**	**Number of species**	
		**%**			**%**
ASTERACEAE	69	13.53	OLACACEAE	2	0.39
FABACEAE	35	6.86	OXALIDACEAE	2	0.39
LAMIACEAE	25	4.90	PINACEAE	2	0.39
SOLANACEAE	21	4.12	POLEMONIACEAE	2	0.39
EUPHORBIACEAE	12	2.35	POLYGALACEAE	2	0.39
POACEAE	11	2.16	PORTULACACEAE	2	0.39
APIACEAE	11	2.16	SALICACEAE	2	0.39
LYCOPODIACEAE	10	1.91	SMILACACEAE	2	0.39
CUCURBITACEAE	9	1.76	TILIACEAE	2	0.39
ROSACEAE	9	1.76	ZINGIBERACEAE	2	0.39
AMARANTHACEAE	8	1.57	ACANTHACEAE	1	0.19
MYRTACEAE	8	1.57	AMARYLLIDACEAE	1	0.19
PIPERACEAE	8	1.57	ANNONACEAE	1	0.19
RUTACEAE	8	1.57	AQUIFOLIACEAE	1	0.19
GENTIANACEAE	6	1.18	ARALIACEAE	1	0.19
BRASSICACEAE	6	1.18	ARAUCARIACEAE	1	0.19
ORCHIDACEAE	6	1.18	ARISTOLOCHIACEAE	1	0.19
CAMPANULACEAE	6	1.18	ASCLEPIADACEAE	1	0.19
PASSIFLORACEAE	6	1.18	ASPHODELACEAE	1	0.19
VERBENACEAE	6	1.18	BALANOPHORACEAE	1	0.19
AIZOACEAE	5	0.98	BERBERIDACEAE	1	0.19
ANACARDIACEAE	5	0.98	BETULACEAE	1	0.19
APOCYNACEAE	5	0.98	BIXACEAE	1	0.19
BORAGINACEAE	5	0.98	CHLORANTHACEAE	1	0.19
BROMELIACEAE	5	0.98	CHRYSOBALANACEAE	1	0.19
CLUSIACEAE	5	0.98	CRASSULACEAE	1	0.19
GERANIACEAE	5	0.98	CUPRESSACEAE	1	0.19
LAURACEAE	5	0.98	ELAEOCARPACEAE	1	0.19
MALVACEAE	5	0.98	EPHEDRACEAE	1	0.19
PLANTAGINACEAE	5	0.98	ERIOCAULACEAE	1	0.19
POLYPODIACEAE	5	0.98	ERYTHROXYLACEAE	1	0.19
SAXIFRAGACEAE	5	0.98	HIPPOCRATEACEAE	1	0.19
STERCULIACEAE	5	0.98	ILLICIACEAE	1	0.19
BIGNONIACEAE	4	0.78	ISOETACEAE	1	0.19
CYPERACEAE	4	0.78	JUGLANDACEAE	1	0.19
LILIACEAE	4	0.78	KRAMERIACEAE	1	0.19
MORACEAE	4	0.78	LECYTHIDACEAE	1	0.19
RUBIACEAE	4	0.78	LEMNACEAE	1	0.19
SCROPHULARIACEAE	4	0.78	LICHENES	1	0.19
VALERIANACEAE	4	0.78	LOGANIACEAE	1	0.19
ALGAE	3	0.59	LYTHRACEAE	1	0.19
CAPRIFOLIAEAE	3	0.59	MALESHERBIACEAE	1	0.19
CHENOPODIACEAE	3	0.59	MALPIGHIACEAE	1	0.19
CONVOLVULACEAE	3	0.59	MENISPERMACEAE	1	0.19
ERICACEAE	3	0.59	MUSACEAE	1	0.19
MONIMIACEAE	3	0.59	MYRICACEAE	1	0.19
PHYTOLACCACEAE	3	0.59	MYRISTICACEAE	1	0.19
POLYGONACEAE	3	0.59	OLEACEAE	1	0.19
URTICACEAE	3	0.59	PAPAVERACEAE	1	0.19
MELASTOMATACEAE	3	0.59	PROTEACEAE	1	0.19
ONAGRACEAE	3	0.59	PUNICACEAE	1	0.19
ADIANTACEAE	2	0.39	RANUNCULACEAE	1	0.19
ALSTROEMERIACEAE	2	0.39	SAPOTACEAE	1	0.19
ARECACEAE	2	0.39	THEACEAE	1	0.19
BURSERACEAE	2	0.39	THELYPTERIDACEAE	1	0.19
CACTACEAE	2	0.39	THYMELEACEAE	1	0.19
CAPPARIDACEAE	2	0.39	TROPAEOLACEAE	1	0.19
CARICACEAE	2	0.39	TYPHCEAE	1	0.19
CARYOPHYLLACEAE	2	0.39	ULMACEAE	1	0.19
DIOSCOREACEAE	2	0.39	VIOLACEAE	1	0.19
DIPSACACEAE	2	0.39	VITACEAE	1	0.19
EQUISETACEAE	2	0.39	XYRIDACEAE	1	0.19
LINACEAE	2	0.39	ZYGOPHYLLACEAE	1	0.19
LORANTHACEAE	2	0.39	INDET.	6	1.17
NYCTAGINACEAE	2	0.39	**TOTAL**	**510**	

#### Medicinal

Five hundred ten plants with medicinal properties were registered in Northern Peru. The same species was often used for various medical conditions and applied in different ways for the same condition. For example, nervous disorders might be treated using different parts of a plant in different applications, e.g., topical (as a poultice or bath), oral (ingestion of plant extracts), and by supplying the patient with a "*seguro*," a bottle with herbs and perfumes that serves as a protecting charm. Two thousand four hundred ninety-nine different uses were registered for the 510 species encountered. Two hundred seventy-eight different medical conditions were recorded. Most plants were used for the treatment of multiple ailments. The large variety of conditions is grouped into 72 main categories (Table 3).

**Table 3:** Plant uses in Northern Peru

**Use**	**Number of uses**		**Number of species used**	
		**%**		**%**
**Magical/Ritual healing**	682	27.3	207	40.4
**Respiratory**	233	9.3	95	18.5
**Psychosomatic/Nerves**	176	7.0	98	19.1
**Kidneys/Urinary**	111	4.4	85	16.6
**Rheumatic – Arthritis, Muscle pain**	103	4.1	45	8.8
**Female – Infections of uterus, vagina etc.**	100	4.0	66	12.9
**Intestinal – Liver**	77	3.1	61	11.9
**Skin**	75	3.0	40	7.8
**Stomach**	70	2.8	51	9.9
**Blood**	68	2.7	44	8.6
**Heart**	68	2.7	42	10.1
**Inflammation**	63	2.5	59	11.5
**Intestinal – Colic, gases, constipation**	52	2.1	29	5.7
**Wounds**	47	1.9	43	8.4
**Female – birth and reproduction issues**	39	1.6	25	4.9
**Diabetes**	32	1.2	33	6.4
**Inflammation (internal and intestines)**	31	1.2	33	6.4
**Bones (fractures, sprains)**	26	1.0	13	2.5
**Male – Prostate, Impotence**	24	0.96	23	4.5
**Fever**	23	0.92	17	3.3
**Cancer**	22	0.88	22	4.3
**Intestinal – Laxative and purgative**	21	0.84	19	3.7
**Haemorrages**	20	0.80	12	2.3
**Hair**	20	0.80	17	3.3
**Intestinal – Diarrhea**	18	0.72	17	3.3
**Intestinal – Gallbladder**	18	0.72	18	3.5
**Sex – Aphrodisiac, potency**	18	0.72	12	2.3
**Weight loss**	16	0.64	15	2.9
**Bacterial infections**	15	0.60	7	1.4
**Cholesterol**	14	0.56	14	2.7
**Viral infection**	14	0.56	11	2.1
**Food**	12	0.48	12	2.3
**Inflammation of tonsils**	11	0.44	7	1.4
**Parasites**	11	0.44	11	2.1
**Hallucinogen/Enhancement of visions**	10	0.40	7	1.4
**Animal bites**	9	0.36	5	1.0
**Cysts**	9	0.36	9	1.8
**Eye sight**	9	0.36	6	1.2
**Allergies**	8	0.32	8	1.6
**Blood – High blood pressure**	8	0.32	7	1.4
**Female – birth control**	8	0.32	8	1.6
**Headache**	8	0.32	6	1.4
**Cleansing internal and external**	7	0.28	4	0.8
**Fungus**	7	0.28	5	1.0
**Pain**	7	0.28	5	1.0
**Sharp pain in the body**	7	0.28	5	1.0
**Teeth – Inflammation of molars**	7	0.28	5	1.0
**Memory/Brain**	6	0.24	6	1.2
**Contussion**	5	0.20	3	0.6
**Hangover**	5	0.20	3	0.6
**Bad breath**	4	0.16	4	0.8
**Detoxification (alcohol and drugs)**	4	0.16	4	0.8
**Haemorrhoids**	4	0.16	4	0.8
**Paralysis**	4	0.16	1	0.2
**Blood – Anemia**	3	0.12	3	0.6
**Blood – Low blood pressure**	3	0.12	3	0.6
**Ears and hearing problems**	3	0.12	3	0.6
**Internal bleeding**	3	0.12	3	0.6
**Varicose veins**	3	0.12	3	0.6
**Alertness**	2	0.08	2	0.4
**Nosebleed**	2	0.08	2	0.4
**Weight – gain**	2	0.08	1	0.2
**Abscesses**	1	0.04	1	0.2
**Anesthetic**	1	0.04	1	0.2
**Antiseptic**	1	0.04	1	0.2
**Anus and vaginal cysys and pimples**	1	0.04	1	0.2
**Cramps**	1	0.04	1	0.2
**Mouth bitterness**	1	0.04	1	0.2
**Sarna**	1	0.04	1	0.2
**Waking a person who has fainted**	1	0.04	1	0.2
**TOTAL**	**2499**	**99.46**		

In the following, the total number of uses/applications and the number of species used are given, rather than only the number of plant species used to treat a condition, in order to emphasize the importance of the treatment of specific conditions.

The highest number of species (207, 40.4%) is used for the treatment of "magical/ritual" ailments like *Mal aire *(bad air; illness caused by spirits who influence passing adults), *mal viento *(bad wind, similar to mail aire but affecting mostly children), *susto *and *espanto *(fright, caused by an astounding event in life or environment), *mal ojo *(evil eye, illness mainly in children caused by persons with pervasive look) and *envidia *(envy, illness of adults caused by envy of other persons), with 682 (27.3%) of all conditions. Respiratory problems (95 species, 18.5%) were mentioned as 233 (9.3%) of all uses; 98 species (19.1%) are used to treat psychosomatic and nervous system problems, with 176 applications (7%). Kidney and urinary tract disorders are treated with 85 species (16.6%), with 111 conditions (4.4%). Rheumatic and arthritic symptoms are mentioned in 103 uses (4.1%), with 45 species (8.8%) used for treatment. Infections of female organs are treated with 66 species (12.9%), and comprised 100 (4.4%) of all conditions. Table 3 gives an overview of the main illness categories treated.

### Magical/ritual healing

*Mal aire *(bad air), *mal viento *(bad wind), *susto *and *espanto *(fright), *mal ojo *(evil eye) and *envidia *(envy) are seen as very common illnesses in Andean society. Causes include sudden changes in body temperature, any kind of shock, spells cast by other people, poisoned food, etc. Medicinal problems caused by outside influences were reported in a wide variety of studies [57,96]. The Western concept of "psychosomatic disorders" comes closet to characterizing these illnesses. These illness categories are deeply rooted in Andean society, and Western medicine does not offer efficient alternatives to traditional treatment. This might explain why this category has still such outstanding importance.

Six hundred eighty-two applications (27.3% of all uses) fall into the "magical/ritual" category. Two hundred seven plant species (40.4% of all species encountered) were named for treating these disorders. In addition, seven species (1.4%) were used as hallucinogens in curing ceremonies.

Treatment in many cases involved the participation of the patient in a cleansing ceremony or *limpia*. This could either be a relatively simple spraying with perfumes and holy water, or an all-night ceremony involving the healer's curing altar (*mesa*). In the days after an all- night ceremony, patients are normally treated with a f*lorecimiento *(flowering bath) in order to relieve them of any remaining adversary symptoms or spirits. In addition, patients frequently receive *seguros *(herbal amulets) for protection against further evil influences and for good luck. *Seguros *are flasks filled with powerful herbs, as well as perfumes, pictures of saints, and the hair and fingernails of the patient.

The enormous number of plant species used for the treatment of psychosomatic disorders indicates that the *curanderos *of Northern Peru are valued specialists who are consulted mainly for these conditions. This is all the more interesting since Western medicine has still not found efficient treatments for psychosomatic disorders. The plant species used for "magical/ritual" disorders come mostly from the high Andes, especially from the vicinity of sacred lakes, since plants from those regions are regarded as especially powerful. This links the present day curing practices directly to ancient Andean cosmology. The use of purgatives and laxatives, to literally "expel" evil spirits is also very common.

### Nerves and psychosomatic problems

The enormous role that *curanderos *play in the area of treatment of psychosomatic and nervous system problems become even more apparent when considering that 176 uses (7%) involve the treatment of nervous system disorders like depression, anxiety, insomnia, etc. A total of 98 species (19.1%) was employed for this category. Some of the plants used, e.g., *Valeriana *spp. are used worldwide for the treatment of nervous disorders.

### Respiratory system

Respiratory system problems, like, the common cold, flu, bronchitis and asthma represent the most "tangible" illnesses treated by healers in Northern Peru. Two hundred thirty-three uses (9.3%) fall into this category. The damp conditions in local homes, leading to high mold counts, as well as insufficient air circulation account for the prevalence of these conditions. Many houses in rural areas still have open stoves, with smoke causing constant irritation to the pulmonary system. *Curanderos *use 95 plant species (18.5%) for respiratory problems.

### Urinary system (Kidneys, Bladder)

Disorders of the urinary system include kidney and bladder infections and kidneystones. Altogether 111 applications (4.4%) focused on the urinary system, with 85 plant species (16.6%) used. Some of the species employed, e.g., *Chanca Piedra*, literally "Stonebreaker" (*Phyllanthus *spp.) have already entered the international market.

### Rheumatic problems

The housing conditions already described, as well as difficult working conditions, lead to a wide spectrum of muscular-skeletal disorders, including rheumatism, arthritis, bone- and muscle-pain. One hundred three applications (4.1%) with 45 species (8.8%) used fall into this illness category. Treatment involves the application of a poultice to the affected body part. Willow (*Salix *sp.), well known for its content of acetacetylic acid, is used orally as an analgesic.

### Internal organs (Liver, Gallbladder, Diarrhea, Colic)

Disorders of internal organs fall far behind the most commonly treated medical conditions. This is another indication that *curanderos *in Northern Peru are to a large extent specializing in the treatment of psychosomatic disorders, and that "bodily" illnesses are treated more as a sideline. Internal organ problems treated include liver (77 applications, 3.1%; 61 species, 11.9%); stomach problems, including ulcers (70 applications, 2.8%; 51 species, 9.9%); colic (52 applications, 2.1%; 59 species, 11.5%); digestive tract inflammations (31 applications, 1.2%; 33 species, 6.4%), diarrhea (18 applications, 0.72%; 17 species, 3.3%), and gallbladder problems, including stones (18 applications, 0.72%; 18 species, 3.5%). The cleansing of the digestive system trough enemas (7 applications, 0.28%; 4 species, 0.8%) and by employing laxatives/purgatives (21 applications, 0.84%; 19 species, 3.7%) was also observed.

### Gynecological problems

Gynecological problems are among the most important medical conditions treated by *curanderos*, independent of the gender of the healer. Infections of ovaries, uterus, and vagina as well as post partum infections were very common conditions for which women sought the help of healers. Infections of this kind involved 100 applications (4%), but 66 species (12.9% of the total) were used for treatment. Furthermore, 39 uses (1.6%; 25, 4.9%) involved facilitation of childbirth, such as easing of dilation. The same species were often used to ease menstrual cramps and to regulate the menstrual cycle.

Birth control, female fertility, and abortion were only mentioned in 8 applications (0.32%), with 8 different species (1.6%) used, only one of which (*Ruta graveolens*) was used to induce abortions.

### Skin problems

Skin infections, either fungal or bacterial, as well as sunspots, moles, pockmarks, and malnutrition blemishes can be observed frequently in Northern Peru. Traditional healers are consequently consulted to treat these conditions. Seventy-five applications (3%) involved skin problems, and 40 species (7.8%) were used. Fungal infections are particularly difficult to treat in the context of Western medicine, and the use of plants to alleviate such infections is thus of particular interest.

### Heart and circulatory system

Traditional healers are frequently consulted to treat heart problems and disorders of the circulatory system. Typical heart conditions, including heart pain involved 68 applications (2.7%), for which 44 species (8.6%) were used. Blood pressure issues were rather insignificant, with high blood pressure treated in 8 applications (0.32%; 7 species, 1.4%), and low blood pressure in 3 applications (0.12%; 3 species, 0.6%). Interestingly, *Erodium cicutarium *was used to treat both conditions.

Most treatments of the circulatory system involved the purification of the blood in order to improve the general condition of the patient. Sixty-eight applications (2.7%) involve such blood purifications, and 44 species (8.6%) were used for this purpose.

### Weight management/cholesterol

The fashionable concept of "weight management" and conditions related to obesity has entered into the domain of Peruvian healers. Diabetes, especially in overweight patients, occurs as a prominent medical condition with 32 applications (1.2%) and 33 species (6.4%) used for treatment. The high incidence of diabetic conditions seems to point towards a change in lifestyle and nutrition by the local population. All healers readily acknowledge the negative influence of high cholesterol levels, and 11 plant species (2.1%) were used specifically to lower cholesterol. Sixteen applications (0.64%) with 15 species (2.9%) involved weight loss therapies, while plants used for weight gain were insignificant (2 applications, 0.08%; 2 species, 0.4%).

### Inflammation

General inflammation of the body was mentioned in 63 applications (2.5%), and 59 plant species (11.5%) were used for such conditions. In addition, throat and tonsil infections were treated with 7 species (1.4%).

### Wounds and hemorrhages

Wound infections and bleeding resulting from accidents are very common in the Northern Peruvian work environment, and are a major concern especially in rural areas. Forty-seven applications involved wound treatment. Although this represents only 1.9% of all plant uses, 8.4% of all plants (43 species) were used for the treatment of wounds. An additional 12 species (2.3%) were used in 20 applications (0.8%) that involved the treatment of bleeding and hemorrhages.

### Bones

Twenty-six (1%) of all plant uses included the treatment of fractures, sprains and the like, with 13 species (2.5%) used for this purpose.

### Male problems (Impotence, Prostate, Hair loss)

Typical "male" problems like prostate inflammations and disorders, impotence, and hair loss had a relatively prominent role in the treatments observed. Twenty-four applications (0.96%) with 23 species (4.5%) used involved prostate inflammations and problems in urinating. "Hair loss" was mentioned in 20 applications (0.8%), with 17 species (3.3%) used for treatment. Finally, 18 applications (0.72%) of 12 species (2.3%) focused on the treatment of male impotence, on the improvement of potency, or the plants were simply used as aphrodisiacs.

### Fever

"Fever" included a variety of conditions, from fevers accompanying flu, to fever as a result of malaria. Plants were employed for 23 applications (0.92%), with 17 species (3.3%) used. Malaria was recognized as a parasitic infection, and treated accordingly, while other plant species were used to treat fever as a symptom, mainly focusing on lowering body temperature.

### Cancer and tumors

Various cancers and tumorous conditions are also treated by *curanderos*. Treatment of such cases often involves a single species at a time, with a total of 22 plant species (4.3%) used in 22 applications (0.88%). The use of plant species in this field could provide particularly interesting leads in medicinal development.

### Infection (Bacterial and Viral, Parasites)

Infections caused by bacteria, viruses, and various parasites are common in many developing countries. Bacterial infections treated included cholera, tuberculosis, and gangrene, with 14 applications (0.56%; 11 species, 2.1%) while viral infections were mostly related to dengue fever, yellow fever and measles (15 applications, 0.6%; seven species, 1.4%). Intestinal, urinary tract and female organ infections are mentioned in the respective paragraphs.

Parasites like amoebas, plasmodia, and worms were mentioned in 11 applications (0.44%), and 11 different species (2.1%) were employed for these conditions.

### Pain

Seven applications (0.28%) with five species (1%) involved the treatment of general pain, intense body pain (e.g., caused by dengue fever), as well as tooth pain and the follow up after extraction.

### Brain

Memory loss and confusion, as caused by old age were mentioned in 6 applications (0.24%), and treated with six plant species (1.2%).

### Other uses

Rare disorders treated included contusion, hangover (five uses, 0.2%; three species, 0.6%); animal bites (snake bites, rabies) (nine uses, 0.36%; five species, 1%); Eye problems (nine uses, 0.36%; nine species, 1.8%); cysts (nine uses, 0.36%; six species, 1.2%); headache (eight uses, 0.32%; six species, 1.4%); bad breath, detoxification (drug and alcohol abuse); hemorrhoids (four uses, 0.16%; four species, 0.8%); paralysis (four uses, 0.16%; one species, 0.2%); anemia, ear and hearing problems, internal bleeding, varicose veins (three uses, 0.12%; three species, 0.6%); alertness, nosebleeds (two uses, 0.8%; two species, 0.4%); abscesses, anesthetics, anal and vaginal pimples, antiseptics, cramps, mouth bitterness, sarna, and waking a person who has fainted (one use, 0.04%; one species, 0.2%).

#### Parts of medicinal plants used and mode of application

Northern Peruvian *curanderos *prefer to use either the leaves (in 25% of all uses) or the whole plant (24%) for the preparation of their remedies. In 19% of the cases the stems of the plants were used, most commonly together with the leaves. Flowers (10%), seeds (7%), fruits and roots (4% each), bark (3%), fruit peel (2%), and latex and wood (1% each) were only used for a small number of preparations (Table 4).

**Table 4:** Plant part used for medicinal purposes

**Plant Part**	**Number of uses**	
		**%**
**Leaves**	191	25
**Whole plant**	184	24
**Stems**	146	19
**Flowers**	73	10
**Seeds**	55	7
**Fruit**	31	4
**Root**	28	4
**Bark**	20	3
**Fruit peel**	12	2
**Latex**	9	1
**Wood**	6	1
	**755**	**100**

Almost two-thirds (64%) of the remedies employed in Northern Peru are prepared using fresh plant material. Many of the introduced species are cultivated in fields and gardens, but the majority of the indigenous species are collected wild. This indicates that a widespread system of plant collectors is needed to supply the fresh plant material needed in traditional medicine. Most healers agreed, however, that in most cases dried material could be used if fresh plants were not available. In 36% of all cases the remedies were prepared using specifically dried plant material. The main explanation for this was however, that the plant material had to be transported from other regions, and thus fresh material was not available (Table 5).

**Table 5:** Plant constitution

**Constitution**	**Number of uses**	
		%
**Fresh**	626	64
**Dry**	355	36
**TOTAL**	**981**	**100**

Healers in Northern Peru often employ very sophisticated mixtures of a variety of plants in their treatments. The use of single species for treatments was rare. Most commonly, plant material was boiled in water, or in some cases in sugarcane alcohol (*aguardiente*) to extract the active compounds. In some cases, plant material was macerated in cane alcohol or wine for longer periods of time, before use.

The *curanderos *all had strikingly exact recipes for treatment, with very specific quantities of plant material used to prepare remedies. These quantities did not differ greatly from one healer to another. Simultaneously, the amount of a specific remedy that was given to a patient was very similar among the different *curanderos*.

The most frequent way to administer remedies was to prepare a decoction and ingest it orally (52% of all uses), followed by application as a poultice (38%, plant crushed or boiled and applied). Seven percent of all plant uses entailed the preparation of a *seguro*, a bottle or small flask filled with plant material along with various perfumes. This amulet has to be carried by the patient at all times, or it is placed in the house and used for periodic blessings. *Seguros *contained anything from a handful to more than three-dozen different ingredients. In two percent of the plant uses the material was employed to fabricate charms, and in one percent of all applications the plant material was burned as incense, with the smoke inhaled for treatment (Table 6).

**Table 6:** Preparation and application methods for medicinal plants:

**Application**	**Number of uses**	
		**%**
**Oral**	429	52
**Topical**	315	38
**Seguro**	60	7
**Charm**	14	2
**Incense**	10	1
		
**TOTAL**	**828**	**100**

#### Food and spices

A variety of species normally used as food also had some medicinal applications, mostly as nutritional supplements to treat mineral and vitamin deficiencies and malnutrition, and were prepared and served as side-dishes or as ingredients of normal meals. Old Andean crops like Quinoa (*Chenopodium quinoa*), Kichwa (*Amaranthus caudatus*), Tarhui (*Lupinus mutabilis*) and Maca (*Lepidium meyenii*) – now globally used as a supplement – featured most prominently. Coastal species like Algarrobo (*Prosopis pallida*) were also used. Altogether 12 species (2.3%) were used in this way.

#### Ceremonial

Palm staffs (*Bactris *spp.) are still used as power objects on Northern Peruvian *mesas*.

#### Phytochemistry of Peruvian medicinal plants

If the botanical documentation of Peruvian medicinal plants has been neglected for a long time, investigations on the phytochemical composition of useful plants is lagging even further behind. Most studies on the phytochemistry of Peruvian plants concentrate on a few "fashionable" species that have been marketed heavily on a global scale, especially Maca (*Lepidium meyenii*), Sangre de Drago (*Croton lechleri*), and Cat's claw/Uña de Gato (*Uncaria tomentosa *and *Uncaria guianensis*). The number of other Peruvian plants for which at least limited phytochemical studies exist is still minuscule, and most efforts are fueled by the fads and fashions of the international herbal supplement market. Studies involving multiple species were initiated as late as 1990s [97,98], and are still the exception to the rule.

## Conclusion

Current research indicates that the composition of the local pharmacopoeia has changed since colonial times [14]. However, the overall number of medicinal plants employed seems to have increased. This indicates that the Northern Peruvian health tradition is still going strong, and that the healers and public are constantly experimenting with new remedies. One example of this is the sudden appearance of Noni (*Morinda citrifolia*) fruits and products in large quantities in plant pharmacies and markets in the region since 2005. This plant was not available before, but is heavily marketed worldwide. Peruvian sellers are clearly reacting on a global market trend and are trying to introduce this new species to their customers. This makes it obvious that local herbalists and herb merchants are carefully watching international health trends to include promising species in their own repertoire.

The use of hallucinogens, in particular the San Pedro cactus (*Echinopsis pachanoi*) is still a vital component in Andean healing practices, and has been around for millennia [22,66,99-102]. San Pedro can often be found in Cupisnique and Moche iconography [15,16]. Five hundred years of suppression of traditional healing practices by church, state institutions and Western medicine, starting in colonial times and continuing to manifest in the prejudices of contemporary national administrations have not managed to destroy this tradition. The use of San Pedro, together with additives like Angel's-Trumpet (*Brugmansia *spp.), Jimson-weed (*Datura ferox*), and tobacco, is still a central part of the curing ceremonies of healers in Northern Peru. Healers are in fact experimenting with new hallucinogens, and some northern *curanderos *have started to include decoctions of Ayahuasca (*Banisteriopsis caapi*) in their rituals.

The knowledge of medicinal plants is still taught orally, with no written record. An illustrated identification guide for the medicinal plants of Northern Peru and their uses, similar to our field guide for Southern Ecuador [19] will hopefully help to keep the extensive traditional knowledge of this area alive. However, Traditional Medicine is experiencing increasing demand, especially from a Peruvian perspective as indicated by the fact that the number of herb vendors, in particular in the markets of Trujillo, has increased in recent years. Also, a wide variety of medicinal plants from Northern Peru can be found in the global market. While this trend might help to maintain traditional practices and to give traditional knowledge the respect it deserves, it poses a serious threat, as signs of overharvesting of important species are becoming increasingly apparent.

Today the most serious threat to this millennial tradition is the destruction of medicinal plant habitats. Urban sprawl has already greatly altered the coastal plains around Trujillo and Chiclayo. Climatic change is threatening the mountain forest systems that are the source of many medicinal species. Most importantly, the high Andean ecosystems and sacred lagoons where many medicinally active species are found are in danger of being destroyed by large-scale mining activities [103].

## Declaration of competing interests

The author(s) declare that they have no competing interests.

## Authors' contributions

Both authors share the contributions to fieldwork, data analysis, and compilation of this manuscript.

## Supplementary Material

Additional File 1 Medicinal plant species of Northern Peru: Scientific and vernacular names, uses and preparation. The data provided represent the complete overview on all plants encountered: Scientific names, vernacular names, plant parts used, preparation and uses. Click here for file
