# Supplementary material for: Traditional medicinal plant use in Northern Peru: tracking two thousand years of healing culture
Source: J Ethnobiol Ethnomed. 2006 Nov 7;2:47. doi: 10.1186/1746-4269-2-47 (PMC1637095; doi:10.1186/1746-4269-2-47)
Supplement: Additional File 1 — Medicinal plant species of Northern Peru: Scientific and vernacular names, uses and preparation. The data provided represent the complete overview on all plants encountered: Scientific names, vernacular names, plant parts used, preparation and uses. [file 1746-4269-2-47-S1.pdf]

Additional file 1. Species encountered and used in Northern Peru

Family/Genus/Species	Indigenous name	Plant part used	Admin.	Preparation	Use	Coll. #
ACANTHACEAE						
Aphelandra cirsioides Lindau	Espina de Hoja	Whole plant, dried	Oral	2 Tbsp with 1l boiled water, 3 cups per day, 3-4 days.	Bronchitis	ISA40
ADIANTACEAE						
Adiantum concinnum Wild. ex H.B.K.	Culantrillo del Pozo, Culantrillo	Leaves and Stems, fresh or dried	Oral	20g per 1l of water for 5 minutes combined with Purenrosa, Lancetilla, Moradilla, Oregano. Drink three glasses per day for three days.	Blood purification, Hair loss, Menstrual regulation	VFCHL29, TRUBH17, RBU/PL265, JULS149
Pellaea ternifolia C. Chr.	Cuti Cuti, Cute Cute, Cuticuti, Cute-Cute Amarillo, Cuti Cuti Amarillo	Whole plant, fresh	Oral	5g per 1l of water, drink 3 times per day for 1 week.	Diabetes, Liver	EHCHL46, RBU/PL319, TruBH21, RBU/PL258, TRUBH21
AIZOACEAE						
Tetragonia crystallina L'Herit	Hierba de la Sonrisa, Señorita, Ulluco de Gentil, Hierba de la Señorita	Stems, Leaves, Flowers, fresh	1. Seguro<br>2., 3. Topical	1. Standard Seguro mixture, see below.<br>2. Poultice, chop the Leaf and Stems, apply 2 hours, 2 times per day.<br>3. Alternative mixture for Spiritual Flowering, see below.	1., 3. Happiness, Tranquility, Fragrance, Good luck, Spiritual Flowering, Protection, Good health, Good fortune, Good business<br>2. Inflammation of the molars	ISA133, RBU/PL360, ISA26, GER131
AMARANTHACEAE						
Alternanthera brasiliana (L.) Kuntze	Hierba del Oso, Veronica (Hembra), Moradilla de Cerro	Whole plant, fresh or dried	1. Oral<br>2. Topical	1. 5-10g per 1l water, mix with Muyaca, Huamanrripa, Brochamelia. 4 cups per day, 1-2 weeks.<br>2. Limpia or bath. 5g per 3l boiled water, also use with Hierba del Susto, Zanahoria de Gentil, Poleo de Gentil. 1-2 times per month.	1. Twisted bones, Bronchitis, Asthma, Bruises, Fractures, Bumps<br>2. Susto of the summit / Susto de la cumbre	RBU/PL275, JULS11, EHCHL78, ISA83
Alternanthera halmifolia (Lam.) Standley & Pittier	Paja Morada (Colores), Lenguetilla, Sanguinario, Lengua de Pajaro, Sanguinaria, Moradia, Moradilla, Hierba Morada	Whole plant, fresh	1., 3. Oral<br>2. Topical	1., 2. Boil 1l water, then add 10g Paja Morada. Combine with Malva, Pie de Perro, Cola de Caballo, Chacur, and Sombrerito. Drink the solution and use it as a wash. 3 times a day, as needed.<br>3. Boil 1l water, then add 10g total of Toronjil, Manzanilla, Romero, Hinojo, Chanca de Comida, Cascorade, and Membrillo. Let mixture sit for 2 to 3 minutes. Drink lukewarm 3-4 times per day during mealtimes, or as needed. Patient should drink cold solution. It is important to drink it at 6AM and at 6PM.	1., 2. Inflammation<br>3. Nerves, Heart, Anxiety, Heart disease, Relaxation	JULS85, JULS243, GER23

Family/Genus/Species	Indigenous name	Plant part used	Admin.	Preparation	Use	Coll. #
Alternanthera porrigens (Jacquin) Kuntze	Sanguinaria, Moradilla, Lancetilla	Whole plant, fresh or dried	1. Oral<br>2. Topical<br>3. Seguro	1. 5g per 1l, combined with Conchalagua, Moradilla, Colores, Lancetilla, Culantrillo, Hierba del Toro, and Zarza Parilla. Drink 3 times per day up to one year.<br>2. Alternative mixture for Spiritual Flowering, see below. Take 3 baths per month.<br>3. Standard Seguro mixture, see below.	1. Blood circulation, Warts, Blood coldnes, Allergies<br>2. Cleansing womb after childbirth, Fragrance, Luck in love and work, Bad Air / Mal Aire, Love, Business Relations, Protection, Good fortune, Good health<br>3. Good business, Protection, Good fortune, Good health	EHCHL142, ISA56, RBU/PL301, RBU/PL324, EHCHL93, GER117
Alternanthera villosa H.B.K.	Hierba del Oso	Flowers, Leaves and Stems, fresh or dried	1. Oral<br>2. Topical	1. Boil 20g of Hierba del Oso in 1/2 cup of water for 5 minutes. Drink cold, 1/8 cup 1 time only.<br>2. Bath mixture for Protection from evil, see below.	1. Bad Air / Mal Aire, Sorcery (protection from)<br>2. Protection from evil	GER63
Amaranthus caudatus L.	Quihuicha, Kiwicha	Seeds, dried	Oral	150g of the grain and 1l of water. Boil for 10 minutes or until grain is soft. Add Cinnamon, Apples, and Membrillo. Oral cup 1-2 times a day as necessary.	Nutrition supplement	JULS235
Amaranthus hybridus L.	Yuyo	Leaves and Stems, fresh	Oral	100g of Yuyo and 1/2 cup of water and boil for 5 minutes. Drink cold, 1/4 cup 2 times a day for 3 days.	Inflammation (general)	GER190
Iresine diffusa H.B.K. ex Willd.	Paja Blanca, Sangrinaria	Whole plant, fresh	Oral	Boil 10g per 1l water, mix with Ambarina, Lancetilla, Hierba de la Rabia, and Palo de Sange. Drink three times per day or as needed, 1l daily, for 1 year.	Liver, Kidneys, Inflammation of the ovaries, Blood, Menstruation symptoms in adolescents	JULS75, ISA62
Iresine herbstii Lindley	Colores, Timoras, Zangurache	Leaves, fresh	1. Topical<br>2. Oral	1. Fresh Leaves only, may use with Yonque, 7 Espiritus, and Bully Vinegar and Contrahierba. As poultice, 3 times a week.<br>2. Boil 5g per 1l water with Lancetilla, Contrahierba, Cachorillo, or chop, eat fresh. Oral once a day for a week or drink 1l daily, 1 month, always before breakfast.	1., 2. Liver, Kidneys, Cancer of the blood, Blood circulation, Intoxication of the blood, Heart, Nervous system, Blood, Inflammation of the stomach, Inflammation	ISA42, EHCHL114, ISA121
ALSTROEMERIACEAE						
Bomarea angustifolia Benth.	Cachuljillo	Whole plant, dried	Oral	1 Tbsp per 1 cup boiling water, 1 cup daily, 8 months.	Infertility in women	ISA27
Bomarea dulcis (Hook.) Beauv.	Espuela de Gallo	Whole plant, fresh	Seguro	3 Stems per flask.	Protection, Success, Advising	ISA95

Family/Genus/Species	Indigenous name	Plant part used	Admin.	Preparation	Use	Coll. #
AMARYLLIDACEAE						
Eustephia coccinea Cav.	Tumapara, Pomanpara, Puma Para, Para Para	1., 4. Bark, fresh or dried<br>2., 3. Bark, dried	1., 2., 4. Oral<br>3. Topical	1. Macerate in a bottle of wine, 3 small glasses per day.<br>2. Boil 200g of Pomanpara in 1l of water for 10 minutes. Drink cold, 1 cup every 3 days for 1 month.<br>3. Crush and pulverize with a rock, then drain to create a powder. Place powder on top of the wound. 1 time per day until the wound is healed. Boil for 20 minutes, 20g of herb per one liter of water mixed with Matico Malva, and Talla, wash wound one time per day for eight days.<br>4. Boil 3-5minutes, 5-10g per 1l of water mixed with Flor Blanca Purenrosa, Malva Olorosa, drink 3 times per day for 8 days.	1. Arthritis, Rheumatism<br>2. Inflammation of the stomach, Untangle witchcraft<br>3. Wounds<br>4. Inflammation, Hemorrhages, Inflammation of uterus, Ulcers, Cysts, Cancerous wounds	RBU/PL313, GER71, EHCHL68
ANNACARDIACEAE						
Annacardium occcidentale L.	Marañon	Seeds, fresh	Topical	Cut Seeds and extract/collect "blood". Topical application in affected area.	Scars, Moles, Cysts (ingrowing), Skin stains	GER48
Loxopterygium huasango Spruce ex Engl.	Hualtaco	Wood, dried	Oral	Boil 20g of Hualtaco with Diego Lope, Suelda con Suelda, and 1l of water for 30 minutes. Patient should drink cold solution. Do not exceed 3 dosages of the treatment because it is very strong. 1/2 cup a day every other day or as needed.	Bone or muscular pain due to an accident, Pain from bone fractures	GER109
Mangifera indica L.	Mango	Leaves, dried	Oral	Boil 5 Mango Leaves with 10 Moy Leaves, 10 Eucalyptus Leaves, 5 Stems buds of Pajaro Bobo and 1 Limon (all dried Leaves) in 1l of water for 30 minutes. Drink cold, 2 tablespoons 2 a day for 3 days.	Bronchitis, Colds, Inflammation (chest)	GER49
Mauria heterophylla H.B.K.	Shimir, Tres Hojas, Trinidad, Chacur, Ahimir, Feregreco	Leaves, fresh	1., 3. Topical<br>2. Oral	1. Boil 50g with Lailambo, Nogal, Ajenco, Timolina. Limpia, once a week.<br>2. 10g per cup, combined with Cola de Caballo, Verbena, Amor Seco, and various others. Drink the solution and use as a wash. Take 4 cups per day, 1 month.<br>3. 1/2l per 1 Stems with Leaves, not mixed with others. Situate legs in a ""V"" position and drop solution into vagina for 10 minutes, go to the restroom and contract muscles till fluid has drained. Repeat if necesary. Twice a month.	1. Daño, Fright / Susto, Skin irritation from daño<br>2. Inflammation, Liver, Kidneys, Wounds, Inflammation of uterus, Cleansing (external), Cleansing (internal), Ulcers (internal), Ulcers (external), Inflammation of the ovaries, Cysts, Fibroids<br>3. Vaginal cleansing	ISA24, JULS17, EHCHL83

Family/Genus/Species	Indigenous name	Plant part used	Admin.	Preparation	Use	Coll. #
Schinus molle L.	Molle, Moy	1., 2. Flowers, Leaves and Stems, fresh<br>3., 4. Bark and Latex, fresh	1., 4. Topical<br>2., 3. Oral	1. Macerate material in alcohol and spray on patient at nighttime. Once daily for five days as poultice or rub the patient's body with plant material while bathing in the mixture. Advise the patient to rest and to avoid going outdoors.<br>2. 20g, crush and mix with alcohol, boil 20-30 hours, mixed with Eucalyptus, Ruda, Chamana, Tilo. Take 1 cup, 4 times a day for 2 months or as needed.<br>3. Add 20g of Bark Resin (approximately 5cm) in 1l of water. Boil the combination for 3 minutes. Take 1 cup, 4 times a day for 2 months or as needed.<br>4. Add 20g of Bark Resin (approximately 5cm) in 1l of water. Boil the combination for 3 minutes. Use 1 cup 4 times per day for 2 months as vaginal douche.	1., 2. Arthritis, Rheumatism, Bone pain, Bronchitis, Cough, Cold, Chills, Inflammation of the body<br>3. Cancer, Tuberculosis<br>4. Vaginal infection	EHCHL123, JULS196, GER13
ANNONACEAE						
Annona muricata L.	Huanabana, Graviola	Leaves, fresh	Oral	Boil 1/2l of water with 10 Leaves of Huanabana and 10g of Amor Seco, Peel of Pinapple, Achote for 3-4 minutes. Drink cold, 3-4 cups a day for 1 month.	Gastritis, Inflammation, Kidneys, Cancer	GER2, EHCHL81
APIACEAE						
Ammi visnaga (L.) Lam.	Bisnaga	Flowers and Leaves, fresh	Topical	20g crushed Leaves as poultice, or 20g per 5l water for 20 minutes as bath, 3 times per week.	Bad Air / Mal Aire, Headache	EHCHL134
Apium graveolens L.	Apio Cimarron, Apio	Whole plant, fresh	1. Oral<br>2. Topical	1. Boil 1l water, then add 10g Apio Cimarron. Combine with Manzanilla, Mejorana, and Culantrillo. Drtink 4 cups per day for 1 week.<br>2. Boil with Perejil. Mix with Agua del Susto, 7 Espiritus. 3 baths per month.	1. Colic, Bronchitis, Heart, Nerves, Insomnia, Anxiety, Gases, Gastritis, Colic of the stomach<br>2. Freight in children / Susto en niños, Gastritis	JULS21, ISA79, ISA116, EHCHL106
Arracacia xanthorrhiza Bancroft	Racacha, Racacha Cimarrona	Leaves and Stems, fresh	Topical	Boil 20g of plant material with Flor de Chocho, Eucalyptus, Chueguis, and 2l of water. Bathe the patient in the warm mixture while rubbing him with the Leaves. Bathe 3 times a week for 1 month using 1 cup of material.	Fright / Susto	JULS278
Coriandrum sativum L.	Culantro	Leaves, fresh	Topical	Place fresh Leaves on top of the eyes. Apply only once. Leave it for 1 hour.	Mal Aire that takes your sight	GER47
Daucus montanus H. & B. ex Spreng.	Zanahoria de Zorro, Zanahoria de Gentil, Zanahoria	1. Leaves and Stems, fresh<br>2., 3. Root, fresh	1., 2. Topical<br>3. Oral	1. Leaves with Bully Vinegar and 7 Espíritus, 1 bundle with 3l boiled water with Ishpinguillo, Conchalalay Blanco, Manzanilla de Cerro, Lailambo, and Timolina. As bath, limpia or poultice, 1-2 times per week.<br>2. Grate root, 3 drops in each eye, or on affecteed areas of skin, 2 times a day for 2 to 3 days.<br>3. 50g of the tuber and 1/4 cup of water, blend and strain.1 glass 1 time a day for 15 days. Drink cold during breakfast time while fasting.	1. Bad Air / Mal Aire, Sorcery, Twists caused by sorcery, Twists, Contusions caused by Ssrcery, Susto in the Huacas / Susto en las huacas, Sustos of the summits / Susto de las cumbres<br>2. Eye sight, Sun spots<br>3. Inflammation	ISA33, ISA38, ISA68, JULS271, GER172

Family/Genus/Species	Indigenous name	Plant part used	Admin.	Preparation	Use	Coll. #
Foeniculum vulgare P. Miller	Hinojo, Anis Criollo	1. Whole plant, fresh<br>2. Seeds, fresh	Oral	1. Boil 5g per 1l boiling water, combine with Manzanilla, Poleo, Toronjil, Pimpinela, Clavel and Borraja, drink 3 times per day, 1 month.<br>2. Add 1 teaspoon of plant material to 1 cup of water. Boil mixture for 2 minutes. Drink warm. Honey or sugar can be added, if desired, twice a day, for 2 days.	1., 2. Colic, Gases, After birth, Stomach pain, Stomachache, Nerves, Diarrhea	EHCHL23, JULS101, JULS166
Hydrocotyle bonariensis Commerson ex Lam.	Tutapure de Estrella	Leaves and Stems, dried	Topical	1 handful boiled with 3l water. Can combine with Bejuco Amarillo and Palo Blanco. 1 bath per month.	Sorcery	ISA8
Hydrocotyle globiflora R. & P.	Sombrerito	Whole plant, fresh	Oral	4 small Leaves, 2 Flowers per 1l, boil 3 min. Drink 3 times per day, for 1 month.	Liver, Inflammation of the kidneys	JULS63
Niphogeton dissecta (Benth.) Macbr.	Hornamo Toro, Orma Motora	1. Leaves and Stems, dried<br>2. Whole plant, fresh	1. Topical<br>2. Oral	1. Boil 20g per 5l, 20 min. Mix with other Hornamos. Bath, 3 times per week.<br>2. Boil 5g per 1/2l, drink 1 cup per day, 1 month.	1. Wounds (cancerous), Wounds from sorcery<br>2. Purgative	EHCHL166, RBU/PL338
Petroselinum crispum (Miller) A.W. Hill	Perejil	Whole plant, fresh	1. Oral<br>2., 3. Topical	1. 3-5g of herb per 1l of water, combined with Toronjil, Pimpinela, Mejorana, Siempre Viva. Take 1 glass 2 times a day, 3 days before the menstrual period and three days after. Can also be eaten as salad.<br>2. Bundle fresh Leaves and Stems with Apio and burn.<br>3. Crush herb and boil with meat and salt. Apply as poultice.d other herbs. 2 per month as limpia or bath.	1. Heart, Nervous system, High blood pressure, Infections, Nose bleeds, Food condiment, Forgetting love or trauma, Regulation of menstrual cycle<br>2. Daño, Fright / Susto<br>3. Infections, Nose bleeds, Food condiment, Forgetting love or trauma	ISA80, EHCHL31, ISA117, RBU/PL278, JULS225
Pimpinella anisum L.	Anis Criollo, Anís	Seeds, dried	Oral	Tea, 5-20g per 1l boiling water, with Menta and Manzanilla, 2-3 cups a day for 3 days or as needed.	Gases, Stomach Pain, Colic	EHCHL137, TRUBH21, GER213
APOCYNACEAE						
Mandevilla antennacea (A.DC.) Schum.	Bejuco Colambo Negro	Whole plant, fresh	Charm	Plant and grow close to the house.	Protecting the house and field	GER236
Mandevilla cf. *trianae* Woodson	Bejuco, Bejuco Negro (Grande), Bejuco Negro (Chico)	Leaves, fresh or dried	Topical	1 handful of per 3l boiled water. Can combine with Zanahoria, Gentil, Chilca, Añasquero Chico, Ishpinguillo, Conchalalay, Hierba del Susto (if it's used for susto), 7 Espiritus. Bathe once a week and have a limpia once a month.	Daño de Brevaje, Fright / Susto, Sorcery	ISA14, ISA13
Nerium oleander L.	Laurel, Laurel Rosa	Flowers, Leaves and Stems, fresh	Topical	Boil 30g Laurel with 5l water for 5 minutes. Use water to shower. Rub Leaves on the skin. Don't touch the eyes or mouth while washing because the Leaves are poisonous. Wash every other day for 7 days or 3 times: Tuesday - Friday - Tuesday.	Cleaning wounds, Itching, Sarna, Rashes, Skin mark, Herpes	JULS65, GER45

Family/Genus/Species	Indigenous name	Plant part used	Admin.	Preparation	Use	Coll. #
Thevetia peruviana (Pers.) Schum.	Mailchin, Maichil, Camalonga, Cabalonga	1. Stems and Leaves, fresh 2., 3. Seeds, dried	1. Topical 2., 3. Oral	1. Bath, add 10g of plant Leaves with 2l of water and boil the mixture for 3 minutes, or boil 20 min in 5l water, apply the mixture lukewarm. Rub the patient with the Flowers and the water. Right after bath, advise patient to dress in warm clothing. 2 baths per week (Tuesday and Friday) or 3-4 times a month. Do not ingest! 2. Ground and boiled, best macerated in wine that has a small amount of alcohol, must use holy wine from the church. Mixed with Seeds from a specific seven other plants: Ashango, Pucho, Amala, Quina Quina, Mozcada, Ishpingo, 1 time per month or as needed. 3. Put together 1 Seeds of Cabalonga in 1 bottle of wine and let it sit for 8 days, one small wine glass once a day for 20 days or as needed.	1. Bones, Rheumatism, Bad Air / Mal Aire, Arthritis, Cause bad luck / Cast a spell (Sorcery) 2. Bad Air / Mal Aire, Epilepsy, Nerves, Heart attacks 3. Menopause, Cancer, Bad Air / Mal Aire, Sorcery	EHCHL162, TRUVan/Erica19, JULS187, EHCHL174, GER225
Vallesia glabra (Cav.) Link.	Cuncuno, Cun Cun	Leaves, fresh	Oral	1. Boil 15 Leaves of Cuncuno with 10 Seeds of Fuque and 1/8L of cooking oil into 1l of water for 20 minutes. Drink cold, 1/2 cup 2 a day (6 AM and 6 PM) for 2 days. Do not eat fish or spices (no chiles) and stay away from the sunlight during treatment. 2. Blend 15 Leaves until you have an extract. Patient should drink cold solution. Drink only at 6 AM. Do not eat anything sweet while on treatment. 1 small glass every morning for 30 days.	1. Snake bites 2. Diabetes	GER26
AQUIFOLIACEAE						
Ilex guayusa Loes	Guayusa, Agracejo, Citrodora	Leaves, dried	Oral	Tea, 5-10g per 1l water, 1 cup three times per day as needed, drink warm.	Diabetes, Intoxication of the blood	EHCHL130, JULS160
ARALIACEAE						
Oreopanax eriocephalus Harms	Maque Maque, Mano de León	Leaves and Flowers, fresh	1. Oral 2., 3. Topical	1., 2. Boil 3-10g per 1l of water. Drink 3-4 times per day for 1 month or take as bath. 3. 5g per 3l boiled water, mixed with Laurel, Rumilanche, Poleo de Gentil, 7 Espiritus, and Manzanilla Blanca, Romero Castillo, Manzanilla Blanca, Nogal. Bathe 3 times a month.	1., 2. Heart, Nerves, Inflammation, Fractures, Nervous system, Rheumatism, Protection from evil 3. Fright / Susto	EHCHL36, JULS39, RBU/PL270, ISA71, GER221
ARAUCARIACEAE						
Araucaria heterophylla (Salisb.) Franco	Pino	Resin, fresh or dried	Topical	Warm up a small piece of Resin. Place Resin on top of the tooth affected. 2 times a day as needed or until tooth is healed.	Toothache, Extracting teeth	JULS229
ARECACEAE						
Bactris spp.	Chonta	Wood	Charm	Pass stick over body while praying.	Protection	GER229

Family/Genus/Species	Indigenous name	Plant part used	Admin.	Preparation	Use	Coll. #
Cocos nucifera L.	Coco	Peel of the Fruit, dried	Oral	Grind 10 Seeds of Coco. Boil in 1/4l of water for 20 minutes combined with 1-2 Leaves of Hierba Luisa, Culen, Hinojo, and Poleo. Boil the mixture for 3-5 minutes. Drink 3 times a day, for 2-3 days. Drink the coconut milk for inflammations.	Diarrhea, Parasites, Inflammation of the liver, Inflammation (general)	JULS145
ARISTOLOCHIACEAE						
Aristolochia ruiziana (Klotzsch) Zahlbr.	Bejuca del Contraaire	Stems, dried	Oral	Boil 20g of Bejuca with 1 cup of water for 10 minutes. Patient should drink cold solution. Once only.	Untangling a person who is having trouble progressing in life, Untangling a person who is a mess	GER107
ASCLEPIADACEAE						
Sarcostemma clausum (Jacquin) Schultes	Marrajudio	Leaves, Stems, fresh	1. Topical<br>2. Oral	1. 2l of water, add 10g of Flor de Retama, Quinual, Flor de Chuco, 20g of Eucalyptus. Boil for 3 minutes. Let it cool. Once cooled proceed with taking a bath. 2 to 3 times a week as needed. Alternatively break a Stem and collect Resin. Apply on affected area. Twice a day (AM and PM) as needed.<br>2. Boil 5 Leaves and 1 Stems of a plant in 1/2l of water for 10 minutes. Drink cold, 1 small cup 3 times a week, AM only.	1. Fright / Susto, Rashes, Pimples, Cold sores, Skin marks<br>2. Promoting lactation in women after birth	JULS121, GER43
ASPHODELACEAE						
Aloe vera (L.) Burm f.	Sabila, Zabila, Aloe, Hojas de Sabila, Aloe Vera	Leaves, fresh	1. Topcial<br>2. Oral<br>3. Charm	1. Take a wide leaf. Take the spines out and cut the leaf longitudinally open. Get rid of the iodine secretion. Apply over inflamated area (hot) as poultice. 2 times a day for a week. For vaginal inflammation insert leaf, 2-3 deposits a day or as needed. One deposit at a time, leave it inside for 5 minutes take out that one and insert the next. In case of Hair loss apply to hair and skin. Juice can also be applied to eyes: Apply one drop on each eye. 1 drop every 2 days for 6 days.<br>2.1kg of herb, 1/2kg of Honey, and three Tbsp of Pisco. Open the leaf longitudinally and exctract the iodine secretion and the internal gel from the inside of the leaf. Consume the iodine secretion and the gel. 1-2 cups per day for a week to a month. Leaf can also be macerated in a bottle of alcohol.<br>3. Hang whole plant in doorway.	1. Inflammation (external), Vaginal inflammation, Vaginal ulcers, Vaginal cancer, Hair growth, Skin embelishment, Cataracts, Eyes, Wounds, Burns<br>2. Weight loss, Gastritis, Inflammation, Diabetes, Cough, Bronchitis, Kidneys, Ulcers, Cholesterol, Cancer, Asthma, Bile<br>3. Good Luck, Happiness	JULS274, GER22, EHCHL165, VFCHL10
ASTERACEAE						
Acanthoxanthium spinosum (L.) Furreau	Juan Alonso, Espina de Perro, Corona de Cristo	Whole plant, fresh or dried	Oral	Boil 10g of plant material with 1l of water for 3-5 minutes. Also add Alcaparria and Guava Seeds. Drink warm, 1-2l per day, for 2-3 months.	Detoxification of alcohol and drugs, Inflammation, Bronchitis, Haemorrhages	JULS176, EHCHL32

Family/Genus/Species	Indigenous name	Plant part used	Admin.	Preparation	Use	Coll. #
Achillea millefolium L.	Milenrama, Chonchon	Flowers and Leaves, fresh	1. Oral, 2. Topical	1. Boil 3-5g per 1l of water, drink 3 times per day for one week.<br>2. 1 bundle per 5l boiling water, 3 baths per month during the night.	1. Gastritis, Diabetes, Blood, Cholesterol<br>2. Skin infection, Dispel bad spells	RBU/PL371, RBU/PL361, EHCHL56
Achyrocline alata (H.B.K.) DC.	Ishpinguillo, Ishpingo, Flor de Ishpingo	1., 2. Stems and Leaves, dried	1., 2. Topical	1. Bath and limpia, 5g boiled with 3l water, mixed with Añasquero Grande, Ajenco, Tres Hojas. 2 times per month. Alternatively 1/2kg, boiled 10 minutes for a steam bath.<br>2. Poultice. Use 1 bundle fresh Leaves with 7 Espiritus and Bully Vinegar. 6 hours duration, 2 per month as long as illness requires.	1. Fright / Susto, Daño in children, Arthritis, Bones<br>2. Arthritis, Bones	ISA85, ISA109
Acmella cf. *ciliata* (H.B.K.) Cas.	Ufla	Root, dried	Oral	Boil 100g of Ufla root and 100g of Menta in 1l of water for 10 minutes. Patient should drink lukewarm solution. 2 times a day for 3 days.	Internal bleeding, Renal bleeding, Cold with high mucus	GER7
Ambrosia arborescens Miller	Ambrosia	Whole plant, fresh	1. Topical<br>2. Seguro	1. Alternative mixture for Spiritual Flowering, see below. One time only.<br>2. Standard Seguro mixture, see below.	1. Spiritual Flowering<br>2. Good business, Protection, Good fortune, Good health	GER118
Ambrosia peruviana Willd.	Altamisa, Marco, Artamisa, Manzanilla del Muerto, Marcos, Alta Misa, Ajenjo, Altamis, Llatama Negra Malera, Llatama Roja Malera	Leaves and Stems, fresh	1. Oral<br>2., 3. Topical	1. Boil 1l water 2 min, then mix water with a total of 10g of Manzanilla, Borraja, Madre Selva, Toronjil, Hinojo and Chancas de Comida for nerve disorders. Use Boldo, Malva, and Linaza for liver ailments. Use Matico, Borraja, Eucalipto, Vira Vira, and Brochamelia for Bronchitis. Cover and let sit for 2-3 minutes. Drink lukewarm, 3-4 cups a day for a month. Colds: Boil 1/2l of water with 50g of Altamiz and 10g of Sauce, Chicoria, and Pajaro Bobo for 10minutes. 2 tablespoons every 8 hours for 8 days.<br>2. Boil 200g with 3l water and 7 Espiritus, Agua de Susto. Use as poultice, or bath, 6 hrs per bath 2 times per month, 1 month. For Susto 3 times a week: Tuesday, Friday, and the following Tuesday.<br>3. Emplasto, crush 200g of leaf and add 5 drops of Trementina. Place emplasto on affected area (the woman's belly) and cover with a piece of cloth. Leave for 2 hours. 2 times only every 2 days.	1. Heart, Nerves, Epilepsy, Liver, Bronchitis, Colds, Bad Air / Mal Aire, Burns<br>2. Fungus, Fright / Susto<br>3. After birth to reduce inflamation and prevent spasms in the woman's womb	JULS108, TRUBH18, RBU/PL370, TRUBH15, JULS90, GER9, GER110
Arctium lappa L.	Lampazo	Seeds, dried	Oral	Boil for 5 minutes 1/2l of water with 10g of Cadillo, Amor Seco, Triñozo. Drink lukewarm. 1-2 cups 3 times a day for 20 days as needed.	Urinary problems, Skin, Liver, Gallbladder, Intestine, Tumors	GER227
Arnica montana L.	Arnica	Commercial tincture	Charm	Place close to patient, inhale. Splash all corners of the house in the center of the house and make a sign of the cross at the front door with it.	Waking a person who has fainted, Keeping bad spirits away from the house	JULS106

Family/Genus/Species	Indigenous name	Plant part used	Admin.	Preparation	Use	Coll. #
Artemisia absinthium L.	Ajenco	1., 2. , 3. Leaves, fresh<br>4. Whole plant, preferably Leaves and Stems, fresh	1., 2., 3. Topical<br>4. Oral	1. Natural, as limpia, 1 bundle with Bully Vinegar, Añasquero grande, Añasquero chico, Flores de Muerto, Hierba del Susto, 7 Espiritus, Agua del Susto. Twice per week (Tuesday and Friday).<br>2. Bath, with Añasquero Grande, Ruda Hembra, 7 Espiritus, and Agua del Susto (if you have Susto). twice per week.<br>4. Tea, 6-10 Leaves per 1 cup boiling water. 1 cup daily for 3 days OR add 100g of the plant sample with 1 cup of water. Boil the mixture for 5 minutes, take 1/4 cup, once a day, for 3 days.	1., 2. Fright / Susto in children, Sorcery<br>3. Sorcery<br>4. Menstrual colics, Menstration, Regulating the menstrual cycle	ISA66, RBU/PL363, GER146
Baccharis caespitosa (R. & P.) Pers. var. *alpina* (H.B.K.) Cuatr.	Paja Amargoza,	Flowers and Leaves, fresh	Topical	Grind 500g material, apply as poultice. 1 per day for 8 days. put on affected area.	Swellings	GER260
Baccharis ciliaris (Retz.) Koeler	Pata de Gallina	Whole plant, fresh or dried	Seguro	Mix in a bottle 10g of Valeriana Estrella, Señorita, Carpintero, Chupa Flor, Hierba la Coqueta, Oro, Dollar. Add Agua Florida, Ramillete de Novia, Tabu, Lima juice, Agua Bendita, sugar. One bottle to keep forever.	Protection of job and house, Protection (general)	JULS220
Baccharis genistelloides (Lam.) Pers.	Simba Simba, Carceja, Karqueja, Cadillo	Whole plant, fresh	Oral	10g per 1l water, boil for 2 minutes only. Mixed with Canchalagua, Verbena, Amor Seco, Cola de Caballo, Hierba del Toro, Campote, Mal Rubio. Take at breakfast and dinner, 1l per day, for one week to one month.	Diabetes, Blood, Burn fat, Cholesterol, Kidneys, Internal Inflammation, Liver, Gallbladder, Bad blood, Baldness	EHCHL101, TRUBH10, RBU/PL255, JULS34, VFCHL36, EHCHL92
Baccharis glutinosa Persoon	Chilco Macho	Leaves, fresh	Oral	Boil 1l of water and 100g of plant material. Drink mixture 3 times a day, for 1 month.	Diabetes	JULS135, GER29, GER198
Baccharis inidica (L.) Gaert	Pata de Gallina	Whole plant, fresh or dried	Seguro	Mix in a bottle 10g of Valeriana Estrella, Señorita, Carpintero, Chupa Flor, Hierba la Coqueta, Oro, Dollar. Add Agua Florida, Ramillete de Novia, Tabu, Lima juice, Agua Bendita, sugar. One bottle to keep forever.	Protection of job and house, Protection (general)	JULS220
Baccharis latifolia (R. & P.) Pers.	Chilca Chica, Chilca Grande	1. Leaves and Stems, fresh or dried<br>2. Leaves, fresh	1., 2. Topical	1. Bath, 5g per 3l water mixed with Manzanilla Blanca, Hierba del Susto, Laurel, and Agua del Susto, 2-3 baths per 1 month, for limpia 1 per week.<br>2. Poultice, 200g fresh Leaves mixed with 7 Espiritus. 1 bundle of fresh Leaves per 5-6 hours with a bandage to keep the leaf in place. Apply for 6 hours, 2-3 times per month.	1., 2. Hot bones, Bone pain, Rheumatism, Arthritis	ISA86, ISA115
Baccharis odorata H.B.K.	Pasto Miel	Whole plant, dried	Topical	Heat with Agua Florida. Place heated emplasto on affected area with a cloth, leave for 2 days as it will absorb the cyst. As needed.	Cysts, Wounds (clotted), Abcesses	JULS219

Family/Genus/Species	Indigenous name	Plant part used	Admin.	Preparation	Use	Coll. #
Baccharis salicifolia (R. & P.) Pers.	Hierba de la Plata, Chilco Hembra	Whole plant, fresh	1., 3. Topical 2. Seguro	1. Alternative mixture for Spiritual Flowering, see below. Bathe once. 2. Standard Seguro mixture, see below.	1., 2. Good business, Protection, Good fortune, Good health 3. Allergies, Rashes, Pimples	TRUVan/Erica5, GER125, GER84
Baccharis vaccinioides H.B.K.	Sigueme Sigueme	Flowers and Leaves, fresh	Topical	1. Standard Seguro mixture, see below. Some spiritual prayer invoking the name of the patient, owner of the seguro. Fogear on Tuesdays and Fridays. Spray and rub the mixture on the patient for good luck. 2. Bath, 50g of all: Hierba del Lucero, Hierba del Este, Ambrocilla, Senorita, Caballero, Pega Pega, Siempre Viva, Carpintero, Waime Waime, Piri Piri (Hembra y Macho), Hierba del Buen Querer, Hierba del Oro, Hierba de la Plata, Hierba del Halago, Sigueme Sigueme, Hierba del Negocio boil into 5-7L water and boil for 20 minutes then add a bit of the following perfumes: Cariño, Dios de la Huaringa, Dios de la Felicidad, San Antonio, Macumba Pusanga, Gran Jefe, Mil Flores, Llama Plata, and Ekeko and let it cool before bathing. 2 times (Tuesdays and Fridays only) every 3 months.	Good luck, Spiritual Flowering / Florecimiento	GER188, TRU BH 28
Bidens pilosa L.	Amor Seco, Cadillo, Morseco, Tres Esquinas, Karqueja	Whole plant, fresh or dried	1. Oral 2. Topical	1. 10g per 1l of water, combined with Chacur, Unquia, Flor de Arena, Espiga de Maiz, Cola de Caballo, Guanabana, Pimpinela and las Flores de Azares. Drink 1 cup four times per day for one month. 2. Same mixture can be used as bath.	1., 2. Gallbladder, Kidney Inflammation, Inflammation (general) , Kidneys, Prostate, Hair loss, Diabetes, Liver, Blood, Heart	JULS74, VFCHL25, EHCHL18, ISA127, GER1
Chuquiraga spinosa sp. *huamanpinta* C. Ezcurra	Chuquiragua, Huamanpinta	Leaves, dried	Oral	Boil 5-10g per 1l water for 3-5 min. 1-4 cups per day, 15 days or as needed. For impotence macerate 6 plants for 8 days in wine in a 1l bottle. Mix with eggs of Angelote, Pollen, Catachi, and Viril de Oso. Take as needed.	Inflammation, Kidneys, Prostate, Bladder, Prostate inflammation, Sexual impotence	EHCHL168, TRUBH9, JULS276, RBU/PL373
Chuquiragua weberbaueri Tovar	Amaro Amaro	Whole plant, fresh or dried	Oral	Boil 10g in 1 L of water for 3-4 minutes with Eucalyptus, Matico, Mullaca, Muña, Flor de Overo. Take one cup 3-4 times a day for a month.	Cough, Bronchitis, Asthma, Liver, Air	JULS99, EHCHL131
Clibadium cf. *sylvestre* (Aubl.) Baill.	Flor de Novia	Flowers, Leaves and Stems, fresh or dried	Topical	1 bundle, 20 drops of perfume per 3l boiling water. 3 baths per month.	Cold, Before marriage	EHCHL80
Cronquistianthus lavandulifolius DC.	Clavelillo, Espino de Hoja, Pulmonaria	Flowers, Leaves and Stems, fresh or dried	Oral	Add 10g of plant material, Matico, Zarzamora, Nogal, Salvia, Borraja, Llatama, Vira Vira. with 1l of water. Boil the mixture for 3-4 minutes. Drink 1l daily, 3 months.	Cough, Bronchitis, Headache, Cold, Asthma, Pulmonary disease	ISA5, JULS233, GER163

Family/Genus/Species	Indigenous name	Plant part used	Admin.	Preparation	Use	Coll. #
Cynara cardunculus L.	Alcachofa	Stems and Leaves, fresh or dried	1., 2. Oral	1. Boil 10g per 1l water, 5 min, 1l per day or 3-4 four glasses per day.<br>2. Add 1l water with 1 Alcachofa and boil for 5 minutes, Take 1 cup, three times a day, for 1 month.	1. Diabetes, Memory, Physical weariness, Liver, Blood purification, Mental weariness<br>2. Weight loss	VFCHL31, RBU/PL261, JULS94
Diplostephium gynoxyoides Cuatr.	Parrano	Flowers, fresh	1. Oral<br>2. Topical	1. Boil 10 Flowers of Parrano and 4 Leaves of Chicoria in 1/2 cup of water for 2 minutes. Patient should drink hot solution. 3 tablespoons 3 times a day for 5 days.<br>2. Crush 2kg of Parrano Leaves + 200g of Flor de Muerto, Frejol Chileno, Garlic, and Agua Cananga (perfume). Place on affected area and cover with a piece of cloth for 3 hours.	1. Cold, Inflammation of the lungs<br>2. Mal de Aire	GER5
Diplostephium sagasteguii Cuatrecasas	Hierba del Tigre, Gato Simura	Leaves and Stems, fresh or dried	1. Oral<br>2. Topical<br>3. Seguro	1. Boil 10g of Hierba del Tigre, 10g of Hierba del Oso, and 10g of Semora Negra, 3 Leaves of Toro Simuro, and 3 Leaves of Mishia Amarilla in 1/2 cup of water for 5 minutes. Very strong compound. Do not exceed the dosage. 1/8 cup 1 time only. Drink cold. Patient should stay inside the house without any light or noise for 3 days. Should also observe a diet (no spices or seafood).<br>2. Bath mixture for Protection from Evil, see below. Only once.<br>3. 2 small branches per seguro.	1., 3. Bad Air / Mal Aire, Fragrance, Good luck for work, Daño (prevention), Undo bad things done to you, Strength, Maldad (cure), Evil eye /Mal ojo<br>2. Protection from evil	GER61, RBU/PL336, ISA139, TRUBH24, GER58
Eupatorium gayanum Wedd.	Asma Chilca, Asma (Chica)	Leaves, fresh	1. Topcial<br>2. Oral	1. 200g with Balsamo de Buddha. Use as poultice, 2 times per month.<br>2. 5g per 1l. mix with Tilo, Huamanripa, Borraja, Nogal. 4 cups per day, 10 days.	1., 2. Cough, Bronchitis, Asthma	RBU/PL276, EHCHL164
Eupatorium triplinerve Wedd.	Chilco Hembra	Leaves and Stems, fresh	Topical	Boil 5l of water with 20g of plant material and Laurel for 5 minutes (similar to a tizana). Bathe 2 times a day, for 1 week.	Rashes	JULS134
Ferryanthus verbascifolius (H.B.K.) H. Robinson & Brettell	Tutapure Amarillo,Tutapure Amarillo (Grande)	Whole plant, fresh	Topical	Mix with Timolina, 2 times per week as limpia. Mixed with Tutapure Negro, Manzanilla Blanca, Añasquero Chico, Ruda Chingue, Conchalay, Ticra, Manzanilla, 7 Espiritus as bath, 3 times per week. Can also be used as steam bath once a month.	Fright / Susto, Sorcery, Diarrhea in children from Susto, Daño	ISA36, ISA9, ISA11
Flaveria bidentis (L.) Kuntze	Mata Gusano	Flowers, Leaves and Stems, fresh or dried	Oral	Boil 1l water, then add 10g Mata Gusano. Drink 3-4 times per day for 1-2 weeks, or as needed.	Cough, Bronchitis	JULS68
Gnaphalium americanum Mill.	Lechuguilla	Whole plant, fresh or dried	Oral	Boil 1/2l of water with 10g of Lechugilla. Patient should drink lukewarm solution. 1 glass 2 to 3 times a day for 1 month.	Diabetes, Nerves	JULS179
Lactuca sativa L.	Lechuga	Root and Stems, fresh	Oral	Add 100g of the plant material, with 100g of Beterraga, 1/2l of water and heat. After heating, let mixture cool. Drink 1 cup, 2-3 times a day for 1 month.	Nerves, Blood purification, Clean toxins from blood	JULS178, GER179

Family/Genus/Species	Indigenous name	Plant part used	Admin.	Preparation	Use	Coll. #
Loricaria ferruginea (R. & P.) Wedd.	Palmerilla, Palmita, Pata de Gallina, Palmera, Trencilla, Palmilla, Patita de Gallo, Palmia Pina, Palmera Blan	Leaves and Stems, fresh	1. Topical<br>2. Seguro	1. Alternative mixture for Spiritual Flowering, see below. 3 times per day, 1 week, only once for florecimiento.<br>2. Standard Seguro mixture, see below.	1. Spiritual Flowering / Florecimiento, Menstrual delay, Blood circulation<br>2. Protection, Good health, Good fortune, Good business, Fragrance, Succes, Good travels, Becoming sociable, Good relations with others	GER108, RBU/PL339, TRUBH20, ISA136, TRU Van/Erica23, TRU Van/Erica10, ISA148(105a)
Loricaria pauciflora Cuatr.	Palmilla Ancha, Palmilla Verde, Palma Bendita	Whole plant, fresh	1. Seguro<br>2. Topical	1. 7 small plants per Seguro, combine with strong magical herbs.<br>2. Boiled 15-20 minutes, 10-20g per 12l water, bath/saumo 2-3 times per month.	1. Business<br>2. To cast away bad spirits	TRUVan/Erica18, EHCHL13
Matricaria frigidum (HBK) Kunth	Manzanilla	Whole plant, fresh or dried	1. Oral<br>2. Topical	1. Boil water first. Add 10g Manzanilla per cup. Three cups per day for one week.<br>2. Boil water first. Add 10g Manzanilla per cup. Do not mix with other herbs. Rub solution over the womb or inflamed area. As needed. For vaginal inflammation squat over the steam 2-3 times per day, every other day. Alternatively boil Manzanilla, then place inside a cloth. Twist cloth to get the water out of it. May also boil a Manzanilla tea bag. Place cloth with herbs, or tea bag, on affected area for 3-4 minutes or until the tea bag or cloth is cool. Heat again and repeat the process. 3-4 times a day for no more than 2 days.	1. Pain of love, Nerves, Insomnia, Inflammation of wounds, Colic, Stomach ache, Bronchitis<br>2. Inflammation, Colic, Inflammation of the vagina, Injuries, Wounds (open), Wounds (closed)	JULS22, EHCHL1, TRUBH7
Matricaria recutita L.	Manzanillon, Agua de la Banda, Manzanilla Blanca, Manzanilla Amarga, Manzanilla	Whole plant, fresh	1. Seguro<br>2., 4. Topical<br>3. Oral	1. Three Stems per flask.<br>2. Boil the whole plant for 3-5 minutes. Mix entire plant with Hierba del Susto, Ajenco, after combining with 2 Tsps Bully Vinegar. Pour bath the mix in a bathtub and sit in it, rub the patient, 2-4 times a month. Boil 1 dried bundle per 1l water for 5 min for washings (wounds).<br>3. Boil water. Add 10g of Manzanillon into 1 cup of hot water. Manzanilla, Toronjil, and Pimpinela may be added. Take 1 small cup 3 times a day, for 1 month. Drink lukewarm.<br>4. Chop fresh herb to get extract, mix with odorles vaseline. Wash with Llanten, then apply ointment as needed.	1. Good travels, To make people more sociable, Good relations with others<br>2. Fright / Susto, Infection of wounds, Vaginal cleansing<br>3. Blood purification, Menstrual colics<br>4. Infection of wounds	JULS192, RBU/PL306, ISA120, ISA76, GER145
Mikania leiostachya Benth.	Enredadera	Leaves, dried	Topical	1 handful with 3l boiled water, combine with Huaminga, Chilca, Hierba del Susto, and Agua del Susto. Take 2 baths per week.	Daño, Daño de Brevaje	ISA12

Family/Genus/Species	Indigenous name	Plant part used	Admin.	Preparation	Use	Coll. #
Monactis flaverioides H.B.K.	Hierba del Susto (Amarillo), Malva, Mocura, Hierba del Susto, Hierba Susto	Stems and Leaves, fresh	1. Topical<br>2. Oral	1. 7 Stems with their Leaves boiled with 3-5l water, combined with Agua del Susto, Ajenco, and Llatama for 20 minutes. Bath, 2-3 times per week, at 7,9, and 11PM.<br>2. Mix with Toronjil, Pimpinela, boiled for 3-5 minutes. 1l daily for 7 days.	1., 2. Bad Air / Mal Aire, Prostate, Fright / Susto, Vaginal cleansing, Business, Casting away bad luck, Freight in children / Susto en niños	EHCHL19, RBU/PL274, TRUVan/Erica7, ISA104, ISA72
Munnozia lyrata (A. Gray.) Rob. & Brett.	Canillahuanga	Whole plant, fresh or dried	Topical	Bath, 20g per 5l, boil 20 min, with Hierba del Susto, Añasquero, Cutiquero, Hierba del Ave, Ishpingo. 3 times per week.	Fright / Susto, Bad Air / Mal Aire	EHCHL138
Onoseris odorata (D. Don) Hooker & Arnott	Hierba de Reina	Whole plant, fresh or dried	Oral	10g per 1l boiling water, 3 cups per day.	Heart, Nerves	RBU/PL337
Oritrophium peruvianum (Lam.) Cuatrec.	Huamanripa, China Linda, Wiña Wiña, Vira Vira, Oronamo, Hierba del Sol, Maguanmarica, Hierba del Lucero	Whole plant, fresh or dried	1. Oral<br>2. Topcial<br>3. Seguro	1. Add 10g of plant material per 1l, boil 3 min. 3 cups per day, as needed. Drink lukewarm.<br>2. Standard mixture for Spiritual Flowering, see below.<br>3. Standard Seguro mixture, see below.	1. Asthma, Bronchitis, Pneumonia<br>2. Fragrance, Attract lovers, Spiritual Flowering<br>3. Illuminating your path and destiny, Good business, Protection, Good fortune, Good health	JULS58, EHCHL126, TRUBH29, TRUBH26, ISA96, TRUVan/Erica2, GER166
Paranephelius uniflorus Poepp. & Endl.	Pacha Rosa, Carapa de Chancho	Whole plant, fresh or dried	Oral	1. 5g per 1l water, mixed with Flor Blanca, Purenrosa, Flor de Arena, Manayupa, Sauco, Cola de Caballo, Pie de Perro, or other anti-inflammatory plants can be mixed in as well. Addition of these other plants is optional. Take 3-4 times a day, for one month.	Inflammation of the ovaries, Uterus, Inflammation (internal female parts), Stones, Inflammation	EHCHL133, JULS125
Perezia multiflora (H. & B.) Lessing	Corzonera, Escorcionera, Escorzonera	Whole plant, fresh or dried	Oral	Boil 1l water, then add 10g Escorcionera. Combine with Matico, Eucalyptus, Veronica, Vira Vira, Nogal, Huamanripa, Tilo and Zarzamora. 3 cups per day for 15 days. Patient should drink cold solution.	Nerves, Cough, Bronchitis, Asthma, Sharp pain in the body	RBU/PL323, JULS16, EHCHL52, GER160
Perezia pungens (H.B.K.) Cas.	Lengua de Vaca	Leaves, fresh	Topical	1 bundle of Leaves, makes a cream. Apply 2 times per week.	Infection of wounds, Prevents Peeling of skin after sunburn, Twisting or fractures from sorcery	ISA4
Picrosia longifolia D. Don	Achicoria, Chicoria	Whole plant, fresh	Oral	Boil 10-50g of Chicoria and Verbena, Canchalagua, Chochocon per 1l water, 1l daily, 15-30 days. Alternatively chop and extract juice of 200g fresh material, drink 1 glass daily, no longer than a week. Overdosing can harm vision.	Liver, Blood, Hepatitis, Gallbladder, Purification of the blood, Bronchitis, Pneumonia	EHCHL116, JULS6, GER21

Family/Genus/Species	Indigenous name	Plant part used	Admin.	Preparation	Use	Coll. #
Porophyllum ruderale (Jacq.) Cas.	Hierba Gallinazo, Hierba del Gallinazo	Whole plant, dried	1. Incense<br>2. Topical	1. Burn with Llatama, Ajos Giro, Añasquero Chico, 5g each herb. 2 times per month.<br>2. 5g Llantén, Ajos Giros, Hierba Gallinazo, Hierba del Romero, Flor del Huerto, Eucalyptus, add Floripondio Flowers, Retama and Añasquero Chico per 3l. Bathe 2 times per month, Tuesday and Friday only. Rub body with herbs. Rinse with the water. Do not dried with a towel. Air dry.	1. Clean the energy of the home<br>2. Daño, Fright / Susto	ISA73, GER89, JULS180
Pseudogynoxis cordifolia (Cass.) Cabr.	San Juan	Whole plant, fresh or dried	Topical	Add 10g of plant material with 2l of water and 10g of Eucalyptus, Chancas de Muerto, and Romero. Boil the mixture for 3 minutes. Wash the patient in the lukewarm mixture during the evenings. Advise the patient not to leave the house afterwards. Bathe every 2 days.	Fright / Susto, Bad Air / Mal Aire	JULS294
Schkuhria pinnata (Lam.) Kuntze	Canchalagua, Canchalagua (Chica)	Whole plant, fresh	Oral	Boil 20g per 1l for 3-10 min. Mix with Ortiga, Lancetilla, Culantrillo, Panisara, Purenrosa, Boldo, Berro, of Flor Blanca and Canchalagua. Take 3 times per day (1l), 1 month. Blood purification: tizana must sit out over night before drinking.	Blood Cleansing, Liver, Blood purification, Gallbladder, Bad breath, Diabetes, Menstrual delay, Allergies, Menstruation, Blood irrigation, Inflammation of the urinary tract	RBU/PL266, JULS42, VFCHL27, GER228
Senecio canescens (H.B.K.) Cuatrecasas	Vira Vira, Oreja de Conejo	Whole plant, fresh	1. Oral<br>2. Topical	1. 10g diced herb in boiling water, combine with Borraja, Eucalyptus, Corzonera, Borraja, Cerraja, Polen de Hierbas, Manzanilla, Toronjil, Congona, Poleo, Claveles, Juan Alonso, Espina de Hoja, and Alcanfor. Drink 3 cups per day, 1 month.<br>2. Use same mixture for steam baths and inhalation.	Bronchitis, Asthma, Cough, Nerves	TRUBH8, RBU/PL322, EHCHL104, EHCHL24, ISA108, TRUVan/Erica12, JULS14, GER158
Senecio chinogeton Wedd.	Hornamo Leon Amarillo	Leaves and Stems, fresh or dried	1., 2. Oral	1. Boil at low temperature 3 pieces of 3 inches each of Hornamo Leon Amarillo and Hornamo Leon Verde, 1 slice of San Pedro of 6 lines and one of 7 lines and 3 pieces of 3 inches each of Condor Purga in 9L of water for 1 hour at low temperature. Drink cold, 1 small glass 1 time only.<br>2. Boil 2 leaves of Misha Morada, 1 leaf of Misha Amarilla, 1 leaf of Misha Blanca, 1 leaf of Misha Rosada, 1g of Toro Maigue and 1g of Toro Misha. in 1/2 cup of water for 5 minutes. Drink cold. Patient must stay in a dark room for 3 days while maintaining a diet without spices or seafood. Patient should rest for three more days afterwards. 1/8 of a small glass.	1., 2. Bad Air / Mal Aire, Inflammation (general), Hallucinoen / Vision enhancement	GER60

Family/Genus/Species	Indigenous name	Plant part used	Admin.	Preparation	Use	Coll. #
Senecio genisianus Cuatr.	Tutapure Blanco	Leaves and Stems, dried	Topical	1 handful per 3l boiled water. Can combine with Chuque, Huaminga, Chinque, Manzanilla de Cerro, with Bully Vinegar, 7 Espiritus. One bath per week, also for limpias.	Wounds (cleansing), Rabies, Animal bites	ISA16
Senecio hypsandinus Cuatr.	Ornamo Blanco	Whole plant, fresh	Seguro	A few Stems per flask.	Fragrance, Good Luck	RBU/PL358
Senecio pseudotites Grieseb.	Arnica	Leaves and Stems, fresh	Topical	In 1/2l water boil 100g of Arnica for 10 minutes. Wet a piece of cloth in the warm tizana, then squeeze a bit of the water out and place cloth on affected area for a few seconds. Repeat over and over again until body temperature is lowered. Alternatively: Crush 200g add 8 drops of alcohol, warm-up on a pot over the fire. Place emplasto mixture on top of the affected area, then cover with a piece of cloth and then with a piece of plastic. 2 times a week as needed.	Inflammation, Rheumatism, High fever	GER217
Senecio tephrosioides Turcz.	Huamanrripa, Genciana	Whole plant, fresh	Oral	Boil 1 cup of water, then add 10g of Huamanrripa, combined with Veronica, Vira Vira, Brochamelia, and other herbs. Drink 3 cups per day, 15 days.	Bronchitis, Asthma, Pneumonia	JULS12
Smallanthus sonchifolius (Poepp. & Endl) H. Rob.	Hojas de Yacon, Llacon	Leaves, dried	Oral	5g per 1l, drink Three times per day, total of 1l a day.	Diabetes, Kidneys, Inflammation of the prostate, Cholesterol	EHCHL143
Sonchus oleraceus L.	Cerraja, Serraja, Zeraja	Whole plant, fresh	1., 2., 3., 4. Oral	1. Tea, 5g per 1l boiling water mix with Colores, Lancetilla, Contrahierba. Three times per day.<br>2. 1 cup with the whole plant (especially Flowers) cut with scissors, together with 3 drops of lime, a pinch of salt and a shot of pisco, let mixture sit for 3 minutes and take Take 1 cup, 2 times a day for 1 day, until condition passes.<br>3. 1 handful of herb boiled with 1/2l water, 3 cups per day, before food.<br>4. Take 1 whole plant, crush, drink extract, once.	1. Cholera, High blood pressure<br>2. Embarrasment, Shame, Hangover, Negative energy, Anger<br>3. To calm strong character<br>4. Hangover	RBU/PL368, EHCHL54, JULS64, VFCHL48, JULS248
Spilanthes leiocarpa DC.	Turre	1. Flowers, fresh<br>2. Leaves and Stems, fresh	1. Oral<br>2. Topical	1. Crush and grind the plant material, then place on the affected area. Place plant material on affected area while chewing. Apply 2 times a day, as needed.<br>2. Patient should bathe in the solution when it is cold. Do not rinse it off. Patient must air dry. 2 times a week until rash is gone.	1. Toothache, Anesthetic for pain<br>2. Skin rashes	JULS264, GER195
Tagetes elliptica Sm.	Culantrillo Serrano	Whole plant, fresh or dried	Oral	50g of the plant and 1 cup of water and boil for 5 minutes. Drink cold, 1/4 cup a day for 8 days.	Colds, Bronchitis, Congestion	GER184

Family/Genus/Species	Indigenous name	Plant part used	Admin.	Preparation	Use	Coll. #
Tagetes erecta L.	Flores del Muerto, Claveles Chino, Flor de Muerto	Flowers and Leaves, fresh	1. Topcial<br>2. Oral	1. Macerated in any liquid substance (like Agua Florida). With 100g of Parrano, 100g of Frejol chileno ground, 100g of Ajo ground, 5g of Chili powder and 5g of Black Pepper and 5g of Oregano, Flor de Chocho, Retama, Ruda (hembra and macho) and Agua del Susto. As poultice, put a portion on feet and cover with a cloth. It can also be used on the stomach. Alternatively the mixture as bath, 3 times per week, Tuesday, Friday, Tuesday.<br>2. Take 3 to 4 Flowers and boil in 1l of water along with 10g of a mixture of Toronjil, Pimpinela, Poleo, Manzanilla. Drink 3 to 4 glasses a day for 1 month.	1. Fright / Susto, Colic of the stomach, Bad Air / Mal Aire<br>2. Cough, Nerves, Inflammation (general)	EHCHL141, JULS156, GER112
Tagetes filifolia Lag.	Anis, Anis Serrano	Whole plant, fresh or dried	Oral	10g per 1l mixed with Poleo, Manzanilla, Muña or Chancas de Comida, and Hinojo. 3 cups daily for 1 week to 1 month.	Severe colic, Stomach, Stomach pain, Diarrhea	RBU/PL283, JULS8
Tagetes patula L.	Flores del Muerto	Stems and Leaves, fresh or dried	Topical	Limpia, 5g with 3l water mixed with Ajenco, Ruda Hembra, Hierba del Susto, Manzanilla Blanca, and Timolina. 2 times per month.	Susto of Death/ Susto de muerte.	ISA89
Taraxacum officinale Wiggers	Diente de Leon, Amargon, Hierba del Leon	Whole plant, fresh	1., 3. Topical<br>2. Oral	1. 200g leaf and flask of 7 Espiritus. Poultice: 2 times per month.<br>2. 20g leaf, 2l of water with Chacur, Pie de Perro, Cola de Caballo, Linasa, Malva, Amor Seco and other plants. 20g total of all. Add a piece of 4 inches of Bejuca del Contra Aire, and 2 spikes of Palmerilla. Boil for 3 minutes. 1 cup 4 times a day for 1 month.<br>3. Bath mixture for Protection from Evil, see below. Only once.	1., 2. Liver, Stomach, Inflammation (internal), Ovaries, Bad Air / Mal Aire, Protection against evil<br>3. Protection from evil	RBU/PL252, JULS150, GER62, GER189
Tesaria integrifolia R. & P.	Pajaro Bobo	Flowers and Leaves, fresh	Oral	Boil 10g Pajaro Bobo per 1l water. Combine with Cola de Caballo, Verbena, Chacur, Paja Blanca, and Espiga de Maiz. Drink 3-4 times per day for 15 days. Patient should drink hot solution for most ailments, and cold solution for bad breath.	Liver, Kidneys, Gallbladder, Inflammation (general), Fever, Bad breath	JULS71, GER12
Trixis cacalioides H.B.K.	Añasquero Chico	Whole plant, fresh or dried	1. Incense<br>2. Topical	1. Burn 2 times a month. 2. Mix with Añasquero Grande, Ruda Hembra, Ruda Macho, Ajenco, Timolina, Ishpinguillo, Chuque, 7 Espiritus. Take 2 baths per week or use as limpia once a week.	1. Rid the house of negative energy<br>2. Daño, Fright / Susto, Bad Air / Mal Aire	ISA65, RBU/PL295
Weddelia latifolia DC.	Cuchalman	Whole plant, fresh	1. Topical	1. Boil 10g Chulgan with 1l water. Patient should take solution at room temperature, once.<br>2. Boil 1 small bundle of Chulgan with 2l water. Do not mix with other herbs. Patient should drink lukewarm solution, once.	1., 2. Fever	JULS80

Family/Genus/Species	Indigenous name	Plant part used	Admin.	Preparation	Use	Coll. #
Werneria humilis H.B.K.	Señorita	Flowers, Leaves and Stems, fresh	Seguro	3 Flowers or Stems per flask.	Inducement of love in men and women, For a woman to find a good man, Making the feelings of men and women clearer	ISA98
Werneria pygmaea H. & A.	Hierba del Halago	Whole Fruit, fresh	1. Seguro<br>2. Topical	1. Standard Seguro mixture, see below.<br>2. Standard mixture for Spiritual Flowering, see below.	1. Good business, Protection, Good fortune, Good health<br>2. Spiritual Flowering	GER120
Werneria villosa A. Gray	Hierba del Oro	Leves and Stems, fresh	1. Seguro<br>2. Topical	1. Standard Seguro mixture, see below.<br>2. Alternative mixture for Spiritual Flowering, see below. After boiling add a bottle of your favorite perfume. Rub the entire body with all the herbs, then rinse with the water and Air dry. Do not use soap nor a towel.	1., 2. So that all goes well in the life of a person, Home, Good luck, Big enterprise, Personal, Good business, Protection, Good fortune, Good health	ISA101, GER124
BALANOPHORACEAE						
Corynaea crassa Hook. F.	Huanarpo (hembra & macho)	Tuber/Root, fresh	Oral	1 bottle of wine (abuelo), add 10g of Huanarpo, then add Huevo de Angelote, Bee Pollen, honey, Pacra, Palo Sangre, Palo Huaco, Chuchuhuasi, Cascarilla, Para-Para. Drink 3 cups per day, 3-6 months. Take a small cup before intercourse. Can refill the wine bottle once more with the same herbs, it will be stronger. If it for a man, use "macho". If it is for a woman, use "hembra". 3 cups per day, 3-6 months. Take a small cup before intercourse.	Fertility, Sexual potency, Male impotence, Elderly men, Tension	JULS171, VFCHL52
BETULACEAE						
Alnus acuminata H.B.K.	Aliso Blanco (Liso), Aliso Colorado (Arrugado)	Bark, fresh	1., 3. Topical<br>2. Oral	1. Macerate 1kg of Colorado mixed with Aliso Bark per 4l alcohol. Apply to wounds, do not Ingest! Apply until wounds seal, three times a day.<br>2. Boil 10 minutes, 2 Tbsp per cup to get the extract, Take 1 Tbsp every 4 hours.<br>3. Soak Bark in water and use as bath, or grind the Leaves and mix with odorles vaseline. Bathe 3 times a month, or rub daily on the patient until symptoms improve.	1. Sealing wounds, Rashes, Skin irritations, Arthritic pain<br>2. Arthritis, Cold, Colic of the stomach, Colic of the intestine<br>3. Bone pain, Arthritis	ISA18, ISA17, RBU/PL292
BERBERIDACEAE						
Berberis buceronis J.F. Macbride	Palo Amarillo	Wood and Bark, dried	Oral	2g of the Bark in 1l of water. Boil for 3 minutes with a total of 10g of Amor Seco, Cola de Caballo. Drink lukewarm with drops of lime. 3 cups a day for 1 month.	Liver, Hepatitis	JULS285

Family/Genus/Species	Indigenous name	Plant part used	Admin.	Preparation	Use	Coll. #
BIGNONIACEAE						
Crescentia cujete L.	Higueron	Latex from Leaf, fresh	Topical	With white cotton, administer milk of Higueron, forming a ball with the Latex. Apply to the bellybutton and tie it down with pressure. Leave it on for 3 weeks.	Healing of belly button after birth	JULS164
Cydista aequinoctialis (L.) Miers	Bejuco Amarillo	Flowers, Leaves and Stems, fresh	Topical	1 handful in 3l water for 5 minutes. 2 times per month.	Daño, Internal inflammation from sorcery, Bruises froms sorcery	ISA6
Jacaranda acutifolia H. & B.	Arabisca, Yarabisca	Leaves and Stems, fresh or dried	Oral	10g per 1l boiling water, boil 2-3 min. Drink 3 cups per day, as needed.	Cough, Bronchitis, Asthma, Phlegm	RBU/PL326
Tynnanthus scabra (Hoffm. ex Roem. & Schult.) Schum.	Clavo Huasca	Leaves and Stems, fresh	Oral	Blend 100g of plant material with 1/4 glass of water and drain. Drink cold. Used during ritual ceremonies. 1 tablespoon per ritual.	Bad Air / Mal Aire, Hallucinogen, Enhance the vision of shaman	GER224
BIXACEAE						
Bixa orellana L.	Achote, Hoja de Achote	Seeds and Leaves, fresh or dried	Oral	Chop 3 Seeds and eat as needed. Alternatively boil 10g of plant material with 10g of Uña de Gato with 1l of water. Boil the mixture for 3-4 min, mix with Chante because plant is cold. Drink 1l per day for 1 week. Patient should drink warm solution. If possible, use Leaves. The Seeds are les powerful.	Inflammation of the kidneys, Prostate, Food coloring, Bronchitis, Hemorrhages, Pulmonary systems, Urinary infections	ISA126, RBU/PL264, JULS9, EHCHL20, ISA44, JULS293
BORAGINACEAE						
Borrago officinalis L.	Borraja	Whole plant, fresh or dried	Oral	10g herb with 1l boiling water, boiled for 3-5 minutes, combined with Vira Vira. Drink three times per day or 1l per day, as long as needed.	Bronchitis, Lungs, Blood problems, Burn fat, Lose weight, Anxiety, Depression, Heart, Nerves, Insomnia, Cough, Cold, Bruises	ISA112, JULS24, RBU/PL300, EHCHL58
Cordia alliodora (R. & P.) Oken	Ajos Giro, Ajos Quiro, Ajo Sacha	Bark and Stems, dried	1. Topcial<br>2. Oral	1. 5g with Llatama, Ajos Giros, Añasquero grande, Llatama, Hierba del Gallinazo, Añasquero chico, Ruda macho per 3l water. 2 baths per month. Can also be used as steam bath.<br>2. Add 1 bottle of Abuelo wine with 10g of plant material and 20g of Chuchuhasi, Cascarilla, Honey, Pollen, Tutuma. Let the mixture sit for 1 week. Drink the mixture. Patient should not leave the house while taking treatment. Adults take 1 small cup. Children take 1 teaspoon. Patients take the medication 3-4 times a day until the bottle is finished.	1. Daño, Fright / Susto, Dispelling negative energy from the house<br>2. Bronchitis	ISA74, JULS281

Family/Genus/Species	Indigenous name	Plant part used	Admin.	Preparation	Use	Coll. #
Cordia lutea Lam.	Overo, Flor de Overo, Overal	Flowers, fresh or dried	Oral	1 Tbsp per 1l water, use with Llantén, Boldo, boil 5 min. gather Jan-Feb. Drink 4 cups a day for 1 month, after food. after drinking the beverage, eat a lemon candy. Patient must limit physical activity until well rested.<br>Latex of the Fruit is used as paper glue.	Liver, Bladder, Hepatitis, Inflammation of the kidneys, Prostate inflammation.	ISA125, EHCHL77, JULS62, GER10
Heliotropium curasavicum L.	Alacran, Alacrancillo	Whole plant, fresh or dried	Topical	Boil 10g of Alacran with 10g total of Hierba de la Plata, Justicia, and yellow, red, and white roses in 2-3l of water. Bathe 3 times (Tuesday, Friday, and the following Tuesday).	Spiritual Flowering	JULS305
Tiquilia paronychoides (Phil.) Rich.	Flor de Arena, Paja de Lagartija, Mano de Raton	Flowers, fresh or dried	Oral	10-100g of plant material and 10g of Malva, Espiga de Maiz, and Cola de Caballo, Contrahierba, Flor Blanca, Cadillo, Berros, Chante, Achote, Lancetilla, Pomanpara, boil 3-5 minutes, with 1l water. Drink 3-4 times a day, 1l daily, for 2 weeks to 1 month.	Inflammation, Inflammation of the kidneys, Inflammation of the ovaries, Bladder stones, Prostate inflammation, Gallbladder, Urinary infections	JULS154, EHCHL107, ISA58, GER20
BRASSICACEAE						
Brassica oleracea L. f. sp. *capitata*	Col, Repollo	Leaves, fresh	Oral	3 to 4 Leaves of Cabbage in 1l of water with a couple of drops of olive oil. Drink lukewarm. 1 cup 3 times a day for a week.	Gallbladder with stones	JULS147
Brassica rapa L.	Nabo	Root, fresh	1., 2. Topical	1. Grind tuber, drain to extract the juice. Gargle fast with juice 3 times day for 2-3 days.<br>2. Grind 2 big tubers. Place on affected area, cover with a piece of cloth for 5 min. 3-4 times per day for 2 days.	1. Throat infection and inflammation<br>2. Kidney inflammation, Ovaries	JULS201
Capsella bursa-pastoris (L.) Medic.	Bolsita del Pastor, Hierba del Pastor, Bolsa de Pastor	Whole plant, fresh or dried	Oral	10-30g per 1l water, mix with Chacur, Verbena, Espiga de Maiz, Flor Blanca, Cola de Caballo, Flor de Arena, Pasuchaca, Corpus Way, Cola de Caballo, Arenilla. 4 cups per day, 1 month as needed.	Kidneys, Prostate, Inflammation, Inflammation (internal), Liver, Gallbladder, Stomach infection, Urinary tract	JULS7, VFCHL42, VFCHL12, RBU/PL257, EHCHL6
Lepidium virginicum L.	Maipa	Whole plant, fresh	Topical	Boil 1 cup and mix with 2 small branches, or 1 small branch for a blemish on the skin. Wash the wound with water in the morning, afternoon, and night, wash face three times per day.	Pock marks (facial), Sun spots, Malnutrition blemishes, Skin blemishes (facial), Washings (wounds)	JULS45

Family/Genus/Species	Indigenous name	Plant part used	Admin.	Preparation	Use	Coll. #
Raphanus sativus L.	Rabanito	Tuber, fresh	1., 2. Oral	1. 1/4kg of sugar, add 1/2kg of Rabanito cut in pieces. Boil with a scallion with no water. The syrup becomes a drink for the patient.1 Tablespoon evey 6 hours for 1 month.<br>2. Blend about 50g of raddish. Drink cold in the morning while fastingglass 1 time a day for 15 days. Drink in the morning while fasting.	1. Bronchitis<br>2. Clean blood from toxins, Cleansing of the liver, Erase stains from the face	JULS238, GER202
Rorippa nasturtium-aquaticum (L.) Hayek	Berros	Whole plant except root, fresh or dried	Oral	Oral fresh as needed or crush and drink juice with Alfalfa. Make a soup with the nape of the neck of the sheep and boil. Add potatoes and veggies. Alternatively boil 1l of water with Berros, plus 10g total of Malva, Pie de Perro, Unquia, Amor Seco, Chacur, PajaBlanca, Flor de Arena, Puren Rosa, and other herbs. Boil for 3 to 4 minutes. Drink 3 to 4 times a day for 1 month.	Liver, Urine retention, Bronchitis, Kidneys, Inflammation of the liver, Inflammation of the kidneys, Anemia	RBU/PL367, EHCHL25, JULS113
BROMELIACEAE						
Ananas comosus (L.) Merrill	Piña	Fruit Peel and Fruit, fresh	Oral	Whole pineapple Peel, add 1l of water and boil for 3 to 4 minutes. Drink hot, 1 cup 3 times a day as needed. Also drink one glass of fresh juice daily.	Burn fat, Lose weight	JULS230
Puya hamata L.B. Sm.	Hierba del Carnero, Hierba de Borrego	Hairy part of the Seeds, dried	1. Oral<br>2. Topical	1. 1 cup of water and 5g of the plant (the hairy part of the Seeds being the most important) and boil for 3 minutes. Drink 1 cup twice a day 3 to 4 times a week. This seguro is used to get the patient under control, usually if the patient is being violent or out of control because of drunkenness or other reasons.<br>2. Same mixture can be applied as poultice.	Making a man stupid, Making a man obey like a sheep, Cleansing, Controlling a violent person, Dominating a drunk, Tumors, Infections	JULS162, GER95, EHCHL121
Puya weberbaueri Mez.	Ticta, Tifta	Whole plant, fresh or dried	Topical	Boil 15g of Ticta and 10g of Hierba Santa with 3l of water. Boil the mixture for 3-4 minutes. Bathe the patient in the mixture. Patient can bathe on any day. Bathe once a week, for 1 month.	Bad Air / Mal Aire, Wounds, Any illness involving wounds	JULS290
Tillandsia cacticola L.B. Sm.	Palmera, Siempre Viva, Palma Bendita, Siempreviva (lilac)	Leaves and Stems, fresh	1. Topical<br>2. Oral<br>3. Seguro	1. 50g in fire combined with Romero, Palo Santo, Alucema, Incense, Saumerio, and Myrrha. As bath: Alternative mixture for Spiritual Flowering, see below. Steam bath, as needed or bath one time per day for 15-30 days.<br>2. 20g per 1l water, boil 2 min. Combine with Pimpinela, Cedron, Mejorana, Simpre Viva, Flores de Diamelas, Toronjil, Romero, Claveles, Orange Flowers. 1l per day or 3-4 cups per day after meals.<br>3. Standard Seguro mixture, see below.	1., 2. Fright / Susto, Heart, Gases, Nerves, Anxiety, Heavy air, Good luck, Susto of Death / Susto de muerte, Spiritual Flowering, Good business, Protection, Good Luck, Good health<br>3. Good business, Protection, Good luck, Good health	VFCHL17, RBU/PL375, RBU/PL289, GER123, JULS307

Family/Genus/Species	Indigenous name	Plant part used	Admin.	Preparation	Use	Coll. #
Tillandsia multiflora Bentham. var. *decipiens* (Andre) Sm.	Siempre Viva, Siempre Viva Roja	1. Flowers, fresh<br>2. Whole plant, fresh	1. Oral<br>2. Topical	1. 10g per 1l boiling water. Combine with Toronjil, Clavelin, and Lima juice. Take 3 times a day for 1 month.<br>2. 20g per 5l water and boil for 20 minutes. Bathe 3 times a week.	1., 2. Depression, Heart, Nerves	EHCHL15, RBU/PL376
BURSERACEAE						
Bursera graveolens (H.B.K.) Triana & Planchon	Palo Santo, Palo de Santo	Small Stems, Bark and Wood, dried	1. Topical<br>2. Oral<br>3. Incense	1. 3 Tbs per 3l water. Combine with Romero Blanco and Romero Castilla. Bathe 2-4 per month.<br>2. Boil 1l of water, then add 2 pieces of about 5-10g of the Palo Santo, boil for 5 minutes. Cover and let it sit for 3 minutes. Drink hot, 1 little glass 3 times a day for 2 days only.<br>3. Big house: use 250g of herb. Small house: 20g. Combine with Romero Blanco, Romero de Castilla, Romero, Hierba de la Plata, Hierba de la Fortuna, and Hierba de Oro, incense and myrrha. Every Tuesday and Friday as needed. For people: Patient must be naked with a piece of cloth tied to the neck. Place the jar with the smoking Palo de Santo under the person (feet) letting the smoke rise. Also used in animal corrals mixed with Palo Huaco to keep insects away.	1. Daño, Fright / Susto, Sorcery<br>2. Cough, Flu, Bronchitis, Cold<br>3. Dispelling negative energy from the house, Bad shadow	ISA143, JULS210, GER34
Commiphora myrrha (T. Nees) Engl.	Mirra	Latex, dried	Incense	Burn it into charcoal and mix with 10g of Myrrha plus Palo Santo, Saumerio, and Romero. Burn like an incense and spread smoke around the patients house. 3 times a week: Tuesdays - Friday - Tuesday. Repeat as necessary.	Dispelling negative energy from the house	JULS195
CACTACEAE						
Echinopsis pachanoi (Britton & Rose) Friedrich & G. Rowley	San Pedro, Huachuma	Whole plant, fresh	1. Oral<br>1., 2. Topical	1. Chop San Pedro in thin slices horizontally and boil in 4l of water from 12 noon to 6pm. Cook on low fire and add water if necessary. 1 glass for the patient and 1 glass for the shaman per ritual session. Cannot eat fat, spices (such as aji), fish or shellfish, for 24 hours after drinking. Apply topical for wounds and acne. Patient should stay away from the sunlight for 24 hours.<br>2. Apply pulp onto the scalp.	1. Ulcers, Hallucinogen, Enhancing vision during rituals, Wounds caused by Mal Daño, Bad Air / Mal Aire, Inflammation (general), Acne<br>2 .Washing hair, Fortifying hair	TRUBH36, JULS242, GER73
Opuntia ficus-indica (L.) Miller	Tuna	1. Fruits, fresh<br>2. Leaves, fresh	1. Oral<br>2. Topical	1. Fruits Peeled and extracted. Drink the extract. Take 1 glass per day, as needed.<br>2. Cut a leaf in half (horizontally), boil each half in 3l of water for 20 minutes and drain. Wash hair with preparation and rub scalp as a shampoo. Bathe once a day for 2 days. Repeat once more. Total of 4 baths.	1. Diabetes<br>2. Hair loss	JULS263, GER3

Family/Genus/Species	Indigenous name	Plant part used	Admin.	Preparation	Use	Coll. #
CAMPANULACEAE						
Centropogon articulatus Drake	Conchalalay	Stems and Leaves, fresh or dried	Topical	20g per 5l water, boil 20 min. Bath, 1-3 times per month.	Fright / Susto, Air / Aire	EHCHL119
Centropogon cf. *cornutus* L.	Raínga	Leaves and Stems, dried	Oral	Boil 1 cup of water with 100g of the plant. Drink cold. Once a day. It has to be followed by other treatments with other herbs.	Bad Air / Mal Aire, Dissolve/remove tumors	GER78
Centropogon cf. *rufus*. Wimm.	Trinoso	Leaves and Stems, fresh or dried	Oral	10g of each of the following: Cadillo, Amor Seco, Lampazo into 1/2l of water and boil for 5 minutes. Drink lukewarm, 1/2 cup 3 times a day for 20 days or as needed.	Intestine, Liver disease, Gallbladder disease, Tumors, Urinary disease, Skin	GER210
Lobelia decurrens Cavaniles	Contolla	Whole plant, fresh	Oral	Boil 1l of water, then add 5g Contolla. Drink 1 cup a week for up to 1 month. Alternatively empty a cigarette 75%, fill up to 25% with ground Contolla, and refill remaining 75% with tobaco, smoke.	Curing drug addicts. Causes vomiting and diarrhea.	JULS277
Siphocampylus angustiflorus Schlechtendal	Contoya, Hierba de Envidia, Contolla	Flowers, Leaves and Stems, fresh	1. Oral<br>2. Topical	1. 5g per 1/2 cup with boiled water, drink 1 per month.<br>2. 20 minutes, with 10g of herb per 10l water mixed with other herbs of luck. Bathe 3 times per week.	1. Purgative<br>2. Daño, Cast away envy	RBU/PL364, EHCHL99
Siphocampylus cutervensis A. Zahlbr.	Conchalalay Blanco	1. Leaves, fresh<br>2. Leaves, dried	Topical	1. Poultice, 1 time per month.<br>2. 1 handful or pouch with 3l boiled water. Combined with Conchalay Colorado, Guaminga, Bully Vinegar, and 7 Espiritus. 2 baths a week, in agreement with what the Mesa indicates. As limpia 2 times a month.	1. Concuisions<br>2. Freight/ Susto, Daño	ISA2
Siphocampylus tupaeformis Zahlbr.	Cochaya	Whole Fruit, fresh	Charm	Do not ever cut the plant. use it in its natural form by planting it in the area around your house. Always plant Cochaya close to a San Pedro. Always have a few on your property. If a thief comes onto your property this plant will start to release snakes all around and tie the person up without ropes. A thief can go crazy. This plant always detects who is doing bad things and recognizes its owner and its family.	Guarding the house and land.	GER102

Family/Genus/Species	Indigenous name	Plant part used	Admin.	Preparation	Use	Coll. #
CAPPARIDACEAE						
Capparis crotonoides H.B.K.	Simuro, Bichayo, Simulo	1. Flowers, fresh<br>2., 3., 4. Leaves, fresh	1. Oral<br>2., 3., 4. Topical	1. Boil 10 Flowers buds in 1/2 cup of water for 2 minutes. Patient should drink warm solution and stay inside the house during treatment. 1 cup a day for 8 days.<br>2. Boil 2kg of Bichayo Leaves in 5l of water for 30 min. The patient must be naked with a towel over his head in an enclosed room. Patient must take deep breath/inhalations. Bath should last about 1/2 hr. 1 bath every 6 days, 2 times only.<br>3. Crush 20 Leaves of Bichayo. Place crushed Leaves on affected area and masage the area with it. Patient should not go out during treatment.<br>4. Add 20g of plant material into 4-5l of water. Boil the mixture for 5-6 minutes. Bathe with the tizana. Do not ingest the mixture. Bath 2-3 times, as needed.	1. Bronchitis<br>2. Arthritis, Rheumatism<br>3. Cold, General pain: muscular, bone, etc.<br>4. Bad Air / Mal Aire, Colds	GER4, JULS250
Capparis scabrida Kunth	Zapote	Fruits, fresh	Oral	Blend Fruit and collect extract. Drink temperate. 1 glass a day for 4 days.<br>Also used as glue extracted from the trunk of the tree.	Inflammation (general), Heart palpitation, Refresh liver, Reduces anxiety, Also good to increase milk production in cows.	GER33
CAPRIFOLIAEAE						
Lonicera japonica Thunberg	Madre Selva	Whole plant, fresh or dried	Oral	Boil 1l water, then add 10g Madre Selva. Drink 3-4 cups per day for 1-3 months, or as needed. Epilepsy is characterized by heart pains, frequent falling to the ground, and los of consciousnes every 3 days.	Depression, Heart, Pain of love, Nerves, Epilepsy, Psychological pain	JULS28
Sambucus nigra L.	Cinta de novia	Stems, fresh	Charm	Tie a picture on the Stems and wrap it. Pray and spray (fogear) perfume with the appropriate names. One per ritual. Repeat as needed. Ritual "Amarres" is for tying up a person, binding someone.	To tie a person	GER220
Sambucus peruviana H.B.K.	Sauco, Saucotillo	1. Leaves, Flowers and Stems, fresh or dried<br>2., 3. Leaves, fresh<br>4. Flowers and Leaves, fresh	1., 4. Oral<br>2., 3. Topical	1. 5-20g per 1l, boil for 1 min, as tea, combine with Llonque. 3 times per week, up to 1l per day if needed, or until fever passes. Take while cold. Rub with Llonque.<br>2. Bath. Combine with Nogal, Hierba del Susto, Manzanilla Blanca with a flask of Timolina. 2-4 times per month, not to be used too much because it is very cold.<br>3. Poultice, one time per month.<br>4. Boil 1l of water, then add 10g of Sauco. Add Manzanilla, Hinojo, Coleo, Ajenjo, Toronjil, Pimpinela and Claveles. Cover and let it sit for 2-3 minutes. Patient should drink warm solution, 3-4 cups per day for 1 month.	1. Swelling, Kidneys, Cough, Concussions, Prostate, Fever, Bronchitis, Yellow Fever<br>2. Fright / Susto, Fever, Yellow Fever<br>3. Inflammation of the kidneys, Gastritis<br>4. Nerves, Cough, Cold, Fever, Insomnia	EHCHL140, RBU/PL291, VFCHL44, ISA131, ISA87, JULS246, EHCHL110

Family/Genus/Species	Indigenous name	Plant part used	Admin.	Preparation	Use	Coll. #
CARICACEAE						
Carica papaya L.	Papaya	1. Seeds and Fruit Peel, fresh<br>2. Fruit	1., 2 Oral	1. Blend 10l of water, 1 cup of Seeds and a whole Peel of a small papaya. Drink while fasting, 1 glass 3 times a month. "This will make you vomit and defacate a lot"<br>2. 1 small bowl everyday for 1 week before breakfast. Do not add lime, fast.	1. Parasites of the stomach, Laxative, Anti-venom, Revert poison effect<br>2. Inflammation of the liver	JULS215, GER204
Jacartia digitata (Poepp. & Endl.) Solms-Lang.	Contra Hechizo	Root, fresh	1. Oral<br>2. Topical	1. Boil a 25cm portion of root boiled with 3l water, 20 min or crush 200g of Contra Hechizo adding 50g of sugar. Drain the extract. 1 glass during the session, in agreement with the patient.<br>2. Crush Stems of Contra Hechizo and drain extract. Apply on face or affected areas as a cream. 2 a day: AM and before going to bed for 6 days or until acne disappears.	1. Purgative (Daño), Laxative for people who suffer from colic pain and gases<br>2. Acne	EHCHL70, GER72
CARYOPHYLLACEAE						
Dianthus caryophyllus L.	Claveles, Clavelina, Clavel de la Costa	Whole plant, fresh	1. Topical<br>2. Oral	1. Boil 7 entire plants with Hierba de la Plata, Hierba de la Justicia, Ruda, and Romero per 3l of water for 5 minutes. Bath as needed on special days depending on the phases of the moon. 1-3 times per month depending on the individual.<br>2. 50g of the Claveles (white, red, yellow, and purple petals), 5g of sugar and 1/2 cup of water and boil for 2 minutes. 3-4 cups per day, 1 month.	1., 2. Pain of love / Mal de amor, Sentimentality, Heart, Nerves, Good luck, Insomnia	JULS18, JULS37, GER214
Dianthus caryophyllus L.	Clavel Serrano	Whole plant, fresh	Oral	Add 50g of the plant material, 50g of Huamanrripa and 1 cup of water. Boil the mixture for 5 minutes. Drink the mixture cold. Take 1/4 of a cup once a day, for 30 days.	Heart disease, Heart palpitations	GER180
Stellaria media (L.) Criollo	Tripa de Cuy	Whole plant, fresh	Oral	Add 10g of plant material with 1l of water, Malva, Amor Seco, Chacur and Unguia. Drink 1 cup, 3-4 times a day, for 1 month.	Inflammation of the kidneys, Renal disease	JULS262
CHENOPODIACEAE						
Chenopodium ambrosioides L.	Paico	Leaves and Stems, fresh	Oral	1. Extract the juice of the Leaves. Oil of the Seeds and Fruit has an ingredient that kills parasites. Use 1 time per month.<br>2. Add 10g of plant material with 1/2l of water. Drink hot, 1 cup, 2-3 times a day for 1 week.	1. Parasites (worms)<br>2. Cough	EHCHL112, RBU/PL280, EHCHL53, JULS206
Chenopodium quinoa Willd. (wild form)	Quino Amargo	Seeds, fresh	Topical	1 Tbsp or 5g per 1l. can mix with el Chocon, once a month as enema.	Intestines (cleansing), Stomach (cleansing)	EHCHL160
Chenopodium quinoa Willd.	Quinua	Seeds, fresh or dried	Oral	Boil 1l of water, add 150g of Quinua and boil for 10 minutes or until the grain is soft. Add a piece of cinnamon and a piece of apple. Take 1 cup, 1 to 2 times a day as needed.	Nutritional supplement	JULS236

Family/Genus/Species	Indigenous name	Plant part used	Admin.	Preparation	Use	Coll. #
CHLORANTHACEAE						
Hedyosmum racemosum (R. & P.) G. Don.	Masamoche, Asancito, Asarcito, Asarquiro, Choleta	Bark, dried	Oral	Use outside of Bark. 8-10g per 2l water, boil 20 min. drink as needed. Alternatively 30g per two bottles of alcohol mixed with Chuchuwasi, Cascarilla, 7 Raices, and Huayacanes then allow to sit for 8 days. Drink as needed, but do not drink before it has sat 8 days.	Bronchitis, Cold, Cough, Asthma, Rheumatism, Bone pain, Nervous system	EHCHL147, RBU/PL377
CHRYSOBALANACEAE						
Coupeia sp.	Acharachango, Charachango, Ashango	Seeds, dried or fresh	1. Topical<br>2. Oral<br>3. Blown on patient	1. Bath, 20 Seeds per 5l water, ground and boiled. 3 times per week, or 2 a month, Tuesday and Friday.<br>2. Beverage: 7-15 Seeds per 1l water, crushed and macerated for 8 days. 3-4 small cups per day, 7 days. Seeds can be also macerated in alcohol for 5 days, then 1 Tbsp, 3 times per day.	1., 2., 3. Fright / Susto, Bad Air / Mal Aire, Nervous system, Enchantment, Sorcery, Getting rid of daño, Epilepsia	EHCHL157, RBU/PL381, GER68, VFCHL54
CLETHRACEAE						
Clethra castaneifolia Meissner	Hierba del Olvido	Leaves and Stems, dried	Seguro	Put together in a cloth 10g of Hierba del Olvido, 10g of Hierba del Demonio, and Hierba de la Justicia. Seal and pray. Patient must carry the bag and pray.	Dominating judgment (ritual), Dominating legal problems (ritual)	GER115
CLUSIACEAE						
Clusia minor L.	Chusgon	Whole Fruit, fresh or dried	Oral	Boil 1l of water, then add 10g of Chusgon. Add Pimpinela, Manazanilla, Toronjil, Barrojo, Romero, and Chancas de Comida. Let it sit for 2-3 minutes. Patient should drink warm solution. 1 cup 4 times a day as needed.	Nerves, Heart disease	JULS280
Hypericum aciculare Kunth.	Hierba de las Cordilleras, Lechuguilla, Hierba de Iman	Leaves and Stems, fresh	1. Seguro<br>2., 4. Topical<br>3. Oral	1. 1 small Stem.<br>2. Mix with Llonque, 7 Espiritus, Hierba Santa. Limpa twice a week.<br>3. 3 Leaves, chopped and made into extract. No Mixing! 1 Tbsp per day, 8 days.<br>4. Natural, with 7 Espiritus. Apply as poultice for 4 hours.	1. So that you should get all you want<br>2. Sorcery, Headache<br>3. Fever, Intestinal fever	ISA135, ISA35, JULS301
Hypericum laricifolium Jus.	Hierba de Cariño, Hierba de la Fortuna, Solitario, Chinchango, Abrecaminos	Whole plant, fresh	1. Topical<br>2. Seguro	1. Alternative mixture for Spiritual Flowering, see below. Take 3 baths per month.<br>2. Standard Seguro mixture, see below.	1. Fragrance, Luck in love and work, Bad Air / Mal Aire, Love, Business Relations, Protection, Good fortune, Good health<br>2. Good business, Protection, Good fortune, Good health	RBU/PL344, TRUBH1, TRUVan/Erica22, EHCHL145, GER128, GER126
Hypericum silenioides Jus.	Cintaura	Whole plant, fresh	Oral	3-5g of herb per 1l of water mixed with Culein, Hierba del Toro. Drink three times per day for five days or as needed.	Diarrhea, Dysentery	EHCHL85

Family/Genus/Species	Indigenous name	Plant part used	Admin.	Preparation	Use	Coll. #
Mammea americana L.	Mamey	1. Fruit Peel, fresh<br>2. Leaves, fresh	Oral	1. Boil 1 cup of water, then add 1/4 of the Mamey Fruit Peel. Cover and let sit for 2-3 minutes. Patient should drink warm solution. 1 glass 2-3 times a day for 2 days.<br>2. Boil 1l of water with 4 Mamey Leaves for 3 to 4 minutes. Drink 1 glass 3-4 times a day for about one month.	1. Diarrhea<br>2. Weight loss	JULS190
CRASSULACEAE						
Echeveria peruviana Meyen	Pinpin, Siempre Viva, Rosa Berta, Haya Rosa	Leaves, fresh	1. Oral<br>2. Topical	1. 2 Leaves per 1/2l water, boil 5 min. 1l daily or three times per day.<br>2. Heat over fire then sqeeze Leaf for liquid. Place ten drops in ear everyday or as needed.	1. Inflammation of the kidneys, Nerves, Heart, Liver inflammation<br>2. Ear aches	EHCHL118, VFCHL33, JULS249
CONVOLVULACEAE						
Ipomoea batatas (L.) Lamarck	Camote	Whole plant, fresh	Oral	First wipe the patient with the plant, making the sign of the cross over her chest. Then wash and place plant in 1 cup of water and heat. Drink warm 1 cup 2 times a day for 2 days only.	Promoting lactation in women after giving birth	JULS120
Ipomoea pauciflora M. Martens & Galeotti	Huanarpo	Whole plant, fresh	Oral	Put together in a bottle of cañazo (Yonque) 20g of the plant material plus 20g of Cascarilla, Diego Lope, Hualtaco. Let it sit for 8 days. Drink temperate 1 small cup once a day or as needed (max 2 days only).	Chills, Colds	GER222
CUSCUTACEAE						
Cuscuta foetida H.B.K.	Yodo	Whole plant, fresh	1. Oral<br>2. Topical	1., 2. 1 Tbsp boiled with 1 cup water. drink 1 cup per day or apply as poultice.	Goiter	ISA39
CUCURBITACEAE						
Citrullus lanatus (Thunberg) Matsumura & Nakai	Sandía	Bark, dried	Oral	Peel the Fruit take the pulp and blend. Drink cold, 1 glass fasting for 20 days.	Blood circulation, Refresh heart	GER234
Cucumis dipsaceus Ehrenb.	Jaboncillo de Campo, Jaboncillo del Campo, Jaboncillo, Patito de Campo	Fruits, fresh	Topical	Use Fruit as a shampoo and wash. Open the Fruit and rub the scalp with the Seeds. Bath using the Fruit to rub the whole body. Rub one half of the Fruit on the breast of a breastfeeding mother. Wash 2-3 times a week, as needed.	Dandruff, Adding shine and beauty to hair, Hair loss (prevention), Stopping baby from breastfeeding	JULS174, GER35, JULS221
Cucumis sativus L.	Pepinillo	Whole Fruit, fresh	Oral	Remove the Peel, chop the Fruit finely and add a few drops of olive oil and a pinch of salt. extract juice or eat as salad. Drink cool, fasting 1 glass a day as needed, or eat a small bowl. Oral once a day for a week.	Burn fat, Lose weight, Inflammation of the liver, Indigestion, Heart burn, Intestinal acidity	JULS224, GER209

Family/Genus/Species	Indigenous name	Plant part used	Admin.	Preparation	Use	Coll. #
Cucurbita maxina Duch.	Zapallo	1. Flowers and joints of Stems, fresh or dried<br>2. Seeds, dried<br>3. Leaves, fresh	1., 2. Oral<br>3. Topical	1. Boil 10 Flowers with Leaves and Stems in 1/2l of water for 15 minutes. 1 cup 2 times a day or as much as wanted for 2 days.<br>2. Oral only in the morning with a glass of water. Take 10 Seeds the first day. Reduce amount by one Seeds each day until, on the 10th day. Take a break of one week before repeating process. Process may last 2 weeks to a month.<br>3. Chop 3-4 big Leaves. Place chopped Leaves on affected area and cover with a piece of plastic, then a piece of cloth. Apply for 2 days, then remove.	1. Preventing miscarriage, Inflammation (general), Anxiety<br>2. Heart disease<br>3. Warts, Moles	JULS272, GER32
Cucurbita moschata Duch.	Zapallo	1. Flowers and joints of Stems, fresh or dried<br>2. Seeds, dried<br>3. Leaves, fresh	1., 2. Oral<br>3. Topical	1. Boil 10 Flowers with Leaves and Stems in 1/2l of water for 15 minutes. 1 cup 2 times a day or as much as wanted for 2 days.<br>2. Oral only in the morning with a glass of water. Take 10 Seeds the first day. Reduce amount by one Seeds each day until, on the 10th day. Take a break of one week before repeating process. Process may last 2 weeks to a month.<br>3. Chop 3-4 big Leaves. Place chopped Leaves on affected area and cover with a piece of plastic, then a piece of cloth. Apply for 2 days, then remove.	1. Preventing miscarriage, Inflammation (general), Anxiety<br>2. Heart disease<br>3. Warts, Moles	JULS272, GER32
Cyclanthera pedata (L.) Schrad.	Caihua	Whole plant, fresh	Oral	Blend/liquify 3 Caihuas + 10g Rutabaga + 10g of Carrots + 10g Radishes. 1 8oz glass per day for 15 days in the morning while fasting. Drink cold.	Tonic for the brain, Renal inflammation, Brain Disease, Brain pain, Headache	GER150
Sechium edule Swartz.	Caihua Chilena, Mochera, Caihua	Fruits, fresh	Oral	1/2 of the Fruit chopped, add 10g of Moradilla. Boil in 1l of water for 3 to 5 minutes. Drink 3 to 4 times a day for 1 month.	Blood circulation, Diluting blood clots in order to treat varicose veins	JULS119
Sicana odorifera (Vell.) Naud.	Secana	Whole plant, fresh	Charm	Sleep with plant material and wash it every morning. Do not eat the plant. At the end of thetreatment, the Secana will be wrinkled. Dispose of it in a far away place. Keep the Secana for 7 days and 7 nights.	Jealousy	JULS247
Sycos baderoa H. et A.	Fuque	1. Seeds, dried<br>2. Leaves, fresh	1. Oral<br>2. Topical	1. Grind 100g of Fuque, and add 100g of Cuncuno and 5g of cooking oil. Drain with a bit of water. Drink cold at 6:00 in the morning while fasting. Half a small glass for 4 days.<br>2. Boil 100g of the leaf with 1l of water until all the liquid is gone. Place hand, face, or affected area over the boiling pot and let the steam touch the affected area. Then take the Leaves and place them on top of the affected area while hot. Once a day for 15 days. Use steam therapy for 20 minutes and leave the leaf on top of the mold for 3 hours.	1. Snake bites<br>2. Getting rid of skin molds	GER99

Family/Genus/Species	Indigenous name	Plant part used	Admin.	Preparation	Use	Coll. #
CUPRESSACEAE						
Cupressus lusitanica Miller	Cipre, Cipres	Whole plant, fresh	1. Oral<br>2. Topical	1. 3 branches per 1l water, boil 20 min, drink 2 times a day for 1 week.<br>2. 1/2l of water and 10g of Cipres, boil for 3 minutes. Bathe and wash face with warm solution. Do not rinse after the solution is put on the face. 3 times a day for a week. For hair loss boil 1l of water add 10g total of Amor Seco, Romero, Cola de Caballo, and Cipres and boil for 3 minutes. Wash hair with solution cold solution once a day as needed.	1. Vaginal hemorrhage, Hemorrage<br>2. Acne, Hair loss	RBU/PL288, JULS302
CYPERACEAE						
Cyperus articulatus L.	Baston de San Jose, Varita de San Jose, Pura pura	Whole plant, fresh	1. Seguro<br>2. Topical	1. Add plant material with Hierba de la Plata, Hierba de la Justica, El Dolar, Carpintero, Chupa Flor, Señorita, Hierba de Oro, Fortuna, Agua Florida, perfume, Remillete de Novia, Lima juice, white sugar, and Holy water. Put all materials in a bottle: first herbs, then remaining materials, and cover the mixture. Only the patient can touch the seguro. It is kept within the house.<br>2. Boil the plant material with Hierba de la Plata, Hierba de la Justica, El Dolar, Carpintero, Chupa Flor, Señorita, Hierba del Oro, Fortuna, Roses (various kinds), Ruda (Hembra and Macho), and 3l of water. Boil the mixture for 10 minutes to make solution concentrated. The patient is rubbed with Flowers while bathing in the lukewarm mixture. Bathe Tuesday, Friday, and the following Tuesday, which represents 1 cycle. The bath should be before noon, at 3PM or at midnight. Patients should bathe for 3 cycles.	1. Protection<br>2. Spiritual Flowering	JULS267
Kyllingia pumila Michx.	Hierba de la Golondrina	Whole plant, fresh	Topical	Boiled 20 minutes, 30 g per 6l water with other herbs of luck. Bathe 3 times a week.	Heart, Nerves, Luck, Daño toward a woman	EHCHL73
Oreobolos goeppingeri Sues	Hierba Chupaflor, Hierba de Suerte, Hierba del Carpintero	Leaves, dried	1. Topical<br>2. Seguro	1. 20-50g per 3-5l, boil 20 min. Mixed with herbs of luck and herbs of strength. Bath, 3 times per week. Alternatively: Alternative mixture for Spiritual Flowering, see below. 1 time only.<br>2. Standard Seguro mixture, see below.	Success, Bad Air / Mal Aire, Good luck, Work, Aphrodisiac, Good business, Protection, Good fortune, Good health	EHCHL149, TRUVan/Erica17, EHCHL67, GER119

Family/Genus/Species	Indigenous name	Plant part used	Admin.	Preparation	Use	Coll. #
Scirpus californicus (C.A. Meyer) Steudel subsp. *tatora (*Kunth) T. Koyama	Balsa, Totora	1. Whole plant, dried<br>2. Heart of the stem, fresh	1. Topical<br>2. Charm<br>3. Oral	1. Burn whole and create ash. Apply powder from burned ashes to infected area and scrub hard. 3 times a day as needed.<br>2. Make a small tool, like a brush, of the insides of the Stems. Make the sign of the cros with the brush on the patient and wipe the brush on them. After use, burn the plant. If it falls apart it means the child had mal ojo. Note that this is used only for children.<br>3. 1/2 cup of water add 10g of Totora, 10g of Saze and boil for 3 minutes. Drink cold, 1/2 cup a day for 8 days.	1. Athlete's foot<br>2. Hangover, Evil eye/ Mal ojo<br>3. Fever, Colds	JULS111, GER169
DIOSCOREACEAE						
Dioscorea tambillensis Kunth	Papa Semitona	Tuber, fresh	Oral	1l of water add 1 Papa (usually 10g), with 20g total of Amor Seco, Chacur, Cola de Caballo, Pie de Perro, Verbena, Linaza, and seperately toasted Cebada. Boil for 2 to 3 minutes. Drink lukewarm. One can also add sugar or limes for flavor. 3 times a day, 1 cup, for 1 month.	Inflammation of the kidneys, Inflammation of ovaries, Inflammation of the liver, Inflammation (internal)	JULS283, GER140
Dioscorea trifida L.f.	Papa Madre, Papa Pacta	Tuber, fresh	1. Oral<br>2. Topical	1. Boil 1l of water per 1/4 of a big tuber, for 5 min with Flor Blanca, Purenrosa, Pacharosa, 10g of Watercrest. Drink lukewarm 3 to 4 times a dayfor 1 week or as needed. Especially for children 3 months - 5 years.<br>2. Boil 20g per 1l of water for 20 minutes. Combine with Matico, Malva, and Tara. 1l per day, 15 days.	1. Inflammation, Renal disease, Uterus disease and discharge, Cysts, Internal Inflammation, Cancer of the Uterus, Inflammation of the ovaries, Vaginal discharge, Inflammation of the kidneys<br>2. Fungus, Vaginal cleansing, Cancer of the Uterus, Washings (wounds).	JULS214, EHCHL40, JULS212, GER142, JULS213
DIPSACACEAE						
Dipsacus jallonum L.	Cardo Santo	Whole plant, fresh	Oral	3-5g per 1l water, mix with herbs that are used for the same things. Drink 3 times per day.	Diabetes, Liver, Cholesterol	EHCHL90
Scabiosa atropurpurea L.	Ambarina, Ambarina Negra, Flor de Ambarina, Ambarindas	Flowers, fresh	1., 2. Oral<br>2. Inhaled	1. Boil 1l of water with 20g of the plant material and Estilo, Veronica, Hierba del Toro, Moradilla, Lancetilla, Hierba de la Rabia. Drink hot Drink 3 times a day as long as the disease lasts.<br>2. Chop and mix with maternal milk. Inhale 1 Tbsp daily for 8 days through the nose and also take orally.	1. Whooping cough, Cold, Cough, Bronchitis, Blood Cleansing, Compulsive cough<br>2. Menstrual regulation	JULS100, EHCHL111, RBU/PL372, ISA50
ELAEOCARPACEAE						
Vallea stipularis L.f.	Chuingue	Leaves, fresh or dried	Topical	1 handful per 3l water, mix with Timolina, use for limpia. Alternatively mix maternal milk together with the leaf and put in the ears.	Daño, Hearing problems, Deafness	ISA32

Family/Genus/Species	Indigenous name	Plant part used	Admin.	Preparation	Use	Coll. #
EPHEDRACEAE						
Ephedra americana H. & B.	Diego Lopez, Suelda con Suelda	Whole plant, fresh or dried	1. Oral<br>2. Topical	1. 20g per 2l, boil for 5 minutes. Drink three times per day, 1-2 cups per day, no more than 2 weeks.<br>2. Grind plant, mix with the fat of a male donkey and place around limb. Wear every day for the entire day until bone mends.	1., 2. Bruises, Fractures, Broken bones, External injuries	EHCHL150, JULS38, GER75
EQUISETACEAE						
Equisetum bogotense (H.B.K.) Kunth	Cola de Caballo, Cola de Caballo (Hembra)	Stems, dried	1. Oral<br>2. Topical	1. 1 Tbsp per 1l boiling water, used with herbs Overo, Lancetilla, Chante, Ochote and Zarzaparrilla. Drink 1l daily, 1 month.<br>2. Tizana to wash wound: 10g of this herb and the herbs Pie de Perro, Chacur, and Uncia mixed all together, with 1l water.	1., 2. Inflammation of the kidneys, Wounds (cleansing), Stomach, Urinary tract, Kidneys, Kidneystones, Inflammation (general)	ISA52, TRUVan/Erica6, ISA107
Equisetum giganteum (Wedd.) Ulbrich	Limpia Plata, Cola de Caballo, Tembladera	Whole plant, fresh	1. Oral<br>2. Topical	1. Boil 10-20g per 1l water. Combine with Verbena, Manzanilla, Chacur, Unquia, Espiga de Maiz, Paja Blanca, Berro, Pata de Perro, Papa Madre, Peloza de Choclo and other herbs that are good for the kidneys. Mix with Chante and Ochote for prostate. Boil 2-5 min., drink 4 cups per day, 1 month.<br>2. Boil 10g Limpia Plata with 1l water. Combine with Chacur, Verbena, Hierba Santa, and Llantén. Clean wound with Leaves. clean exces with gauze. Use lukewarm water. Twice a week until the wound heals. Plant should be used to wash wounds, not as poultice.	1. Arthritis, Kidneys, Hemorrhages, Menstrual inflammation, Internal and Extrenal Inflammation, Prostate, Kidneystones<br>2. Wounds (cleansing)	VFCHL1, JULS5, TRUBH19, GER149
ERICACEAE						
Bejaria aestuans L.	Pullunrosa, Cadillo, Payama, Hierba de la Postema, Purenrosa, Hierba de la Postema Rosada, Pulrosa, Hierba del buen querer	Flowers, Leaves and Stems, fresh or dried	1. Oral<br>2. Seguro	1. 5g per 1l, boil 5 min, used with Flor Blanca, Papa Madre, Flor de Arena, Gauyusa, Pasuchaca, Malva, Amor Seco, Berbena, Llanten, Cola de Caballo, Chumbiaura, Palo de China, Huaminga, Quinuajiro. Drink 3 cups daily or 1l per day for 1-3 months.<br>2. Standard seguro mixture, see below.	1. Diabetes, Liver, Prostate, Allergies, Menstrual regulation, Blood related illnesses, Inflammation of the kidneys, Inflammation of uterus, Inflammation of the Liver, Inflammation of the Bladder, Cysts, Inflammation of ovaries, Inflammation of the womb, Uterus, Complement blood pressure, Menstrual pain, Inflammation (general), Spiritual Flowering<br>2. Good Luck, Good business, Protection, Good fortune, Good health	VFCHL22, JULS50, EHCHL39, ISA114, ISA43, JULS234, GER121

Family/Genus/Species	Indigenous name	Plant part used	Admin.	Preparation	Use	Coll. #
Gaultheria erecta Vent.	Mullaca Mistura, Mullaca, Mullaca Real	Whole plant, fresh or dried	Oral	1l of water and add 10g of Mullaca. Include 10g of each of the following: Humanarripa, Escorceonera, Eucalyptus, Matico, Veronica, and others. Drink 1 cup 3 times a day for 1 month.	Bronchitis, Asthma	JULS288, JULS198
Gaultheria reticulata H.B.K.	Toromaique, Toro Maique, Toromaike, Maique, Maque Candela, Toro Maique Amarillo, Toro Maique Verde, Gavilan Maique Amarillo, Gavilan Maique Verde	1., 3., 4., 5. Whole plant, fresh<br>2. Leaves, dried	1., 5. Topical<br>2. Oral<br>3. Seguro<br>4. Incense	1. 20-30 minutes boil for 50g per 7L of water and mix with other Maiques (7 varieties), 10g each of: Mishia Blanca, Mishia Colambo, Mishia Galga, Mishia Morada, Mishia Roja, Mishia Rosada and Toro Maique. Recite a prayer. Bath, 3 times per week. Bathe the patient in the mixture while rubbing him/her with the herbs. Afterwards, rinse the patient in water, and allow him/her to air dry.<br>2. 3g ground Leaves per 1 cup water, take once a month in the morning before breakfast.<br>3. Add 10g of plant material with Hierba de la Plata, Hierba de la Fortuna, Hierba del Oro, Carpintero, Chupa Flor, Señorita, Condores, Trenza Dias, Agua Florida, Tabu Perfume, Lima juice, Agua Bendita, and sugar. Place all ingredients into 1 bottle.<br>4. Smoke the area using a right to left motion as needed.<br>5. Boil 1/2 a cup of water with 50g of plant material for 5 minutes. Apply to affected area, once a day until it heals.	1. Cold, Arthritis, Bronchitics, Rheumatic pain, Bone pain, Hallucinogen, Bad Air / Mal Aire, Wounds, Ulcers, Sores, Anything else that bothers the body<br>2. Purgative<br>3. Protecting the house, Protecting the patient<br>4. Protecting the house, Protecting the patient, Rituals<br>5. Wounds, Cysts, Wounds from Sorcery	EHCHL57, JULS259, RBU/PL293, EHCHL171, EHCHL51, GER81, GER241, GER57
**ERIOCAULACEAE**						
Paepalanthus ensifolius Kunth	Madriguera	Whole plant, fresh	Seguro	1/5 of plant per Seguro.	Make a business succesful, To have control of employees	ISA149(106a)
**ERYTHROXYLACEAE**						
Erythroxylon coca Lam.	Coca	Leaves, dried	Oral	Add 5g of the leaf with 1 cup of water. Boil the mixture for 3-4 minutes, then let it cool. Gargle 3 times a day for 2 days. Drink 1 cup before bed for 2-3 days. Alternatively wash and chew about 5g of Leaves at a time.	Cold, Cough, Inflammation of the throat, Induce child birth, Strength for woman during childbirth, Helping delivery of newborn, Alertness, Ritual practices	JULS144, GER201
**EUPHORBIACEAE**						
Acalypha mandonii Muell.-Arg.	Chilca Dulce	Whole plant, fresh or dried	Oral	10g of the plant and 1l of water and boil for 3 to 5 minutes. Drink 3 times a day for 2 weeks.	Liver Inflammation, Clean blood from toxins	RBU/PL294

Family/Genus/Species	Indigenous name	Plant part used	Admin.	Preparation	Use	Coll. #
Chamaesyce hypericifolia (L.) Millspaugh	Lecherita, Lechera	Whole plant, fresh	1., 2. Topical<br>2. Oral	1. Break the Stems of the plant so that the juice drips out. Put milky sap in eye. 2 drops, 3 times per day.<br>2. Boil 10g Lecherita with 1l water. Combine with Cola de Caballo, Amor Seco, Linaza, Chacur and with 5 Stems and 5 Leaves of Marrajudio. Drink 1 cup every other day for 4 days and/or wash with solution. A couple of times per day, when needed.	1. Cataracts, White in vision or eye<br>2. Inflammation (external), Promoting lactation in women after birth	JULS67, GER41
Croton draconoides Muell.-Arg.	Sangre de Grado	Latex, fresh or dried	1. Oral<br>2. Topical	1. Cut only during the rainy season. Cut the Bark and allow 5 drops of the blood-like liquid into half a glass (8oz.) of water. Patient should drink solution at room temperature. 3 times a day up to 2 months. Heals scars from inside out.<br>2. Cut the Bark and extract the Resin that comes out of the Bark. Apply as poultice. Use 3 drops that had turned into foam and rub on affected area. Once a day as needed.	1. Internal ulcers, Internal bleeding, Gastritis, Blood circulation, Ulcer, Scars from insect bites, After internal surgery<br>2. Wounds (external), Scars, Acne	JULS244, GER100
Croton lechleri Muell. Arg.	Sangre de Grado	Latex, fresh or dried	1. Oral<br>2. Topical	1. Cut only during the rainy season. Cut the Bark and allow 5 drops of the blood-like liquid into half a glass (8oz.) of water. Patient should drink solution at room temperature. 3 times a day up to 2 months. Heals scars from inside out.<br>2. Cut the Bark and extract the Resin that comes out of the Bark. Apply as poultice. Use 3 drops that had turned into foam and rub on affected area. Once a day as needed.	1. Internal ulcers, Internal bleeding, Gastritis, Blood circulation, Ulcer, Scars from insect bites, After internal surgery<br>2. Wounds (external), Scars, Acne	JULS244, GER100
Hura crepitans L.	Habilla	Seeds, dried	Oral	Grind 3 Seeds, mix with 1 cup of oats. Make a hot cereal and drink warm1 cup once. Drink cold. Do not exceed the dosage, is very strong and can kill you.	Laxative, Removing laziness	JULS161, GER205
Jatropa curcas L.	Piñones	Seeds, dried	Oral	Grind 7 Seeds and mix with 1 cup of oats oats. Drink warm. Patient will react with diarrhea and vomiting. After patient's reaction, give strong tea. 1 cup, once.	Laxative, Getting rid of laziness	JULS231
Jatropa gosypiifolia L.	Piñones	Seeds, dried	Oral	Grind 7 Seeds and mix with 1 cup of oats oats. Drink warm. Patient will react with diarrhea and vomiting. After patient's reaction, give strong tea. 1 cup, once.	Laxative, Getting rid of laziness	JULS231
Jatropa multifida L.	Piñones	Seeds, dried	Oral	Grind 7 Seeds and mix with 1 cup of oats oats. Drink warm. Patient will react with diarrhea and vomiting. After patient's reaction, give strong tea. 1 cup, once.	Laxative, Getting rid of laziness	JULS231
Manhiot esculenta Crantz	Yuca	Tuber, fresh	1. Oral<br>2. Topical	1. Boil 1 cup of water and add 50g of Yuca and boil for 5 minutes. Drink cold 1/4 cup every day for 15 days.<br>2. Crush Peel of the tuber and remove extract with a piece of cloth. Rub affected area with extract and leave on for 3 hours. After it is dried, bathe. 2 times a day for 20 days.	1. Vaginal infection, Vaginal discharge<br>2. Allergies, Rashes	GER192

Family/Genus/Species	Indigenous name	Plant part used	Admin.	Preparation	Use	Coll. #
Phyllanthus niruri L.	Chanca Piedra	Whole plant, fresh or dried	Oral	10g of the plant, Cola de Caballo, Llantén, Boldo, Flor de Overo, Caña-caña, Flor Blanca and Flor de Arena and 1l of water and boil for 3 to 5 minutes. Drink 3 times a day for 2 weeks.	Liver Inflammation, Clean blood from toxins, Inflammation, Bladderstones, Liver, Kidneys, Blood, Inflammation of the gall bladder	JULS133, EHCHL167, GER152
Phyllanthus stipulatus (Raf.) Webster	Chanca Piedra	Whole plant, fresh or dried	Oral	10g of the plant, Cola de Caballo, Llantén, Boldo, Flor de Overo, Caña-caña, Flor Blanca and Flor de Arena, and 1l of water and boil for 3 to 5 minutes. Drink 3 times a day for 2 weeks.	Liver Inflammation, Clean blood from toxins, Inflammation, Bladderstones, Liver, Kidneys, Blood, Inflammation of the gall bladder	JULS133, EHCHL167, GER152
Phyllanthus urinaria L.	Chanca Piedra	Whole plant, fresh or dried	Oral	10g of the plant, Cola de Caballo, Llantén, Boldo, Flor de Overo, Caña-caña, Flor Blanca and Flor de Arena. and 1l of water and boil for 3 to 5 minutes. Drink 3 times a day for 2 weeks.	Liver Inflammation, Clean blood from toxins, Inflammation, Bladderstones, Liver, Kidneys, Blood, Inflammation of the gall bladder	JULS133, EHCHL167, GER152
Ricinus communis L.	Higrillo, Higrilla, Piñon, Higuerilla, Llonque	1. Leaves, fresh<br>2. Seeds, fresh	1., 2. Topical	1. Put oil on the leaf, then warm leaf and Flowers over the fire. Poultice, place on the stomach and wrap in plastic. Apply hot or lukewarm. Do not ingest. 2 times per day.<br>2. For pimples and ulcers grind 10g of Seeds with 1/2 glass of Cañazo (Llonque) and 5g of ground glass. Place mixture on top of the affected area. Leave it on for 2 hours. One time only.	1. Constipation<br>2. Ulcers (external), Pimples, Wounds	JULS83, GER19
FABACEAE						
Acacia macracantha H. & B. ex Willd.	Faique, Espino, Huarango	Bark, dried	Topical	1. Burn Bark and get ashes and strain. Place ashes on afected area. Cover entire wound. Once a day until the wound is healed.<br>2. Extract Resin from 20g of Huarango. Disolve Resin with animal (snake, mule, chicken, guinea pig) fat. A small amount on the affected area enough for a masage as needed.	1. Wounds, Stops bleeding<br>2. Arthritis, Rheumatism	GER28, JULS172
Caesalpinia paipai R. & P.	Pay Pay	Fruits, fresh or dried	Topical	Boil 10 Fruits in 1l of water for 30 minutes. Wash hair with cold tizana. Once a day in the AM for 3 days. Alternatively apply 1 time per day until wound heals. Amount applied depends on the size of the wound.	Killing lice, Wounds	GER40

Family/Genus/Species	Indigenous name	Plant part used	Admin.	Preparation	Use	Coll. #
Caesalpinia spinosa (Molina) Kuntze	Tara, Talla, Chanchalagua	Seeds pods, fresh or dried	1. Oral<br>2. Topical	1. Boil 3 pods with 1 cup water, mix with Romero, Coca, Frenegrew, Sangre de Grado and Bully Vinegar. Gargle Three times per day morning and night, gargle and spit out first, then drink 1 glass. Gargle and drink 1 cup in the morning, one at night, 6-7 months. Powdered Seeds can be applied directly to wounds.<br>2. 5g per 3l water. boiled for 20 minutes. Mixed with Laurel, Hierba del Susto, Ajenco, and Ishpingo. Bath, 1 time per week, wash wounds three times per day for 1-3 months. Can be also used as a vaginal wash or sitting bath, 2 times a day for 2 days.	1. Pharyngitis, Throat, Skin infection, Animal bites, Antibiotic, Inflammation of the tonsils<br>2. Fungus, Skin infection, Angina pectoris and similar conditions, Antibiotic, Wounds, Cleaning wounds, Boils, Animal bites, Amoeba infections, Inflammation of ovaries, Inflammation of uterus, Inflammation of the vagina	ISA55, EHCHL27, VFCHL21, JULS255, GER143
Cajanus cajan (L.) Millsp.	Chivato	Whole plant, fresh or dried	Topical	Boil 3l of water with 10g total of Eucalyptus, Manzanilla, Ilambo, Cordon de Muerto, and Chivato, boil for 5 minutes. Bathe with the water and rub with the plants. 2 times a week or 4 times a month as needed, depending on how bad the Mal Aire was.	Bad Air / Mal Aire	JULS136
Cassia fistula L.	Caña Fistula	Seeds, fresh or dried	Oral	Boil 10g per 1l water. Drink 1 cup daily as needed (approximately 15-20 days).	Nervous system, Epilepsy	RBU/PL386
Cicer arietinum L.	Garbanzo	Seeds, dried	Oral	Boil 1kg of Garbanzo in 1l of water for 5 minutes. Drink lukewarm, 1 cup a day for 15 days.	Cancer	GER46
Desmodium molliculum (H.B.K.) DC.	Pie de Perro, Pata-Perro, Pata de Perro, Chancas de Comida, Muña, Manayupa	Whole plant, fresh or dried	1. Oral<br>2. Topical	1. Boil 10g Pie de Perro per 1l water. Combine with Chacur, Amalba, Amor Seco, and Verbena. Drink 4 times per day for one month.<br>2. Boiled 10 minutes, 20g per 1l water with Llantén, Matico. Wash once per day.	1. Inflammation (internal), Inflammation (external), Inflammation of the kidneys, Diarrhea, Stomachache, Kidneys, Inflammation of the ovaries, Inflammation of the womb, Gastritis<br>2. Wounds (cleansing), Scars	JULS41, RBU/PL268, GER135, JULS44, EHCHL109, RBU/PL256
Desmodium triflorum (L.) DC	Pega Pega	Whole plant, fresh	1. Seguro<br>2. Topical	1. Standard Seguro mixture, see below.<br>2. Standard mixture for Spiritual Flowering, see below.	1. Spiritual Flowering<br>2. Good business, Protection, Good fortune, Good health	GER122, RBU/PL347
Diodea virgata (Rich.) Amsh.	Yin Yin	Whole plant, fresh	1., 2. Oral	1. Serve the whole plant. Take 130g of plant everyday.<br>2. Remove sSeds from the pod. Pop the Seeds into a child's mouth, then promptly instruct the child to close their mouth. Repeat 3 times. Repeat this procedure 2 times a day, for 3 days.	1. Fertility of Cuy<br>2. Promoting child speech	JULS270

Family/Genus/Species	Indigenous name	Plant part used	Admin.	Preparation	Use	Coll. #
Dolichos lablab L.	Frijol chileno	Fruits, fresh	Oral	Boil for 10 minutes 1/2kg of the plant material in 1l of water. Drink it at room temperature. 1/2 cup 2 times a day for 8 days.	Fever, Inflammation of the intestine, Protects the lungs	GER235
Erythrina ormosia	Huariro, Huairuro, Huayruro	Seeds, dried	Charm	Make a bracelet with the Seeds. Wear at all times on the left hand	Evil Eye / Mal Ojo	JULS170
Erythrina velutina Willdenow	Porotillo	Flowers, Leaves and Stems, fresh or dried	Topical	5g per 1l water. Adults: add 2 Tbsp Glycerine oil, Children: add 1 Tbsp Glycerine oil. 1 enema every 3 months or 1 every 6 months depending on condition.	Cleansing of the intestine	ISA75
Erythrina spp.	Huailulo, Huairuro	Seeds, dried	Charm	Make a necklace with the Seeds and have the shaman bless it with spiritual prayers. Wear the necklace, made for life.	Protection from evil	GER196
Indigofera suffruticosa Miller	Añil	Stems, fresh	Oral	Boil 20g of Anil in 1 cup of water for 5 minutes. Drink lukewarm, 1/2 cup 1 time only.	Cleaning of the woman, Expelling placenta from woman after giving birth	GER198
Inga edulis C. Martius	Huaba, Pacae, Guava, Pacai	1. Seeds, fresh or dried<br>2. Flowers, fresh	1. Oral<br>2. Topical	1. Grind 10 Seeds,. 10g of Juan Alonso and Alcaparilla and boil in 1/2 cup of water for 2 minutes, then mix with 1/2 glass of orange juice. Drink 3 - 4 times a day for 2 months or as needed.<br>2. Add 15g of the Flowers with 1l of water. Boil the mixture for 3 minutes, then let it sit. After shampooing, apply the mixture to the patient's hair without rinsing.	1. Rehabilitation of drug addicts or alcoholics, Laxative<br>2. Adding shine and beauty to hair, Hair growth	JULS168, JULS304, GER17
Inga feuillei DC.	Huaba, Pacae, Guava, Pacai	1. Seeds, fresh or dried<br>2. Flowers, fresh	1. Oral<br>2. Topical	1. Grind 10 Seeds, 10g of Juan Alonso and Alcaparilla and boil in 1/2 cup of water for 2 minutes, then mix with 1/2 glass of orange juice. Drink 3 - 4 times a day for 2 months or as needed.<br>2. Add 15g of the Flowers with 1l of water. Boil the mixture for 3 minutes, then let it sit. After shampooing, apply the mixture to the patient's hair without rinsing.	1. Rehabilitation of drug addicts or alcoholics, Laxative<br>2. Adding shine and beauty to hair, Hair growth	JULS168, JULS304, GER17
Lathyrus odoratus L.	Tacon Blanco, Pensamiento Blanco	Flowers, Leaves and Stems, fresh or dried	Oral	5g per 1l, 3 min, combined with Toronjil, Pimpinela, Mejorana, and Cedron. Drink 1l per day or 3-4 cups a day for one month.	Heart, Nerves, Anxiety	VFCHL43
Lens culinaris Medikus	Lenteja	Seeds, dried	Oral	Boil 1l of water with 200g of lentils for 5 minutes. Drink cold, 2 times a day for 20 days. Eat lentils with rice.	Protect bones	GER233
Leucaena leucocephala (Lam.) De Wit	Arabisca, Huaba Bruja	Bark, Flowers and Stems, fresh	Topical	Boil 1l of water for 3-4 minutes with 10g of Arabisca, Verbena, Hierba Santa, Llanten, Cola de Caballo and Chacur. Wash the wound, herpes and/or rashes 2 times a day or as needed until the wound is dried.	Antiseptic, Clean wounds	JULS104

Family/Genus/Species	Indigenous name	Plant part used	Admin.	Preparation	Use	Coll. #
Lupinus mutabilis Sweet	Chocho, Tarhui	Seeds, dried	Oral	Soak plant material in water for 1 week, then either puree the material with onion and cook for 2 minutes, or make a salad with the soaked material. Oral as a salad or as a puree. Oral 1 plate a day, as necesary.	Malnutrition, Nutritional supplement	JULS254
Medicago sativa L.	Alfalfa	1. Flowers and Leaves, fresh<br>2. Flowers, fresh	Oral	1. Blend Leaves and Flowers with water. Drain, and obtain extract. Drink extract. Honey can be added, if desired. Take 1 glass of extract, twice a day.<br>2. Blend 20 Flowers in 1/4 cup of water. 1 small cup 1 a month for 3 months. Do not exceed dosage or might loose eyesight. Plant is very hot.	1. Bronchitis<br>2. Mouth bitternes, Kidney disease	JULS96, GER42
Melilotus alba Medikus	Alfalfilla	Seeds, dried	Oral	1. Boil for 10 minutes 10g of grinded Seeds in 1/2l of water. Drink lukewarm. 1/4 cup a day for 15 days.<br>2. Boil for 10 minutes 100g of the plant material in 1/2l of water. Drink cold, 1/2 a cup. Once a day for 8 days.	1. Gain weight<br>2. Fever, Tuberculosis, Colds, Infections internal, Respiratory infections	GER223
Mimosa albida H. & B.	Tapa Tapa					JULS
Mimosa nothacacia Barneby	Uña de Gato de la Costa	Bark, dried	1. Oral<br>2. Topical	1. 10g of the Bark in 1l of water, boill for 3-4 minutes. Drink cool, a cup 3 to 4 times a day as needed.<br>2. Boil 200g of Uña de Gato de la Costa into 3l of water for 10 minutes and then place hot water into a tub. Pour hot tizana into a tub and then sit in it for about 5 minutes. 2 times a week until the patient is cured.	1. Cancer, Kidney inflammation, Hepatitis, Hemorrhoids, Liver Inflammation<br>2. Anus cyst, Vaginal pimples, Anal pimples	JULS265, GER199
Myroxylon balsamum (L.) Harms.	Quina Quina, Kina Kina	Seeds, dried	1., 3. Oral<br>2. Topical	1. Grind 20 Seeds, mixed with Seeds from a specific seven other plants: Ashango, Pucho, Amala, Ishpingo, Mozcada, Cabalonga and put in a bottle of wine and and amacerar for 8 days. Drink 3 small cups per day.<br>2. Boil 20 Seeds per 5l water for 20-30 min with Ishpingo, Ashango, Pucho, Amala, Raucho, Tokio, Nuez Moscada, Pepa de Cedron (use only the Seeds of these herbs) with 1l of 90 proof alcohol and add 2 pieces of tobacco, 2 pieces of Ajo Macho, 10g of Quina Quina, 2 Leaves of Pacra, 1 branch of both Eucalyptus and Maye. Do not leave bath outside, take bath every other day. 3 times per week.<br>3. 3 Seeds, toasted and crushed, per 1 cup of water. Drink 1/2 cup for adults, 1 tsp for children.	1. Nervous system, Bad Air / Mal Aire, Epilepsy, Bronchitis<br>2. Bad Air / Mal Aire, Bronchitis, Fright / Susto, Headache<br>3. Cough, Bronchitis, Asthma	JULS287, RBU/PL382, EHCHL151, VFCHL46, GER91

Family/Genus/Species	Indigenous name	Plant part used	Admin.	Preparation	Use	Coll. #
Pisum sativum L.	Arberjas, Arvejas	Seeds, fresh	Oral	100g and 1 cup of water and boil. Drink warm, 1 cup 1 to 2 times a day for only 1 day. Patient should drink cold solution. 1/2 cup only 1 time.	Smallpox, Rubiola, To promote the release of all bad and it's coming out, Inflammation of the intestine	JULS105, GER211
Prosopis pallida (H. & B. ex Willd.) H.B.K.	Algarrobo	1. Seeds, dried<br>2. Leaves and Stems, fresh<br>3., 4. Resin, fresh	1., 2. Oral<br>3., 4., 5. Topical	1. Boil 10kg of Algarrobo Fruit and Seeds for 3 hours in medium to high heat until thickened. Turn off fire and let sit until cool, then drain and place syrup in bottle. Drink 2 Tbsp per small cup, 3 times per day as long as you wish.<br>2. Boil 5g of Algarrobo Bark in 1/4 cup of water for 3 minutes. Inside the Bark is a spring, so the Bark has to be cut in half to take it out.<br>3. With a knife extract the Resin exposed on the trunk. Place 5g of Resin in a pan to warm. then use. Masage the affected area with Resin. Once a week for 3 weeks.<br>4. Place a couple drops on top of tooth cavity. The Algarrobo Resin will pulverize the tooth. Patient should be very careful while applying because it will destroy all tooth touched by the Resin.<br>5. Grind 100g of Algarrobo charcoal, 100g of sulphur and 100g of garlic. Apply on affected area. Once a day until healed.	1. Cough, Anemia, Fertility, Sexual potency, Bronchitis, Nutritional Supplement<br>2. Stomachache, Hangover<br>3. Arthritis, Rheumatism, Colds, Bone ache<br>4. Toothache, Pull out tooth<br>5. Critical wounds	JULS97, GER8
Senna bicapsularis (L.) Roxburgh	Alcaparrilla, Alpacaquilla	Whole plant, fresh	Topical	Add 10g of Alcaparrilla, with 1l water. Also add 40g of a mixture consisting of Cola de Caballo, Alonso Verbena. Boil the mixture for 3-5 minutes. Drink 1 cup, 3-4 times a day for one month, or as needed.	Detoxification of alcohol and drugs, Detoxification of liver and kidneys	JULS95
Senna monilifera H.S. Irwin & Bowley	Hojas de Sen	Leaves, dried	Oral	3g per glass of water for children. 5g per glass of water for adults. One time per month.	Purgative, Constipation, Cleansing of the stomach	EHCHL34, EHCHL12
Senna occidentalis (L.) Link.	Retania, Retana	Whole plant, fresh or dried	Oral	1l of water with 10g total of Retania, Amor Seco, Cola de Caballo, Linaza, Chacur, Pie de Perro. Drink warm, 1 cup 4 times a day for 1 month.	Inflammation (general)	JULS152
Spartium junceum L.	Retama	1. Flowers and Root, fresh<br>2. Whole plant, fresh	1. Oral<br>2. Topical	1. 3-5g per 1l of water, combined with Flores de Overo. 3 times a day.<br>2. Boiled for 20 minutes and mixed with Maiques. Bath, 3 times per week for one week. 50g boiled 20-30 minutes, per 5l of water, with herbs of luck, herbs of strength (like Condor and Trensilla) as steam bath, 3 times per week.	1. Hepatitis, Liver, High blood pressure, Diabetes<br>2. Arthritis, Good Luck, Bone pain, Sinusitis, Blood purification	EHCHL60, EHCHL 146, RBU/PL279, JULS239

Family/Genus/Species	Indigenous name	Plant part used	Admin.	Preparation	Use	Coll. #
Tamarindus indica L.	Tamarindo	Fruit pulp, fresh	Oral	Remove the pulp from 250g of plant material. Add this to 3 glasses of warm water. Blend the mixture. Drink the mixture cold while fasting. One glass in the morning Once a day for 30 days. Repeat as necessary.	Laxative, Blood circulation, Epilepsy, Heart disease	JULS252, GER219
Trifolium repens L.	Trebol, Trebol de agua	Flowers, Leaves and Stems, fresh or dried	Oral	1 Tbsp per 3l water. Can combine with Lancetilla, Colores. Can also sometimes combine with a little Zarzaparilla. Drink 1l daily, 1-2 months or take 1 Tbsp of the freshly chopped plant in the morning every day, 1 week.	Inflammation, Inflammation of the urinary tract, Stomach, Stomach Pain, Ulcer, Inflammation of the kidneys, Kidneys, Blood	ISA47, RBU/PL330, EHCHL30
Zornia reticulata Sm.	Hierba de la Vibora	Whole plant, dried	Oral	5g per 1l water with Conchalagua, Norbo, Colores, three times per day during meals.	Nervousness	EHCHL122
GENTIANACEAE						
Coutoubea ramosa Aublet	Genciana	Fruits, fresh	Topical	Squeeze the juice out of the Fruit, squeeze as much as you need at a time. Apply a couple of drops of the fresh squeezed juice on the affected area and let it air dry. A couple of drops 1 time a day as long as needed.	Wounds, Scars, Rashes, Cold sores	GER207
Gentianella bicolor (Wedd.) J. Pringle	Corpus Way, Corposhuar, Hornamo Leon	Whole plant, fresh or dried	1. Oral<br>2. Seguro	1. Boiled 2-3 minutes, 1l daily as needed. Tea is very bitter.<br>2. Mix plant material with Hierba de la Plata, Hierba de la Fortuna, Hierba del Dollar, Hierba de la Justicia, Hierba del oro, Carpintero, Señorita, Sonrisa, etc. Place all the herbs in one bottle with Agua Florida, Lima juice, etc. Use mixture for rituals, or use as a seguro.	1. Arthritis, Diabetes, Bone pain, Cholesterol, Gastritis, Liver, Blood, Rheumatism<br>2. Good luck, Good health, Good business, Protection	EHCHL14, VFCHL5, RBU/PL304, JULS167
Gentianella bruneotricha (Gilg.) J.S. Pringle.	Anga Macha	Whole plant, fresh	Oral	1/2l of water with 5g of Valeriana Estrella and 5g of Anga Macha. Let boil for 3 minutes. Drink hot.1 glass 3 times a day for 2 to 3 days.	Infection of the uterus, After giving birth	JULS282
Gentianella crassicaulis J.S. Pringle	Violeta Genciana	Whole plant, fresh or dried	Oral	Boil 30g per 1l water, 3-5 min. Combine with Pasuchaca, Amargon, Corpus Way. 3-4 glasses per day for 15-30 days.	Gastritis, Diabetes (special types), Dizziness	VFCHL7
Gentianella dianthoides (H.B.K.) Fabris	Genciana, Egenciana, Amargon, Campanilla	Whole plant, fresh	Oral	Boil 10g Genciana with 1l water for 2 minutes. 1 time per day, take before eating, in the evening, every other day, for 1 week. Overdosing can cause miscarriage in pregnant women. The plant contains cortizone.	Liver, Kidneys, Blood, Purgative to loosen the stomach, Diabetes, Cleansing, Blood irrigation, Blood problems, Liver Infection	RBU/PL253, RBU/PL320, JULS56, TRUVan/Erica21, EHCHL136, EHCHL61

Family/Genus/Species	Indigenous name	Plant part used	Admin.	Preparation	Use	Coll. #
Gentianella graminea (H.B.K.) Fabris	Sumaran, Chinchimali, Corpushuay	Whole plant, fresh or dried	Oral	20g per 1l water. 1l daily, 1 week, best with food, because it has a bitter taste. Drink cool while the patient is fasting. Exceeding doseage can lead to blindness.	Diabetes, Liver, Blood, Burn fat, Intestinal fever, Cough, Fever, Infection, Allergies of the Blood, Varicose veins, Blood purification, Inflammation of the liver, Blood detoxification	EHCHL22, RBU/PL285, VFCHL8, JULS148
GERANIACEAE						
Erodium cicutarium (L.) L'Herit.	Agujilla Blanca, Auguilla, Augilla	Whole plant, fresh	Oral	Boil 1 Tbsp Sap per 1l of water, mixed with Ambarindas, Hierba del Toro and Sanguinaria.r. 1l per day, 1-3 months.	Inflammation, Bronchitis, High blood pressure, Low blood pressure	ISA110, ISA54
Geranium ayavacense Willd ex H.B.K.	Puli Punchi, Pasuchaca, Pachuchaca, Miscamisca	Whole plant, fresh or dried	1., 2. Oral	1. Boil 1l water 3 minutes, then add 10g Pasuchaca and 1/2 leaf of Nogal. Combine with Culein and Citrodora. 4 cups per day for life.<br>2. Boil 1l water with 10g Pasuchaca for 3 minutes. Combine with Chacur, Cola de Caballo, Verbena, Unquia, Amor Seco, and Grama Dulce. 4 cups per day for 1 month.	1. Diabetes<br>2. Inflammation, Kidneys, Liver, Urinary tract, Inflammation of all kinds	JULS48, EHCHL63, VFCHL6
Geranium sesiliflorum Cavanilles	Puli Punchi, Pasuchaca, Pachuchaca, Miscamisca	Whole plant, fresh or dried	1., 2. Oral	1. Boil 1l water 3 minutes, then add 10g Pasuchaca and 1/2 leaf of Nogal. Combine with Culein and Citrodora. 4 cups per day for life.<br>2. Boil 1l water with 10g Pasuchaca for 3 minutes. Combine with Chacur, Cola de Caballo, Verbena, Unquia, Amor Seco, and Grama Dulce. 4 cups per day for 1 month.	1. Diabetes<br>2. Inflammation, Kidneys, Liver, Urinary tract, Inflammation of all kinds	JULS48, EHCHL63 VFCHL6
Pelargonium odoratisimum (L.) L'Herit.	Malva de Oro, Malva de Olor, Malva Olorosa	Whole plant, fresh or dried	1. Oral<br>2. Topical	1. 5g per 1l boiling water. Drink 1l per day.<br>2. Boil 2l of water with 10g of Ishpingo, Eucalyptus, Cordon de Muerto, Flor de Chocho, and Flor de Retama. Boil for 3 minutes. Bathe 2-3 times a month.	1. Arthritis, Heart, Nerves, Blood, Mal de susto, Inflammation of the ovaries, Inflammation of the womb<br>2. Fright / Susto	TRUVan/Erica14, TRUBH6, EHCHL89, JULS188
Pelargonium roseum Willd.	Geranio	Flowers and Leaves, fresh	Oral	Boil 10g Geranio with 1l water. Drink 3 times per day, as needed. Solution can also be used to gargle, 3-4 times daily for 3-4 days.	Hemorrhages, Uterus pain, Inflammation of the uterus, Tonsilitis, Infection of the throat	JULS84
HIPPOCRATEACEAE						
Tontelea crassifolia (Mart.) Spreng.	Bejuco de Montaña	Seeds and Stems, fresh or dried	Topical	200g per 3l boiling water, take 2 baths per month.	Nervous system	RBU/PL383

Family/Genus/Species	Indigenous name	Plant part used	Admin.	Preparation	Use	Coll. #
ILLICIACEAE						
Illicium verum Hook. f.	Anis Estrella	Seeds, dried	Oral	Take 10-15g of plant material, add it to 1l water, and boil for 2-3 minutes. Drink warm. Serve in a baby bottle, 3-4 times a day, for 1-2 weeks.	Expel residues of feces in stomach of newborn babies	JULS102
ISOETACEAE						
Isoetes andina R. & P.	Piri Piri	Stems, fresh	Oral	2 small branches in cup of boiling water. Drink 1 cup per night for 1 month.	Male impotence	ISA100
JUGLANDACEAE						
Juglans neotropica Diels	Nogal	Leaves, fresh	1., 2., 5. Topical<br>3., 4. Oral	1. 20g per 1l of water, boil 20 for min. masage head for 3 minutes, 3 times per week.<br>2. 3l water with a bundle or pouch of the herb, 3 times per month.<br>3. 10g per 1l, boil water for 3-5 min. For Bronchitis: mix with Matico, Enredadera, Borraja. 3 glasses per day, 1l daily.<br>4. 1 Tbsp with the Pasuchaca, 1 L daily.<br>5. Limpia, mixed with Añasquero Grande, Rumilanche, Ishpinguillo, Sauco, 3 per month.	1. Hair loss<br>2. Sorcery, Daño, Arthritis, Wounds (cleansing), Fright / Susto<br>3. Cough, Bronchitis, Asthma<br>4. Diabetes<br>5. Sorcery, Daño, Arthritis, Wounds (cleansing), Fright / Susto	RBU/PL273, ISA67, EHCHL4, ISA123
KRAMERIACEAE						
Krameria lappacea (Dombey) Berdet & B. Simpson	Ratania, Raima	Leaves and Root, fresh	Oral	10g per 1l water, boil 3 min, drink as needed.	Inflammation of the kidneys, Inflammation of the ovaries, Inflammation of the intestine, Internal Inflammation, Inflammation of the bladder	JULS53
LAMIACEAE						
Hyptis sidifolia (L´Her.) Briq.	Pedorera, Pedrorera, Hierba de la Ventosedad, Pedorrera, Albaca Serrana	Whole plant, fresh or dried	Oral	5g per 1l boiling water for 3-5 minutes. Take 1 cup, three-four times a day for 2-3 days as needed. When stomach is hard this plant looses up stomach and gases are released both ways.	Gases, Colic of the intestine, Gastritis, Cramps	EHCHL21, RBU/PL254, JULS222, JULS4, GER76
Lavandula angustifolia Miller	Alucema, Alhucema, Labanda	Flowers, Leaves, Stems and Seeds, dried	1. Oral<br>2. Topical	1. Do not use roots. Boil 1l of water, then add a total of 10g of Labanda, Romero, Claveles, Hinojo, Toronjil, Anjenjo, Manzanilla, and Pinpinela for 2 minutes. Patient should drink lukewarm solution. 1 cup 3 to 4 times a day for 1 month.<br>2. Boil 3l of water with 20g total of Labanda, Romero, and Eucalyptus for 3 minutes. Patient should wash with water and masage with Flowers. 2 times a week or 4 times a month or as needed.	1. Cold, Gases, Heart, Nerves<br>2. Relaxant, Stres	GER113, JULS177

Family/Genus/Species	Indigenous name	Plant part used	Admin.	Preparation	Use	Coll. #
Lepechinia meyenii (Walpers) Epling	Salvia, Salvia Real	Whole plant, fresh or dried	1. Oral<br>2., 3. Topical	1. Boil 30g per 1l water. Take with meals, three times per day.<br>2. 100g per 8L for 5 minutes, combined with Romero, Llantén. Bathe three times per day for one month.<br>3. 5g per 3l water, mixed with Añasquero Chico, Ajenco, Nogal and Bully Vinegar. Bath, 2 times per month.	1. Bronchitis, Heart, Nerves, Memory, Menstruation<br>2. Wounds, Hair loss<br>3. Fright / Susto, Freight / Susto in children	RBU/PL303, VFCHL17, ISA91
Marrubium vulgare L.	Cordon de Muerto, Chanca de Comida, Chancaz de Muerto	Whole plant, fresh or dried	Topical	Boil 20 to 30g of Cordon de Muerto, mix with Eucalyptus, Ishpingo, Chiuato, Quinual and place in 2 to 3l of water for 5 to 8 minutes. Empty into a bucket and place in an enclosed room. Rub the solution on your whole body with all the plants.2 baths a week for 4 to 5 months if the patient is in good condition. Do not let the solution touch the mouth.	Mal de susto, Inflammation of the body	JULS132
Melissa officinalis L.	Toronjil, Melissa	Whole plant, fresh or dried	1. Topical<br>2. Oral	1. Boil in 2l water for 10 minutes. Mixed with Torongil, and Churguis. Bathe twice a week, or as needed.<br>2. 20-30g per 1l water, 2 min, with Pimpinela, Cedron, Mejorana, Siempre Viva, flores de Amelas, Romero, Claveles, ongona, Manzanilla, Mejorana, Pimpinela, Naranja Flowers. Drink 4 cups per day, 1 month.	1. Ill-mannered children<br>2. Pain of love, Nerves, Insomnia, Heart, Nervous system, Tachycardia	JULS26, EHCHL2, RBU/PL260, VFCHL14
Mentha x piperita L.	Poleo	Whole plant, fresh or dried	Oral	Boil 1l, then add 10g Poleo. Take when symptoms occur.	Colic, Stomach Pain	JULS29
Mentha spicata L.	Hierba Buena, Hierba Buena Silvestre, Menta	Whole plant, fresh	1. Oral<br>2. Topical	1. 10g in 1l of boiling water. Can be mixed with Anis. Drink as needed, 1-2 times per day in the morning and afternoon or after each meal, 3 times per day, 1 month.<br>2. Boiled 20 minutes, 30g per 6l water, with other herbs of luck. Bathe 3 times per week.	1. Parasites, Colic, Stomach ache, Gastritis, Indigestion, Colic of the stomach, Tapeworms, Intestinal worms, Headache, Aphrodisiac, Gases, Bad breath<br>2. Colic, Stomach ache	RBU/PL308, EHCHL74, RBU/PL267, JULS72, VFCHL3, JULS20, GER15, GER134, JULS20
Minthostachys mollis Griesebach	Muña, Chancas de Comida	Leaves and Stems, fresh	Oral	2-10g per 1l of water, drink 1 cup 3-4 times per day, for 3-4 weeks. Patient should drink hot solution. Can also be eaten as a vegetable or in soup.	Colic, Gases, Parasites in the stomach, Stomach ache, Heart, Nerves, Diarrhea	EHCHL84, JULS200

Family/Genus/Species	Indigenous name	Plant part used	Admin.	Preparation	Use	Coll. #
Ocimum basilicum L.	Albaca Mistura, Albaca Negra, Albaca, Albaca Morada, Albahaca (costa)	Whole plant, fresh	1., 5., 6. Topical 2., 3., 4. Oral	1. Use 20g of herb mixed with Agua Florida, Eucalipto, Alcanfor, Molle, and cane alcohol, Agua del Susto, Flor de Retama, Hierba del Gallinazo. Mix with Ruda Hembra and Macho, Ajenjo, two peppers. Limpia, take it on Tuesday, Friday, and next Tuesday, 3 times.<br>2. 10g per 1 cup with a little salt. Drink one cup very hot. Drink also immediately after giving birth.<br>3. 5g per 1l boiling water.1 cup per day, 2 weeks.<br>4. 20g per 1l for 1-2 minutes combined with Tilo, Toronjil, Mejorana, Cedron. Three cups per day for 10 days, before dinner.<br>5. 100g per 1l for 5-8 minutes combined with Romero, Salvia Real, Yerba Santa, Malva, Olorosa. Bathe three days per week or every eight days.<br>6. Place 1 Seeds directly into the eye. Leave there for 3 hours or until the eye waters it out. 1 time a month for only 2 months.	1., 4., 5. Daño, Fright / Susto, Bad Air / Mal Aire, Insomnia, Low blood pressure, Good luck<br>2. To promote dialation of the uterus, Hasten delivery, Colic, Gases, Preventing infections related to birth, Refreshing womb, Reducing inflammation after birth<br>3. After birth<br>6. Cataracts, Eye problems	JULS54, EHCHL48, VFCHL13, RBU/PL284, TRUVan/Erica8, GER191
Origanum majorana L.	Mejorana	Leaves and Stems, fresh	Oral	10g Mejorana per 1l water. Combine with Sanguinaria, Congona, Toronjil, Melisa, Manzanilla, Hinojo, Albahaca Serrana, Poleo, Manzanilla and Vaniour. Patient should drink warm solution. 4 cups per day for 1 month. For diabetes 4 times a day for life.	Colic, Heart, Nerves, Menstration, Anxiety, Depression, Pain of love	EHCHL88, JULS19, RBU/PL317, GER165
Origanum vulgare L.	Oregano	Leaves and Stems, fresh or dried	Oral	Add 1 cup of water with 3g of Oregano. Boil for 3-5 minutes. Drink hot for menstrual periods. Drink warm for colics. Avoid drinking excesive amounts because it may cause abortion. Take 1 cup, 2 times a day for 1 day.	Colic, Menstrual cramps, Menstration, Stomach ache, Gases, Lower stomach cramps related to PMS	JULS205, GER114
Otholobium glandulosum (L.) Grimes	Culein, Culen	Stems, fresh or dried	Oral	5g per 1l of water. Combine with Manzanilla, Menta, and Anis. Drink 3 times per day. Patient should drink warm solution.	Diarrhea, Cold of the stomach, Diabetes	EHCHL5, JULS40

Family/Genus/Species	Indigenous name	Plant part used	Admin.	Preparation	Use	Coll. #
Rosmarinus officinalis L.	Romero, Romero Castilla	Leaves, fresh or dried	1., 3., 4. Topical<br>2. Oral	1. Boil Romero Otomillo with Nogal, Amor Seco, and Cola de Caballo. Massage scalp with some of the prepared tizana, then apply shampoo and wash like normal.<br>2. 5g with Eucaliptus, Cola de Caballo and Ruda in 1l boiling water. 2 cups per day for 2 days. Take 4 cups per day for 1 month.<br>3. Boil 1 bundle of Romero Castilla with 3l water for 10 minutes. Combine with Llantén, Manzanilla Blanca, Savila, Palo Blanco, Manzanillón, the Flowers of Retama, the Flowers of Chochos, Cordon de Muerto, Verbena, Melisa, Eucalyptus and 7 Espiritus. Inhale or bathe Tuesday, Friday, and the following Tuesday for 10 minutes each time.<br>4. Burn 1 bundle of Romero Otomillo with several other plants, including Palo Santo. Cut the plant and put it on top of burning charcoal and inhale.	1. Hair loss<br>2. Gases, Heart, Nerves, Bronchitis, Indigestion, Colic, Headache, Stomach, Pain of love<br>3. Fright / Susto, Dispelling negative energy in the house<br>4. Purify, Absorb negative energy, To reduce humidity	RBU/PL329, ISA78, TRUBH11, EHCHL3, JULS27, VFCHL2, ISA105
Salvia ayavacensis H.B.K.	Ticra, Sticra	Leaves, fresh or dried	Topical	1 handful per 1l boiling water. Can combine with Tutapure Blanco, Lailambo, Conchalay Amarillo, Conchalay Blanco, Ticra, Zanahoria, Poleo de Gentil, and 7 Espiritus. 1-2 baths per month. Do not ingest.	Fungus all over the body, Daño, Fright / Susto, Sorcery, Skin inflammation	ISA37, ISA150(92a), RBU/PL290
Salvia cuspidata R. & P.	Salvia Blanca	Whole plant, fresh or dried	Topical	1 bundle boiled for 5 minutes with 3l water. 1 bath per week.	Fright / Susto, Daño	RBU/PL315
Salvia discolor H.B.K.	Palmeras (Chica), Llatama, Yatama	Stems, fresh	1. Seguro<br>2., 4. Oral<br>3. Topcial	1. 3 Stems per flask.<br>2. Three Leaves per cup. do not mix with other herbs. One cup a day for a week.<br>3. Used with de with Ishpinguillo, Hierba del Gallinazo, Ajos Giro, 7 Espiritus. Steam bath twice per month or bath 2-3 times per month for children.<br>4. 1 Tbsp boiled with 1 cup water. 1 cup daily, 1 month.	1. Success<br>2. Cough<br>3. Clean the energy of the home, Preventing infections related to birth, Fright / Susto in children<br>4. Preventing infections related to birth	ISA93, ISA151(93a), ISA25
Salvia macrophylla Benth.	Cuchichara	Leaves, fresh or dried	Topical	Toast and beat into a pulp. Put pulp on wound.	Wounds from Sorcery, Chronic gangrene	ISA29
Salvia officinalis L.	Salvia	Whole plant, fresh or dried	Oral	In 1l of water boil 10g of the plant for 3-5 min. It can be mixed with Matico, Nogal and Eucalyptus. Drink hot, 1 cup 3 to 4 times a day as needed. Up to one month.	Cough, Bronchitis, Control and regulate menstrual cycle	JULS241

Family/Genus/Species	Indigenous name	Plant part used	Admin.	Preparation	Use	Coll. #
Salvia rosmarinifolia Hort. ex G. Don.	Romero del Campo, Romero Blanco, Romero Serrano	Whole plant, fresh	1., 2., 3. Topical	1. Boiled 20 minutes, 20g per 1l water, with other Romeros, Salvia, Mejorana. Administer while tepid and absorb the vapors produced. Steam inhalation 2-3 times per month.<br>2. Alternatively burn one bundle with charcoal, Palo Santo, incense and Myrrh and Eucalyptus. Patient should surround themselves with the vapors and smoke produced.<br>3. Can also be used as bath: 1 bundle or pouch per 3l boiling water, with Canchalagua and Cola de Caballo. Fresh: use leaf and Stems. One time only (Tuesday or Friday).	1., 2., 3. Inflammation, Bad Air / Mal Aire, Negative energy, Cleansing of the home, Fright / Susto, Sinusitis	JULS49, ISA118, ISA77, EHCHL108, GER111
Salvia sagitatta R. & P.	Salvia Negra	Root and Stems, fresh or dried	Oral	10g per 1l water, drink 3 times per day, as needed	Cough, Asthma, Hair loss	RBU/PL318
Salvia tubiflora R. & P.	Hierba del Recaida, Hierba del Aire, Cutiquero, Yuca del Aire, Paja del Aire	Whole plant, fresh or dried	1. Oral<br>2. Topical	1. 5g per 1l water. Do not mix with other plants. 1 cup three times per day.<br>2. 500g per 1l alcohol, boiled for 20 minutes, with 1 bottle of Agua Florida, 1 bottle of Agua Cananga and finish filling the bottle with Cañazo. Bath, 3 times per week.	1. After birth, Bad Air / Mal Aire, Body pain<br>2. Bad Air / Mal Aire, Aneurism, Paralysis, Body pain, Half body paralyzed, Facial paralysis	EHCHL148, RBU/PL286, EHCHL49, GER70, GER175
Satureja pulchella (H.B.K.) Briquet	Panizara, Panisara	Leaves, fresh or dried	Oral	Add 50g of plant material with Culein, Manzanilla, Chancas de Comidas or Muña in 1/2 cup of water. Boil the mixture for 3 minutes. Drink the mixture cold. Take 1/8 cup once a day, for 3 days.	Bronchitis, Asthma, Liver disease, Infection (internal), Nerves, Menstrual delay, Providing vitamins	GER148, JULS43
*Scutellatia scutellarioides (*Kunth) R. Harley	Poleo de Gentil	Whole plant, fresh	Topical	1 bundle boiled for 5 minutes with 3l water. 1 bath per week.	Fright / Susto, Daño	ISA69
Stachys lanata Jacq.	Veronica (Macho)	Whola plant, dried	Oral	Boil 10g Veronica Macho with 1l water. Combine with Salvia, Matico, and Muyaca. Drink before or after meals. 3 cups per day for 15 days.	Bronchitis, Asthma	JULS13
Thymus vulgaris L.	Tomillo	Leaves, Stems and Flowers, fresh or dried	Oral	Boil 5g per 1l water. Drink 3 times per day.	Cough, Colic, Liver, Gases, Indigestion, Bladder	EHCHL169

Family/Genus/Species	Indigenous name	Plant part used	Admin.	Preparation	Use	Coll. #
LAURACEAE						
Aiouea dubia (H.B.K.) Mez.	Ishpingo	Seeds, fresh or dried	1., 3. Topical<br>2. Oral	1. Crush and boil 20 Seeds per 5l for 20-30 min. mix with Ishpingo, Achango, Pucho, Amala. Bath, every other day, 3 times a week. Do not leave bath outside, use every other day.<br>2. Crush Seeds, mix with Seeds from a specific seven other plants: Ashango, Pucho, Amala, Quina Quina, Mozcada, Cabalonga and boil in water. Drink 1 time per month<br>3. Boil 5 Seeds per 1/2l water or Caña Florida. Rub affected areas once a day.	1. Fright / Susto, Bad Air / Mal Aire<br>2. Bad Air / Mal Aire, Epilepsy<br>3. Rheumatism	EHCHL152
Cinnamonum verum J. Presl.	Canela	Bark, dried	1. Topical<br>2. Oral	1. 1l of water with 10g of canela and mix with petals of roses (red, white, yellow), Ramillete de Novia, Agua Florida, sugar, and 1 lime (its juice). Boil for 2 to 5 minutes. Take a bath in the solution 3 to 4 times a month. Alternatively grind and pulverize 100g. Rub powder throughout the body while praying and wishing for the person you are yearning for. 4 times a week or as needed.<br>2. 1l of water, 1 garlic clove, 10g of Matico, Veronica, Brochamelia, Vira Vira, 3g of Cinnamon. Boil for 3 to 4 minutes. Drink warm, 3 to 4 times a day as needed. After rituals drink cold a day after rituals occurance. Preferably in the morning during breakfast. As much as the patient feels is needed.	1. Good luck, Love, Enchantment<br>2. Bronchitis, Recovering after working all night on rituals	JULS122, GER101
Nectandra floribunda Nees	Ishpino, Flor de Ishpingo, Hierba de Ishpingo	1. Seeds and Bark, fresh or dried<br>2. Leaves and Flowers, fresh or dried	1. Oral<br>2. Topical	1. Mixed with Seeds from specific seven other plants: Ashango, Pucho, Amala, Quina Quina, Mozcada, Cabalonga. Boil 5 Seeds per 1/2l water, 20 min. Once a month for prevention. 1l per day, 7-15 days for illnes.<br>2. Limpia, combine with Timolina and Bully Vinegar, 3 times per week. Alternatively as bath, 20g per 3-5l water, boil for 20-30 minutes mix with Ajenco, Ruda, Romero, Albahaca, Ortiga, Añasquero, Hierba del Aire, Hierba del Susto, Romero, Hierba del Gallinazo. 3 times per week or once a month for prevention.	1. Bad Air / Mal Aire, Epilepsy<br>2. Fright / Susto, Bad Air / Mal Aire, Nerves, Epilepsy, Enchantment (prepare for)	EHCHL28, VFCHL47, RBU/PL302

Family/Genus/Species	Indigenous name	Plant part used	Admin.	Preparation	Use	Coll. #
Nectandra reticulata (R. & P.) Mez.	Ishpingo, Espingo-blanco, Espingo	1. Seeds, fresh or dried<br>2. Whole plant, fresh or dried	1. Oral<br>2. Topical	Boil with Hierba de Chocho, Cordon de Muerto, Claveles and Eucalyptus (10g total). Both bathe and rub the solution over body. 3 times only, any day. Alternatively grind 100g of Ashiango and 100g (combined) of Ishpingo, Cedron Seeds, Samala and Quina Quina. Blow ground powder on patients face. 2 times a week for 4 months.	1. Nervous system<br>2. Fright / Susto	RBU/PL379, JULS151, GER67
Persea americana Mill.	Palta	1. Leaves, fresh or dried<br>3. Flowers, fresh<br>2. Seeds, fresh	1., 2., 3. Oral	1. Boil 1/2l of water with 10 Palta Leaves for 3 minutes. Patient should drink hot solution. 1 cup 2-3 times a day for 1 - 2 months.<br>2.Grind 1 Palta Seeds (for several dosis). Boil 10g of Palta Seeds grind in 1/2 cup of water for 3 minutes. Add Linaza and sugar. Patient should drink lukewarm solution. 1 cup 4 times a day for a month. If used 3 times on a row the woman will become sterile.<br>3. Boil Flowers in water. Patient should drink lukewarm solution. 1/2 a cßup 3 times a day for a week.	1. Lose weight<br>2. Diarrhea, Kidneystones, Contraceptive, Sterilization for women only<br>3. Cough	JULS211, GER18
LECYTIDACEAE						
Gustavia augusta L.	Chope	Leaves, fresh	Topical	Heat 300g of Chope Leaves and 20g of archaeological chalk in a pan for a few minutes. Place warm on affected area and masage. Twice a day, especially when the hives are out or there is a break out.	Allergies, Rashes, Pimples, Hives	GER30
LEMNACEAE						
Lemna minuta H.B.K.	Flor de Agua	Whole plant, fresh	Topical	Mix 4 spoonfuls Flowers with 2 egg whites, put Flowers with egg whites over the stomach. Use 4 spoonfuls, 4 hours as poultice.	Ulcers, Inflammation of the stomach	ISA21
LILIACEAE						
Allium odorum L.	Cebolla China, Cebolla	Whole plant, fresh	1. Oral<br>2. Topical	1. Dice 15 onions in a bowl. Add a glass of water and 1/4kg of white sugar. Add a piece of ginger (can also add hen fat). Boil and stir until thick. Drink syrup at all temeratures, 1 spoonful every 6 hours for 1 week. Juice can also be drunk naturally.<br>2. Crush 1 1/2kg of Cebolla and strain in a piece of cloth to get all the extract. Discard the juice and use the rest. Place on top of the affected area and cover with a piece of cloth. Every other day in the AM for 3 days.	1. Bronchitis, Asthma<br>2. Bruises, Bad Air / Mal Aire, Blood clots	JULS129, GER36

Family/Genus/Species	Indigenous name	Plant part used	Admin.	Preparation	Use	Coll. #
Allium sativum L.	Ajo	1., 2. Clove, fresh<br>3. Peel, fresh	1. Oral<br>2. Topical<br>3. Incense	1. Add 3 garlic cloves, 1 Chinese onion, Matico, Corcionera, Eucalypto, Vira Vira, white sugar and 1/2l of water or cow milk into a pot and boil for 3 minutes. Drink warm, 2 tablespoons twice a day, for 1 week. Can also be eaten raw.<br>2. Crush 250g of garlic. Add it to 10g Eucalypto, 90g of alcohol, pacra, chuchuhasi and ginger. Let these ingredients soak in 1l alcohol for 1 week. Masage and rub the mixture on affected areas. Rub 1-2 times a day, as needed.<br>3. Burn 1kg of Peel in top of a charcoal burning jar. Smoke the house. Once a week for 4 weeks. Only on Tuesday's or Thursday's.	1. Cough, Bronchitis, Cold<br>2. Bruises, Arthritis, Rheumatism, Bad/ Air / Mal Aire<br>3. Mal de Aire, Removing bad spirits from the house	JULS92, GER37
Dracaena fragrans Ker Gawl.	Flor Dracena	Leaves and Stems, fresh or dried	Oral	10g per 1l water and boil. 3 cups per day, according to treatment.	Cough, Bronchitis, Asthma	RBU/PL334
Hesperoziphium niveum (Rav.) Rav.	Hierba de la Justicia, Piti Piti, Totorilla	Whole plant, fresh	1. Seguro<br>2. Oral<br>3. Topical	1. Put inside a bottle 10g of Hierba de la Justicia and add Hierba de la Plata, Dollar, Fortuna, Señorita, Valeriana Estrellada, perfume Tabu, Agua Florida, Lima juice, sugar, and Agua Bendita. Keep seguro in bedroom. As alternative put together in a cloth 10g of Hierba de la Justicia, 10g of Hierba del Dominio and Hierba del Olvido. Seal and pray. Patient must carry the bag and pray.<br>2. Have patient eat 3 plants. Oral fresh. Patient must be kept from the light, staying inside for 1 week. Will probably cause vomiting. After consumption patient has to follow a strict diet of no spices at all for 1 week. The person who eats this cannot see the light for 7 consecutive days.<br>3. Boil 1 Hierba de la Justicia herb with some water for 3-4 minutes. Bathe 3-4 times per month.	1. House protection, Health protection, Succeeding professionally at work, Judgement, Wounds (rebellious), Fragrance, Good Luck, Love, Dominating judgment (ritual), Dominating legal problems (ritual)<br>2. Mal daño through the mouth, Daño, Purgative, Sorcery<br>3. Spiritual Flowering	JULS269, TRUVan/Erica9, TRUBH27, RBU/PL325, JULS87, EHCHL79, GER93
LINACEAE						
Linum sativum L.	Linaza	Seeds, dried	Oral	1 Tbsp or 5g, 10g of Cola de Caballo, Chanca Piedra, Cana Cana, Boldo, and Overo. Boil the mixture for 5 minutes, then let mixture cool. Can mix with Cola de Caballo, Boldo. Take 1 cup, 3-4 times a day, for 2 weeks to 1 month. Drink lukewarm.	Inflammation of the kidneys, Liver, Inflammation, Inflammation of the prostate, Galbladder stones, Kidneystones	EHCHL159, JULS185, GER139
Linum usitatissimum L.	Linaza	Seeds, dried	Oral	1 Tbsp or 5g, 10g of Cola de Caballo, Chanca Piedra, Cana Cana, Boldo, and Overo. Boil the mixture for 5 minutes, then let mixture cool. Can mix with Cola de Caballo, Boldo. Take 1 cup, 3-4 times a day, for 2 weeks to 1 month. Drink lukewarm.	Inflammation of the kidneys, Liver, Inflammation, Inflammation of the prostate, Gall bladder stones, Kidneystones	EHCHL159, JULS185, GER139

Family/Genus/Species	Indigenous name	Plant part used	Admin.	Preparation	Use	Coll. #
LOGANIACEAE						
Buddleja utilis Kraenzl.	Flor Blanca	Flowers, fresh or dried	Oral	Boiled for 3-5 minutes. 1 Tbsp with 1l boiled water and mix with Grama Dulce, Hierba del Apostema. Drink 1l daily, 3-8 months.	Menstruation, Inflammation of the womb, Ovarian cysts, Inflammation of uterus, Inflammation	RBU/PL333, EHCHL38, ISA60, JULS155, GER136
LORANTHACEAE						
Psittacantus chanduyensis Eichler	Suelda con Suelda	Leaves and Stems, fresh	Oral, Topical	5g per 1l water. Tea: 1l per day, 1 month. Emplasto: 2 times per month.	Fractures, Twists, Bone rupture	RBU/PL269
Tristerix longibracteatus (Des.) Barlow & Wiens	Suelda con Suelda	Whole plant, dried	Oral	Add 10g of plant material with 5g of Uña de Gato and Diego Lope and 1l of water. Boil the mixture for 4 minutes. Drink the mixture lukewarm. Take 1 cup, 3 times a day, for 1 month.	Bones (lacking Calcium), Vaginal discharge (white or yellow), Bones (fractured)	JULS296, GER74
LYCOPODIACEAE						
Huperzia crassa (H. & B. ex Willd.) Rothm.	Condor, Condor Amarillo, Condorcillo, Condorcilla, Condor Rojo, Condor Verde	Leaves and Stems, fresh	1. Seguro<br>2. Topical	1. 3 small Branches / Leaves<br>2. 20g per 5l, boil 20 min, combined with herbs of strength and of luck, bathe 2-3 times per week, during the evening.	1., 2. Good luck and succes in travels, Fragrance, Bad Air / Mal Aire	ISA140, RBU/PL352, RBU/PL356, TRUBH23, RBU/PL351, EHCHL144, RBU/PL353
Huperzia cf. *columnaris* B. Oellg.	Hornamo Condor Purga	Leaves and Stems, fresh	Oral	For 20 patients boil 2 San Pedro's (1 of 7 lines and 1 of 8 lines) and 100g of Condor Purga in 4l of water for 3 hours. Drink cold, 1/2 cup one time only.	Laxative	GER106
Huperzia hohenackeri (Herter) Holub	Guaminga	Whole plant, fresh or dried	1. Oral<br>2. Seguro	1. 5g per 1l boiling water, 1 time per day.<br>2. Seguro, use 7 small plants per Seguro.	1., 2. Fright / Susto, Purgative, Bad Air / Mal Aire, Work, Love	TRUVan/Erica4
Huperzia kuestneri (Nessel) B. Ollg.	Condor Lasio, Trensa Hermosa, Condor Crespo, Condor Simuro, Condor Mise	Whole plant, fresh or dried	1. Topical<br>2. Seguro	1. Boil 3 leaves of the following plants: Condor Simuro, Mishia Galga, Semora Curandera, and mix with the following 6oz perfumes: Jardin España, Tabu. Add 1 12oz bottle of Agua Florida and 1 12oz bottle of Cananga, and boil in 1/2 cup of water for 10 minutes. Drink cold. Patient must stay in a dark room, isolated and on a diet without spices for 3 days. Afterwards, patient may come out of the dark room, but must rest inside the house for another 3 days.<br>2. 7 small branches per Seguro.	1., 2. Luck, Fragrance, Break Sorcery, Work, Love	RBU/PL357, TRUVan/Erica3, TRUVan/Erica1, GER59, TRUVan/Erica15
Huperzia reflexa (Lam.) Trevis.	Condor Mulato, Enrededera	Leaves and roots, fresh	Topical	20g with 1l water and boil 30 min, with other herbs for strength. Bathe 3 times a week.	Fragrance, Good Luck, To prevent someone from moving, To cause someone to return	RBU/PL359, EHCHL113
Huperzia sellifolia B. Ollg.	Condor Crespo	Whole plant, fresh or dried	Topical	10g per 1l boiling water, 2 baths per month, during the evening.	Luck, Fragrance	RBU/PL356(a)

Family/Genus/Species	Indigenous name	Plant part used	Admin.	Preparation	Use	Coll. #
Huperzia tetragona (Hook. & Grev.) Trevis.	Trencilla Roja	Stems, dried	Topical	20g per 5l water and boil 20 min mix with herbs of strength like Hornamos and Maiques. Bathe 3 times per week.	Fractures, Good Luck	RBU/PL354
Lycopodium clavatum L.	Trencilla Verde, Destrencilla	Whole plant, fresh or dried	1. Oral<br>2. Seguro	1. 5g per 1l boiling water, 1 time per day.<br>2. Seguro, use 7 small plants per Seguro.	1., 2. Work	RBU/PL348, TRUBH 4, GER154
Lycopodium jussiaei Desv. ex Poir	Hierba del Hombre, Rastrera	Whole plant, fresh or dried	1. Oral<br>2. Seguro	1. 5g per 1l boiling water, 1 time per day.<br>2. Seguro, use 7 small plants per Seguro.	1., 2. Work	TRUBH3
Lycopodium thyoides H. & B. ex Willd.	Trencilla Roja	Stems, dried	Topical	20g per 5l water, boil 20 min mix with herbs of strength like Hornamos and Maiques. Bathe 3 times per week.	Luck, Bad Air / Mal Aire, Love, Succes, Business	EHCHL124
LYTHRACEAE						
Cuphea strigulosa H.B.K.	Lancetilla, Gacetilla, Sanguinaria, Gansetilla, Hierba del Toro	Leaves and Stems, fresh	1. Topical<br>2. Seguro<br>3. Oral	1. Alternative mixture for Spiritual Flowering, see below. Bathe once.<br>2. Standard Seguro mixture, see below.<br>3. 5-20g per 1l for 3 min, combine with Congona, Claveles, and Madre Selva, Ortiga, Moradilla, Contrahierba, Colores, Agujilla, Colcacur, Pie de Perro, Cola de Caballo, Verbena, Pimpinela, Flor Blanca, Grama Dulce, Esencia de Rosa and Cadillo. Drink 3-4 times per day, 1l daily, take 1 week - 3 months. Patient should drink solution before eating.	1. Spiritual Flowering<br>2. Good Luck<br>3. Blood circulation, Fever, Blood purification, Intestinal infections, Heart, Nervous system, Blood, Liver, Discharges, Colic, Gases, Diarrhea, Inflammation of the stomach, Kidneys, Internal Inflammation, Strengthen the body, Anemia, Bad Air / Mal Aire	GER104, EHCHL35, VFCHL34, JULS33, ISA51, RBU/PL259, EHCHL43, JULS59, ISA53, GER147
MALESHERBIACEAE						
Malesherbia ardens Macbr.	Veronica	Whole plant, fresh or dried	Oral	Boil 5g per 1l, combine with Contilo, Arabisca, and Huamanripa. Drink Three times per day to total 1l daily.	Cold, Cough, Bronchitis, Asthma	EHCHL139
MALPIGHIACEAE						
Banisteriopsis caapi (Spruce ex Grieseb.) Morton	Ayahuasca, Ayauasca, Ayahuasca Verde, Ayahuasca Amarilla	Bark, fresh or dried	Oral	1l of water with 20g of the Bark. Boil from 12 noon until 4pm on a low fire, then increase temperature toward the end. Drink cool, One small cup during ceremony. One needs to fast for 24 hours before taking the drink prepared. Patient cannot be on menstrual period.	Enhancing vision during rituals	JULS109, GER65, GER239
MALVACEAE						
Alcea rosea (L.) Cavanilles	Malva Blanca, Malva Morada	Whole plant except Stems, fresh	Oral	10g per 1l water. Use Flowers for cough and hemorrhages. Drink 3 times per day, as needed.	Inflammation, Cough, Hemorrhages	JULS78, JULS79

Family/Genus/Species	Indigenous name	Plant part used	Admin.	Preparation	Use	Coll. #
Gossypium barbadense L.	Algodon Pardo, Algodon	1. Seed hairs, dried<br>2. Seeds, fresh	Topical	1. Remove the Seeds from the cotton and combine it with a mix of the heart of 2 shredded totoras, 2 aji peppers, and one unbroken egg. Rub the patient with the cotton and mix all over the body. Use the Seedsles cotton to make a sign of the cros on the patient. Then burn the cotton in a faraway place. If the cotton disolves while rubbing, the patient is very sick. Crack the egg in a glass of water and look for signs of illnes in it.<br>2. Grind 200g of Seeds and extract oil. Place on top of affected area once a day until healed.	1. Evil Eye (children)/ Mal Ojo (niños)<br>2. Wounds (external)	JULS98, GER 262
Malva parviflora L.	Malva Rosa, Malva Real	Leaves, fresh	1. Oral<br>2. Topical	1. Combine 1l of water with 10g of Pie de Perro, Chacuro, Verbena, Cola de Caballo, Amor Seco, and Unaza. Also add 3-4 Leaves of Malva. Boil the mixture for 3 minutes. Patient should drink lukewarm solution. Take 1 cup, 3-4 times a day, for 1 month.<br>2. Can also be applied as poultice.	1., 2. Liver, Inflammation (general), Cough, Bronchitis, Coughing with blood	JULS189
Malva sylvestris L.	Malva (Chica), Malva Blanca	Leaves and Stems, fresh or dried	1. Oral<br>2. Topical<br>3. Topical	1. 20g per 1l water for 3 min. Mix with Toronjil, Pimpinela, Mejorana, Pensamiento, and Cedron. Drink 1l per day, 15 days.<br>2. Bath, Boil 20g per 2l water for 20 minutes, wash 3 times per week.<br>3. Boil 10-15g per 1l for 10 minutes combined with Conchalagua, Amaro, Chicoria. Enema 1 time per month.	1. Fright / Susto, Bad Air / Mal Aire, Heart, Nerves, Tachycardia, Epilepsy (initial stages)<br>2. Wounds, Vaginal cleansing<br>3. Intestinal cleansing	VFCHL49, EHCHL29
Urena lobata L.	Buenas Horas	Whole plant, fresh	Oral	The plant should only be gathered in the afternoon. Boil 100g of plant with 1 cup of water for 5 minutes. Drink cold, 1/2 cup before bed 1 time a day for 15 days or as needed.	Mental illness, Memory loss, Confusion	Ger212
MELASTOMATACEAE						
Brachyotum tyrianthium Macbride	Sarcilleja	Stems, fresh	Oral	5g per 1l water and boil 3-5 minutes. Drink, three times per day for three days.	Blood circulation	EHCHL55
Miconia salicifolia (Bonpl. Ex Naud.) Naud.	Llatama roja	Leaves and Stems, fresh or dried	Oral	Boil 100g in 1 cup of water for 5 minutes. Drink cold, 1/4 cup only once.	Bad Air / Mal Aire, Burns	GER83
Tibouchina laxa (Des.) Cog.	Barbon	Flowers, fresh	Topical	Crus and extract juice, put in your eye as if it were eyedrops, 2 drops per eye, 2 times per day.	Cataracts	ISA22
MENISPERMACEAE						
Abuta grandiflora (Mart.) Sand.	Abuta (male and female)	Root and Stems, fresh or dried	Oral	Boil 20-100g with 1l water for 4-5 minutes. Drink warm. Take 1 cup, three times a day. Take 3 days before and 3 days after menstrual period.	Contraceptive, Diabetes, Cholesterol	JULS88, RBU/PL312

Family/Genus/Species	Indigenous name	Plant part used	Admin.	Preparation	Use	Coll. #
MONIMIACEAE						
Peumus boldus Molina	Boldo	Leaves, dried	Oral	1l of water and 10g of Boldo, Pie de Perro, Linaza, Berros, Pata de Perro, Papa Madre, Espiga de Maíz and Flor de Overo. Boil for 2 to 3 minutes. Drink warm, 1 cup 3 to 4 times a day for 1 month.	Inflammation of the liver, Kidney Inflammation	JULS114, GER157
Spiaruna aspera (R. & P.) A.DC.	Rinchinchin, Chinchin	Leaves and Stems, dried	Topical	Ground 100g of the plant material until it is completely pulverized. Blow the powder into the face of the person to whom you want to cause trouble and mention his/her full name. 1 time per ritual. 3 rituals.	Causing trouble for someone, Causing break-ups in other couples or families	GER96
Siparuna muricata (R. & P.) A. DC.	Añasquero, Hojas de Añascero, Añasquero (Grande)	Leaves and Stems, dried	Topical	Boil 5l of water with 100g of: Anasquero, Hierba del Susto, Ishpingo, Romero, Ruda Hembra, Ishpinguillo, Chuque, Palo Santo and 7 Espiritus for 10 min. First: Rub body with herbs Second: Rinse with the water. Third: Do not dry with towel. Also to be used as poultice, 3 times a week to 3 times per month.	Fright / Susto, Arthritis, Rheumatism, Bone pain, Muscle pain, Stomach pain, Daño, Sorcery, Gases, Colic	GER88, EHCHL129, ISA113, ISA64
MORACEAE						
Brosmium rubescens Taubert	Palo Sangre, Palo de la Sangre, Ablita	Wood and Bark, fresh or dried	Oral	1. In wine (Abuelo) add Palo de Sangre (5g), Palo Huaco, Cascarilla, Chuchuhuasi, Pacra, honey, pollen, Huevo de Angelote. Add to the wine Huanarpo Macho if its for a men and Huanapo Hembra if its for a woman. Drink 1 cup 3 times a day until bottle its finished.<br>2. Chop the small sticks and boil 1 Tbsp Ambarina with 1l water with 50g of Palo Sangre and 50g of Palo Huaco for 10 minutes. Drink 1l daily, 3 months or more.<br>3. 7 roots or 50g per 1 bottle of Whiskey or Tequila mixed with Chuchuwasi, Cascarilla. Drink during meals, two times per day for 8-10 days.	1. Fertility, Sexual potency<br>2. Blood irrigation, Blood coagulation, Haemorrhages (prevention and healing), Diabates<br>3. Arthritis, Bronchitis, Muscle pain	JULS209, ISA49, EHCHL64, RBU/PL311, GER86, EHCHL62
Ficus carica L.	Higo	Leaves and Stems, fresh or dried	Oral	Boil in 1l of water 4 leaf for 3 min. Drink lukewarm, One cup 3-4 times a day as needed.	Diabetes	JULS165
Ficus spp.	Higueron	Bark, fresh	Oral	Macerate in any kind of alcohol. Drink 2 glasses per day.	Bones (fractured)	RBU/PL310
Morus alba L.	Morera	Leaves and Stems, fresh or dried	Oral	Boil in 1l of water 4 leaf for 3 min. Drink lukewarm, 1 cup 3-4x per day or as needed, for life.	Diabetes	JULS197

Family/Genus/Species	Indigenous name	Plant part used	Admin.	Preparation	Use	Coll. #
MUSACEAE						
Musa x paradisiaca L.	Platano	1. Flowers, fresh<br>2., 3. Latex of the Stems, fresh	1., 2.Oral<br>3. Topical	1. Boil 1l water, then add 10g Platano. Cover and boil briefly. Remove, and let mixture sit for 3 minutes. Take 1 cup, 3 times a day, as needed.<br>2. Add 5oz of plant material, 5oz of port wine, 2oz of Polen and 2 tablespoonfulls of honey. Drink the syrup. Take 1 tablespoon every 6 hours for 1 month.<br>3. Cut the Platano trunk with a machete. Juice ("blood") will come out and is collected in a container. Place extract on top of the affected area (cover completely). 1 per day until the wound is healed.	1. Diabetes<br>2. Asthma, Pulmonary Disease, Malaria, Dengue<br>3. Wounds, Stops bleeding	JULS228, GER16
MYRICACEAE						
Myrica pubescens H. & B. ex Wild.	Laurel	Leaves and Stems, dried	Topical	Bath, 5g boiled with 3l water, mixed with Sauco, Nogal, Hierba del Susto. 2-4 times per month. for wounds wash morning and afternoon.	Fright / Susto, Sorcery	ISA84, ISA128
MYRISTICACEAE						
Myristica fragrans L.	Nuez Moscada, Ajonjoli	Seeds, dried	1., 2. Oral	1. Grind Seeds and boil in 1l water 1 Seeds to make 4 glasses. Drink 4 cups per day, 7-15 days. Alternatively macerate Nuez Moscada with 10g of Ajonjoli with 1 bottle of Abuelo wine, 10g each of Palo Sangre, Palo Huaco, bee honey, Pacra, Huanarpo Macho, bee pollen, Huevo de Angelote and Para Para. Take 1 cup in the mornings, middays and evenings until bottle is finished.<br>2.Grind Seeds and boil in 1l water with with Seeds from a specific seven other plants: Ashango, Pucho, Amala, Quina Quina, Ishpingo, Cabalonga. Once a month.	1. Nervous system, Cough, Colic, Bad Air / Mal Aire, Asthma, Gases, Vitamins, Bronchitis, Fertility, Sexual potency, Bone pain<br>2. Bad Air / Mal Aire, Epilepsy	RBU/PL385, EHCHL155, JULS292, GER197
MYRTACEAE						
Eugenia obtusifolia Cambes.	Unquia Real, Rumilanchi	Leaves and Stems, fresh or dried	Oral	Chop or break the plant and put in boiling water for 2 to 3 minutes. Drink 3 times a day for up to 1 month.	Inflammation (general)	JULS32
Eucalyptus citriodora Hooker	Citrodora	Whole plant, fresh or dried	Oral	10g per 1l, boil 3 min Drink beverage prepared. 3 times per day, for life.	Diabetes	JULS60

Family/Genus/Species	Indigenous name	Plant part used	Admin.	Preparation	Use	Coll. #
Eucalyptus globulus Labill.	Alcanfor, Eucalipto Serrano, Eucalipto	1. Leaves, dried<br>2., 3. Leaves, fresh or dried	1., 2., 4. Oral<br>3. Topical	1. Boiled, cover the head with steam for 15 minutes. Boil 10gr in 1l water, combined with Manzanilla, Matico, Nogal, Ajos Giro and Chilca. Inhale 1 time per week, 3-4 times a month.<br>2. Boil 20g of Eucalyptus and 10l of water with Cerraja, Borraja, Vira Vira, Manzanillon, Romero, Lavanda and Hortiga can be added as well, sit in the steam once a week for mild condition, twice a week for severe condition.<br>3. Bath, 500g Eucalipto boiled with with Chilca, Palo Santo, Romero, Ajos Giro. 2 times a month, do not use too much because plant is very hot, patient must be naked and covered with a sheet over his head, then sitting to absorb the vapor for 20 minutes. Stay inside home for 24 hours after the bath. 1 every 30 days. 2 times only.<br>4. 10g per 1l, boil 2 min, combined with Muniaca, Escorcionera, Veronica, Humanripa, Zarzamora, Matico. 2-3 cups a day, 3-4 times a day for approximately 2 weeks, patient should drink hot.	1., 2., 4. Bronchitis, Respiration, Cold, Cough, Sinusitis, Asthma<br>3. Cold, Rheumatism, Bone Pain, Congestion, Burn fat	ISA130, JULS61, VFCHL35, JULS153, GER14, EHCHL12
Myrcianthes discolor (H.B.K.) Vaughn	Lanche, Mirto	Whole plant, fresh	Oral	Boil 5g per 1l of water to create jelly or tea, drink breakfast, lunch, and dinner, 3 cups per day, for 1 month.	Food, Memory, Cerebral, Vitamins for the brain and for colds, Inflammation, Rheumatic pain, Stomach, Menstrual regulation	ISA34, EHCHL17, RBU/PL271
Myrcianthes fragrans (Sw) McVaugh	Lanche, Mirto	Whole plant, fresh	Oral	Boil 5g per 1l of water to create jelly or tea, drink breakfast, lunch, and dinner, 3 cups per day, for 1 month.	Food, Memory, Cerebral, Vitamins for the brain and for colds, Inflammation, Rheumatic pain, Stomach, Menstrual regulation	ISA34, EHCHL17, RBU/PL271
Psidium guajava L.	Hoja de Guanábana, Graviola	Leaves and Stems, fresh or dried	Oral	5 Leaves 1l water, boil 3 min. Used alone, no other plants. Take before and after food 1l per day or 3-4 cups for one month.	Cancer, Liver sicknes	VFCHL24
Scutia spicata (H. & B. ex Schultes) Weberb. var. *spicata*	Pial, Pus	Stems, fresh or dried	Charm	Put the Stems in a cross formation and tie with a red ribbon. Place cross behind the house.	Keeping evil spirits away from the house	JULS226
Syzygium aromaticum (L.) Merr. & Perry	Clavo de Olor	Flowers bud (clove), dried	1. Topical<br>2. Oral	1. The patient chews the clove with the aching tooth. Take 1-2 cloves a day, as needed.<br>2. Boil 1/2l of water, then add 10 cloves. Cover the mix and let it sit for 2-3 minutes. Drink the infusion. Exceeding doseage can lead to kidney damage. Take mixture 2 times a day, for 2-3 days.	1. Toothache<br>2. Stomachache	JULS143, GER155

Family/Genus/Species	Indigenous name	Plant part used	Admin.	Preparation	Use	Coll. #
Syzygium jambos (L.) Alston	Poma Rosa	Fruits and Leaves, fresh	Oral	1 cup of water and 20g of the leaf and Fruit and boil for 5 minutes. Drink ciold, 1/4 cup 1 time a day for 8 days.	Diarrhea	GER173
NYCTAGINACEAE						
Boerhavia coccinea Mill.	Pega Pega	Whole plant, fresh	1. Topical<br>2. Seguro	1. Alternative mixture for Spiritual Flowering, see below. 3 baths per month, during the evening.<br>2. Standard Seguro mixture, see below.	1. Spiritual Flowering<br>2. Good business, Protection, Good fortune, Good health	GER122, RBU/PL347
Mirabilis jalapa L.	Buenas Tardes	1. Flowers and Leaves, fresh<br>2. Root, fresh	1. Topical<br>2. Oral	1. Place ground leaf and Flowers on affected area and put a piece of cloth over it. 2 times a day as needed.<br>2. 50g of each of the following: Buenas Tardes, Paja de Lagartija (Flor de Arena), and 1 cup of water. Boil for 5 minutes. Add honey. Drink lukewarm, 1 cup 3 to 4 times a day for 1 month.	1. Bruises, Varicose veins<br>2. Renal disease, Inflammation, Prostate, Kidneystones, Pre-prostate cancer	JULS116, GER185
OLACACEAE						
Heisteria acuminata (H. & B.) Engler	Chuchuasi, Chuchuhuasi	Bark, fresh or dried	1., 2. Oral<br>3. Topical	1. Crush Bark and put in 1 bottle of wine to macerate. Drink 1 cup 3 times a day for 15 days, stop for 15 days, then start treatment again for 15 more days.<br>2. Mix 10g of Bark with 1 bottle of wine (abuelo) add honey, Pacra, Huevo de Angelote, Cholitos, Huanarpo (Macho and Hembra). Drink 1 small cup 3 times a day as needed.<br>3. Boil 5l of water plus 100g of Chuchuhuasi, 100g of Eucalypto, Moy, and Bichayo for 30 minutes. Patient must be in an enclosed room without clothes and with a towel over the head. Patient should inhale the steam coming out of the tizana and afterwards rub body with the herbs. 1 time only or as needed or every 3 months.	1. Cold, Cough, Bones, Arthritis<br>2. Fertility, Sexual potency<br>3. Arthritis, Muscle pain, Bone pains, Sprains, Colds, Burning fat, Burning cholesterol	RBU/PL287, JULS138, GER164
Ximenia americana L.	Limoncillo	Whole plant, fresh or dried	Oral	Boil 1l of water, then add 10g total of Limoncillo, Panisara, Inojo, Ajenjo, Toronjil, and Pimpinela. Let mixture sit for 3 minutes. Patient may drink at any temperature. Take 1 cup, 3-4 times a day, for 1 month.	Nerves, Stomach, Menstrual regulation	JULS184
OLEACEAE						
Olea europaea L.	Hojas de Olivo, Olivo	1. Leaves, fresh<br>2. Leaves, dried	1. Oral<br>2. Incense	1. Tea, 3g per 1l of water mixed with Muña,Corpus Way, 3 times per day for 8 days<br>2. Place a handful of Olivo, Mirra, Palo Santo, incense, and Romero on top of burning charcoal. Smoke for rituals. Pas the smoke throughout the patient's body and around the house, repeat 2-3 times a week, or as needed.	1. Diabetes, Colic<br>2. Dispelling negative energy from the house	EHCHL86, JULS204

Family/Genus/Species	Indigenous name	Plant part used	Admin.	Preparation	Use	Coll. #
ONAGRACEAE						
Epilobium sp.	Hierba Rabia	Whole plant, fresh	Oral	1 Tbsp with 1l water. Can combine with Pimpinela, Cadillo, Colores, Lancetilla. Drink 1l daily for 3 months.	Moodines, Grumpiness, Intoxication of the blood, Anger, Rashes from intoxication, Ingestion of toxic medicine	ISA46
Fuchsia ayavacensis H.B.K.	Conchalalay, Conchalalay Colorado	Leaves and Stems, fresh or dried	1., 2. Topical	1. 5g mixed with Sauco, Nogal, Salvia, Añasquero Grande and 7 Espiritus with 3l boiled water. Boil for 1 hour, then let cool down to tepid temperature (lukewarm). 2 Baños per week in agreement with what La Mesa indicates or twice a month.<br>2. Use fresh Leaves. Combined with Conchalay Blanco and Guaminga, 7 Espiritus, Timolina, Bully Vinegar. Use as poultice 2 per week in agreement with what the mesa indicates.	Cold, Daño, Fright / Susto, Swelling, Arthritis (beginning)	ISA82, ISA1
Oenothera rosea Aiton	Hierba del Dominio	Whole plant, fresh or dried	Oral	5g per 1l water. 4 cups per week, 2 weeks.	Decreasing bad character	RBU/PL366
ORCHIDACEAE						
Aa paleacea (H.B.K.) Rchb. f.	Hierba de la Soledad, Hierba Sola	Leaves, fresh	1., 3. Oral<br>2. Seguro	1. Boil 1 leaf per cup water, drink once a year.<br>2. One leaf per seguro.<br>3. 3-5g per 1l water. Mix with Tapa Tapa, Sicana. Drink 1l daily, 1 week each month.	1., 2. Depression, Loneliness<br>3. Contraceptive, Sterilization of women	ISA141, EHCHL75
Epidendrum calanthum Rchb. f.	Sémora Negra, Sémora Curandera	Leaves and Stems, dried	Oral	Boil 1 cup of water with 50g of plant material. Drink cold once a day.	Bad Air / Mal Aire, Mal de susto	GER79
Lycaste gigantea Lindl.	Caña Caña	Stems, fresh	Oral	Add 10g of the plant material, 10g of Linaza, Berro, Pata de Perro, Papa Madre, Espiga de Maiz, and 1/2l of water. Boil the mixture for 5 minutes. Drink cold, 1/2 cup twice a day, for 8 days.	Kidney inflammation	GER156
Pachyphyllum pastii Krenzl. ex Weberb.	Guaimi Guaimi, Huami Huaimi, Huaime Huaime	1. Leaves, fresh<br>2. Stems, fresh	1. Topical<br>2. Seguro	1. Boiled, 10g per 5l water with other strong herbs. Use 3 times a week.<br>2. Add a small Stems tio the seguro, together with herbs of luck.	1., 2. Self defense, to arm against everything	EHCHL97
Stelis eublepharis Rchb. f.	Hierba del Oro, Boton de Oro	Whole plant, fresh	1. Seguro<br>2. Topical	1. One Stem / 3g, combined with Hierba de la Plata, Hierba de la Justicia, Hierba del Dominio, Encanto, Sigueme Sigueme and plants of strength and luck.<br>2. Bath, boiled 20 minutes, 200g per 8L water, 3 times per week for 1-6 months.	1., 2. Fragrance, Good Luck, Nerves, Luck in love, Luck in business, Good luck for work, Good travels	RBU/PL342, EHCHL9, VFCHL40

Family/Genus/Species	Indigenous name	Plant part used	Admin.	Preparation	Use	Coll. #
Stelis sp.	Huaime-Huaime, Cucharilla	1. Whole plant, fresh<br>2. Root, dried<br>3., 4. Leaves and Stems, fresh	1., 2., 3. Topical<br>4. Seguro	1. Crush the plant and heat up with Agua Florida. Use 10g of the crushed plant and 1 oz of Agua Florida. Place emplasto on the opposite sides of the affected area and cover with a piece of cloth. 2 times a day for 2 days.<br>2. Bath, 5g per 1l boiling water, 3 baths per month.<br>3. Florecimiento. Alternative mixture for Spiritual Flowering, see below.<br>4. Standard Seguro mixture, see below.	1. Bad Air / Mal Aire, Facial paralysis caused by Mal Aire, e.g. when a patient Leaves the house and is hit by mal Aire.<br>2. Inflammation of ovaries, Inflammation of uterus<br>3., 4. Good business, Protection, Good fortune, Good health	JULS169, RBU/PL296, EHCHL45, GER129
OXALIDACEAE						
Oxalis bulbigera Knuth.	Trebol	Leaves and Stems, fresh	Oral	Boil water, then add 10g of Toronjil, Poleo, Manzanilla, Hinojo, Romero, and other herbs. Let mixture sit for 2-3 minutes. Patient should drink lukewarm solution. Drink a glass 3 to 4 times a day for a month.	Heart, Nerves, Insomnia	JULS261
Oxalis tuberosa Molina	Oca Rosada	Tuber, fresh	Oral	Boil 7 to 8 tubers for 2 minutes. Oral 2 to 3 times a day for 2 weeks to a month.	Food, Sexual potency	JULS203
PAPAVERACEAE						
Argemone mexicana L.	Cardo Santo	Flowers, Leaves and Stems, fresh	1. Oral<br>2. Charm	1. 1l water and add 10g of Cardo Santo, mix with Cola de Caballo, Malva, Llanten, Pie de Perro. Drink 1 cup 2 to 4 times a day for 1 month.<br>2. Plant Cardo Santo near the house/chacra and recite a spiritual prayer to ensure the plant guards your property. Keep the plant forever and pray once.	1. Stomach ache, Inflammation (general)<br>2. Seguro de casa, Seguro de Chacra, Protecting the house	JULS126, GER176
PASSIFLORACEAE						
Passiflora caerulea L.	Pasionara	Flowers, Leaves and Stems, fresh	Oral	Boil 1l of water, then add 10g of Pasionaria, Toronjil, Pimpinella, Chancas de Comida, Romero, and Mebrillo Peels. Let it sit for 2-3 minutes. Patient should drink lukewarm solution. 1 cup 3 times a day for a month.	Nerves, Insomnia, Anxiety	JULS217
Passiflora edulis Sims.	Maracuya	Flowers and Fruit pulp, fresh	Oral	Add water to the pulp. Drink or consume at all temperatures. Take 1 Fruit a day, as needed.	High blood pressure	JULS193
Passiflora ligularis Jus.	Hoja de Granadilla, Granadilla	1. Leaves and new shoots, fresh<br>2. Peel of the Fruit, fresh	Oral	1. Combine 1l water with 10g of Granadilla. Add Boldo, Cola de Caballo, Chacur and Amor Seco. Boil the mixture for 3-5 minutes. Take 1 cup, 3-5 times a day for 1 month. Do not use if pregnant!<br>2. Boil 1l water, then add 3/4 of the Fruit Peel. Add Culen, Hinojo and Chancas de Comida. Drink hot. Take 1 cup, 3-4 times a day for 3 days, or as needed.	1. Liver, Blood circulation, Inflammation, Inflammation of the kidneys, Inflammation of the liver<br>2. Diarrhea	EHCHL47, JULS163
Passiflora quardrangularis L.	Hojas de Tumbo	Leaves, fresh	Oral	3 Leaves per 1l water, drink 3 times a day.	Liver, Menstrual pain, Stomachache	EHCHL135

Family/Genus/Species	Indigenous name	Plant part used	Admin.	Preparation	Use	Coll. #
Passiflora punctata L.	Tumbillo	Fruit, fresh	Oral	Oral 50g 3 times per day for 5 days.	Digestion	GER261
Passiflora sp.	Chulgan	Leaves and Stems, dried	Oral	Add 10g of plant material with 1 cup of water. Boil the mixture for 3 minutes. Drink hot. Take 1 cup only once.	Promoting vaginal dilation during childbirth.	JULS279
PHYTOLACCACEAE						
Gallesia integrifolia (Spreng.) Harms.	Palo de Ajo	Stems, dried	Oral	Boil 20g of Palo de Ajo with 1/2 cup of water for 2 minutes. Drink cold, 1/8 cup a day for 8 days.	Bronchitis, Asthma	GER116
Petiveria alliacea L.	Mocura, Mucura	Whole plant, fresh	1. Topical<br>2. Seguro	1. 5l of water, 1 bundle of Mocura (10g), add yellow, red, and white rose petals. Boil for to 2-3 minutes. Filter roses and let sit. Add 1 Tsp sugar, Agua Florida, and lime juice. Bathe when lukewarm. Pray while making the sign of the cros on the body and wash with the plants. Can only bathe during positive energies of the moon, no waxing or waning. Quantity is only for 1 person. Do not bathe until the following day. Bathe Tuesdays Fridays and Tuesdays.<br>2. 1 Stems in flask with typical seguro plants and herbs (Hierba de la Plata, Hierba de la Fortuna, Hierba del Lago, etc.). Take the flask with you if it is small, or keep it in the house if it is big. Only the person it was intended for can touch it.	1. Spiritual Flowering<br>2. Protection	JULS286
Phytolacca bogotensis H.B.K.	Laylambo, Ilambo	Flowers, Leaves and Stems, fresh	1., 2. Topical	1. Fresh Leaves as poultice. Use very rarely because it is too cold.<br>2. Fresh Leaves, 1 bundle with 3l water, mix with Añasquero, Ajenco, Sauco, Tres Hojas and Agua de Susto. Limpia or bath. Bathe 1-2 times per week with the warm mixture making sure to rub the patient with the Leaves. Advise the patient not to rinse after the bath.	1., 2. Daño, Fright / Susto, Sorcery, Malaria, Dengue, Yellow fever	ISA81, ISA111, JULS218
PINACEAE						
Pinus patula Schldl. & Cham.	Pino	Leaves and Stems, fresh	Oral	Boil for 5 minutes 1 cup of water with 100g of the plant material. Drink lukewarm, 1/4 cup once a day for 15 days.	Arthritis, Rheumatism, Bone pain	GER215
Pinus radiata D. Don.	Pino	Leaves and Stems, fresh	Oral	Boil for 5 minutes 1 cup of water with 100g of the plant material. Drink lukewarm, 1/4 cup once a day for 15 days.	Arthritis, Rheumatism, Bone pain	GER215
PIPERACEAE						
Peperomia fraseri C. DC.	Hierba de la Plata, Dollar	Flowers and Leaves, fresh	1. Seguro<br>2. Topical<br>3. Oral	1. 2 small Stems per Seguro<br>2. Boil 20 minutes, 10-50g per 8L for 30 minutes, combined with Condores, Hornamos, Trenzas, Hierba del Oro, Hierba del Cariño, Hierba de la Estrella. Bathe 3 times a week for six months.<br>3. 10g per 1l for three minutes, combined with Siempre Viva, Toronjil, Pimpinela, Romero, Mejorana, Pensamiento. Drink 1l per day for 1-30 days.	1., 2. Fragrance, Good luck, Love, Aphrodisiac, Business, Good travels, Heart, Nerves, Anxiety<br>3. Heart, Nerves, Anxiety	EHCHL7, RBU/PL341, VFCHL32, TRUVan/Erica16

Family/Genus/Species	Indigenous name	Plant part used	Admin.	Preparation	Use	Coll. #
Peperomia galioides H.B.K.	Congonilla	Leaves and Stems, fresh	Oral	5g per 1l, 1-2 min, combined with Malva Olorosa, Siempre Viva, Contrahierba, Toronjil. Put on the Mesa and later given to the patient. 1 Tsp, 3 times during the night fro prevention, or 2 cups daily for 3-4 days for treatment.	Nerves, Bind the boyfriend or husband, Depression, Bad Air / Mal Aire, Heart, Nervousness, Nostalgic anxiety, Emotional trauma	EHCHL95, VFCHL38, RBU/PL298
Peperomia hartwegiana Miq.	Hierba de la Plata, Hierba del Tesoro	Flowers, Stems and Leaves, fresh	1. Seguro<br>2. Topical	1. Standard Seguro mixture, see below.<br>2. Standard mixture for Spiritual Flowering, see below	1. Good business, Protection, Good fortune, Good health<br>2. Spiritual Flowering	ISA134, ISA92, GER127
Peperomia inaequalifolia R. & P.	Congona, Kongona	Whole plant, fresh	1., 3. Oral<br>2. Topical	1. Boil 5g per 1l. stronger with Congonilla, Torongil, Pimpinela, Meforana, and Pensamiento.The Stems and Leaves are used predominantly. Drink 3 to 4 times per day for 1 to 2 months.<br>2. Burn Leaves and inhale smoke.<br>3. Put it in someone else's food so that they forget a loved one.	1., 2., 3. Heart, Emotional pain, Epilepsy, Forgetting love or trauma, Forget problems, Forget pain of love, Forget bad relationships, Anxiety, Heart palpitation	TRUBH12, JULS30, VFCHL39, EHCHL8, RBU/PL297, GER80
Peperomia quadrifolia Trel.	Piri Piri, Piri-Piri (Macho y Hembra)	Whole plant, fresh	1., 2. Topical<br>3. Seguro	1. Boiled for 20 minutes with 20-50g per 3-5l of water, Stems and leaf of Piri-Piri (Macho y Hembra) plus a bit of the following: Hierba del Buen Querer, Palmerilla, Destrencilla, Lanzetia, Hierba del Carpintero, Pega-Pega, Siempre Viva, Hierba de la Fortuna, Hierba del Tesoro, Hierba de la Plata, Hierba del Cariño, Guaime-Guaime, Hierba de la Señorita, Hierba del Caballero, and Hierba de la Justicia. After boiling add a bottle of your favorite perfume. Rub the entire body with all the herbs, then rinse with the water and Air dry. Do not use soap or a towel. Bathe 3 times per week.<br>2. Add 3l of water to 15g of the plant material, 10g of Hierba de la Fortuna, El Dolar, Hierba de la Plata, Chupaflor, Hierba del Halago, Tabu, Petalo de Rosas Roja, Blanca, and Roja Amarilla. Also add Agua Florida, white sugar, and Lima juice. Bathe 3 times, that Tuesday, Friday and the following Tuesday. Patient may repeat when needed.<br>3. Standard Seguro mixture, see below.	1., 2., 3. Good luck, Aphrodisiac, Good business, Protection, Good fortune, Good health	EHCHL66, GER130, JULS306
Piper aduncum L.	Yerba del Soldado, Tilonga, Matico, Mogo-Mogo	1. Leaves, fresh or dried<br>2. Leaves, fresh	1. Oral<br>2. Topical	1. Boil 5-10 Leaves per 1l of water for 3-5 min mixed with Salvia Real, Escorsionera, Vira-Vira, Borraja, and Asma Chilca. Drink 1l daily for 15 days.<br>2. Boil 50g per 8l for 10 minutes combined with Eucaliptus, Laurel, Verbena, Altamisa. Bathe twice a week. Alternativel Grind and pulverize 200g of the plant material. Apply the powder on affected areas. Apply once a day, until the wound is healed.	1. Cold, Fungus, Cough, Wounds, Bronchitis, Chills, Tuberculosis, Stopping a hemorrhage<br>2. Wounds reluctant to heal, Immune System, Infection, Inflammation, Bronchitis, Colic (women), Wounds	VFCHL26, RBU/PL277, TRUVan/Erica24, JULS15, GER141, JULS199

Family/Genus/Species	Indigenous name	Plant part used	Admin.	Preparation	Use	Coll. #
Piper cf. *aequale* Vahl.	Modoquero, Mogoquero	Leaves and Stems, fresh or dried	Oral	5g per 1l of water and mix with Flor de Overo, Boldo. Drink 3 times per day for one week.	Liver, Hepatitis, Infection in the body	EHCHL82, RBU/PL272
Piper nigrum L.	Pimienta Negra	Seeds, dried	Oral	Add plant material, Asma Chilca, Borraja, Escorcionera, Muyaca, Vira Vira, Veronica, Cinnamon and a portion of Garlic. Make the mixture concentrated by boiling for 5 minutes. Drink hot. Take 1 cup, 2 times a day, for 2 weeks.	Bronchitis	JULS227
PLANTAGINACEAE						
Plantago linearis H.B.K.	Llantén Serrano, Llantén de la Costa, Llantén	1., 2. Whole plant, fresh<br>3. Root, fresh	1. Topical<br>2., 3. Oral	1. 1 whole plant, boiled with Matico and 01/2l water. When tepid, take out the plant and apply directly to the affected area, twice a day as needed.<br>2.10 grams per 1l water, mixed with Cola de Caballo, Chacur, Unquia, Grama Dulce, Flor Blanca. 4 times per day, 1 month. Harms the vision. Don't take more than one month.<br>3. Boil 2 roots per 1l water for three minutes and combined with Matico, Nogal, Vira Vira, Eucalipto. Drink 4 times a day, as needed.	1. Inflammation of wounds, Wounds (cleansing)<br>2. Liver, Inflammation of the kidneys, Wounds, Bladder<br>3. Cough, Bronchitis	JULS35, JULS86, GER133
Plantago major L.	Llantén	1., 2., 3. Leaves, fresh<br>4. Seeds, fresh or dried	1., 3. Topical<br>2., 4. Oral	1. Boil 6 Leaves per 1l water, 5 min. mix with Matico, Artemisa, Salvia Real, Retania, Piedra Azul. Wash 1 time per day for 8 days.<br>2. Boil 20-30g per 1l, 3-5 min. mix with Ortiga, Berros, Lancetilla, Chanca Piedra, Flor Blanca. Drink 1 time per day for 3-8 days. Taking too much might harm vision.<br>3. Poultice, 5 Leaves with or without water,1-2 times per day for 2-8 days, apply warm.<br>4. 10g or 1Tbsp per 1l of water, one cup in the morning, at noon and one in the evening, before eating.	1. Hemorrhoids, Tumors of the skin (benign), Vaginal cleansing, Wounds<br>2. Blood purification, Inflammation, Liver, Kidneys, Bad breath produced by contamination of an organ<br>3. Arthritis, Pain (physical), Twists, Contusions, Infections<br>4. Bronchitis, Cough, Colic, A blow or bruise that gets infected	VFCHL50, EHCHL11, TRUVan/Erica13
Plantago sericea R. & P.	Rabo de Paloma, Hierba del Susto (de Cerro)	Leaves, fresh	Oral	Boil 2-3g per 1l for 3-5 min. Mix with Toronjil, Mejorana. Drink 3 times per day.	Freight/ Susto	EHCHL98
Plantago sericea R. & P. var. *lanuginosa* Grieseb.	Pajilla Blanca	Whole plant, fresh or dried	Oral	Boil 1l of water with Anga Macha and 10g of Pajilla Blanca for 3 - 4 minutes. Drink warm, 1 cup, 2 to 3 times a day for a month.	Vaginal discharge	JULS207
Plantago sericea R. & P. subsp. *sericans* (Pilger) Rahn	Paja Blanca	Stems, fresh or dried	Oral	Boil 3g per 1l water, no mixing, drink three times per day.	Ovarian pain, Inflammation of the ovaries, Inflammation of the womb	RBU/PL335, EHCHL96

Family/Genus/Species	Indigenous name	Plant part used	Admin.	Preparation	Use	Coll. #
POACEAE						
Arundo donax L.	Cana Hueca, Carrizo	Whole plant, fresh	1., 2. Topical	1. Chop fresh plant from place of growth. Sit by a small creek and have another person chop the tip of the fresh plant and capture the slime that the plant releases. Place it on the affected area. Every morning for a week.<br>2. Let soak overnight 5 Stems springs in 1 glass of water. 1-5 drops in the affected eye 1 time per day for 3 days, or as needed. Dosage depends on the seriousnes of the problem.	1. Haemorrhoids<br>2. Eye scratches, Eye clouds	JULS124, GER38
Cenchrus echinatus L.	Abrojo, Cadillo	Whole plant, fresh	Oral	Boil 100g Abrojo, Amor Seco, Lampazo, Trinozo into 1/2 cup of water for 3 minutes. Drink 1/4 cup 1 time a day for 3 days.	Sharp pain in any part of the body, Inflammation (general), Skin, Intestine, Liver disease, Gallbladder disease, Tumors, Urinary disease	JULS89
Cymbopogon citratus (DC.) Stapf.	Cedron, Hierba Luisa, Maria Luisa	Leaves, Roots and Stems, fresh or dried	Oral	Boil 1l of water, then add 5g of Hierba Luisa. Let sit for 2 to 3 minutes. Add a little Tequila. Stems have more alkaloids and more strength. Patient should drink hot solution. May consume with food best at breakfast.	Cold, Cough, Nerves, Flu, Varicose veins, Stomach Pain, Blood circulation, Cancer	EHCHL16, VFCHL30, JULS181, GER25
Cynodon dactylon (L.) Persoon	Grama Dulce	Stems, dried	Oral	Boil 10g per 1l, mixed with Cola de Caballo, Verbena, Amor Seco, Malva, Flor Blanca, Hierba de Apostema, Zarzaparrilla and Hierba del Toro. Drink 1l daily, 6-12 months.	Cysts of the ovary, Cysts of the uterus, Inflammation of the kidneys, Inflammation (general), Uterus, Fibroids, Uterus prolapse	ISA61, JULS73, ISA106, GER151
Digitaria ciliaris (Retz.) Koehler	Hierba de los Siete Vientos	Leaves and Stems, fresh or dried	Topical	In 1/2 bottle of Cañazo add 200g of Hierba de los Siete Vientos, 1 bottle of Agua Florida, 1 bottle, of Agua Cananga, a few Stems of Hierba del Aire, Ishpingo, Samala, and Hierba del Dominio. Let it sit for 15 days. Spray over the peerson by mouth, twice a week for one month.	Bad Air / Mal Aire	GER69
Gynerium sagittatum (Aublet.) P. Beauvois	Caña Brava					JULS298
Hordeum vulgare L.	Cebada	Seeds, dried	Oral	Boil 2l of water with 250g of toasted Seeds, 50g Linaza and 10g of Cola de Caballo, Amor Seco and Malva. 1 cup 3-4 times a day for 1 month.	Inflammation of the kidneys, Inflammation (general)	JULS128, GER183
Oryza sativa L.	Arroz	Seeds, dried	Oral	Toast 10g of rice until yellow, then place into 1/2l water with 1 piece of cinnamon and 1 tsp sugar. Boil 3-4 minutes. Drink warm, 2 to 3 times a day for 2 days.	Diarrhea, Colic	JULS107, GER231

Family/Genus/Species	Indigenous name	Plant part used	Admin.	Preparation	Use	Coll. #
Saccharum officinarum L.	Azucar de Caña, Caña de Azucar, Caña de Dulce, Caña Dulce	1. Fresh sugar<br>2., 3. Stems, fresh	1. Topical<br>2., 3. Oral	1. Sugar cane candy placed in Potato to ferment. Resulting juice applied to eyes,,.<br>2. Extract juice from the cane, drink cool, 1 glass a day for 2 to 2 and 1/2 months. Contains lots of Calcium.<br>3. 20g of each of the following: Caña Dulce (do not Peel!), Cola de Caballo, Linaza, Chanca Pieda, Boldo, Pata de Perro. Boil in 1/2l of water for 5 minutes. Drink cold. One cup a day for 20 days.	1. Depression, Sorrow, Bronchitis, Aphrodisiac<br>2. Bones (fractured)<br>3. Inflammation of the kidneys, Inflammation of the prostate	VFCHL4, JULS123, GER208
Triticum sativum L.	Trigo	Seeds, dried	Topical	Add 100g of the plant material with 1/2l of water. Boil the mixture for 5 minutes. Apply as a vaginal douche at a temperate temperature. Do not exceed doseage. Apply 3 times a day for 15 days.	Vaginal infection, Vaginal discharge	GER182
Zea mays L.	Espiga de Maiz, Chuno de Maiz, Maiz	1. Flowers, fresh<br>2., 3. Seeds, dried<br>4. Leaves, fresh	1., 2. Oral<br>3. Topical	1. Boil 10g Espiga de Maiz per 1l water for 3 minutes. Drink all, 3 Flowers at four times per day or as needed.<br>2. 1/2l of water, 1/2kg of corn, a bunch of Chancaca and boil for 5 to 10 minutes (until corn is cooked). Hot servings (reheat if not fresh). Once eaten, stay in room, do not come out to rid the chills. 2 times a day for 2 days.<br>3. Grind 1/2kg of corn into 5l of water. Let it sit overnight and bathe at 6 AM. Bathe without soap at 6 AM, once a month.<br>4. Crush 100g of the plant's leaf and Stems and drain the juice out with a piece of cloth. Drink cold during fasting periods. Preferably drink during the hour of breakfast. 1 small glass 1 time a day for 10 days.	1. Kidneys, Inflammation (internal)<br>2. Chills, Pain in the lungs, Kidney Inflammation<br>3. Inflammation (general), Relaxation for angry people<br>4. Bad digestion, Heart burn, Stomach acid	JULS69, JULS139, GER31, GER186
POLEMONIACEAE						
Cantua buxifolia Jus. ex Lam.	Candu	Whole plant, fresh or dried	Topical	1l water with 10 g, add a mixture of 10g of Eucalyptus, Chancas de Muerto, Flor de Chochos. Bathe 3x in one week (Tuesday, Friday, Tueday) in the afternoon. Hit children who do not speak lightly on the mouth to induce speech.	Fright / Susto, Speech impediment	JULS297
Cantua quercifolia Jus.	Dormidera, Hierba Adormecedora, Tutapure Morado (Chico)	Leaves and Stems, fresh	1. Oral<br>2. Topical	1. 3-5g per 1l of water. 1 cup per day in the evening.<br>2. 3l water boiled with the herbs and vaporized. Baño vaporized over half the body. Can combine with Chingue, Huaminga, Chuque. 1 bath per month.	1. Insomnia, Sedative, Good Luck, Nerves<br>2. Daño, Sorcery, Cutaneous allergy	RBU/PL362, EHCHL100, GER144. ISA10

Family/Genus/Species	Indigenous name	Plant part used	Admin.	Preparation	Use	Coll. #
POLYGALACEAE						
Monnina pterocarpa R. & P.	Clarin	Flowers and Leaves, fresh	1. Oral<br>2. Topical	1. Squeeze 10-15 Flowers and leaves to get the juice out. Drink cold, 1/4 small glass 1 a day for 8 days.<br>2. Use same mixture as ear-drops, 5 drops in each eye 2 a day (6 AM and 6 PM) for 3 days.	1. Throat infection<br>2. Ear infection	GER27
Polygala paniculata L.	Canchalagua	Whole plant, fresh or dried	Oral	3-5g per 1l of water, 3 times per day, for one week.	Blood circulation	EHCHL59
POLYGONACEAE						
Muehlenbeckia tamnifolia (H.B.K.) Meisner	Chumbiauri, Chumbiauria	1. Root, fresh<br>2. Leaves, fresh	1. Oral<br>2. Topical	1. 4 kg per tub (16l water), boiled 8 hours down to 2l and ingest orally, taken with Miel de Mexico. Mix with Hierba de Apostema, Hierba China. 1 small cup of tea mixed with 1 cup Miel de Mexico, in the evening, 1 month.<br>2. Crush and mix with Yonqué. Limpia, 2 times a day, no more.	1. Arthritis, Bones, Rheumatism, Sleep aid, Cancer (early stages)<br>2. Fever	RBU/PL309, ISA30
Polygonum hydropiperoides Michaux	Pica Pica	Leaves, fresh				JULS223
Rumex crispus L.	Acelga, Lengua de Vaca, Hojas de Mala Hierba	Whole plant, fresh	1. Oral<br>2. Topical	1. Boil 20g Acelga per 1l water. Drink 3 times per day for 1-11/2 months.<br>2. A whole plant with 1/2l of water. Do not mix with other plants. Elevate legs in "V" position. Pour wash into vagina and allow to sit for 10 minutes. Go to the restroom and contract vaginal muscles to expel wash. Repeat proces one more time immediately.	1. Infection of the uterus, Inflammation of the kidneys<br>2. Inflamation (internal woman parts), Vaginal inflammation	JULS70, EHCHL173
POLYPODIACEAE						
Cheilanthes myriophylla Desv.	Hierba del Dominio	Leaves and Stems, fresh or dried	1. Seguro<br>2. Oral	1. Place in bag 10g of Hierba del Dominio plus 10g Hierba de la Justicia y Hierba del Olvido. Seal. The patient must carry the bag and pray.<br>2. 3-5 per 1l of water. Drink 3 times per day for one week.	1., 2. Dominating a problematic person, Dominating a person who is out of control, Anger, Bad humor	GER94, EHCHL37
Grammitis moniliformis (Lag. ex Sw.) Proctor	Trencilla pequenia	Whole plant, fresh	Seguro	4-5 small Branches.	So that everything continues to go well, So that nothing bad happens to you.	ISA138
Jamesonia goudotii (Hieron) C. Chr.	Hierba del Carpintero	Stems, fresh	Seguro	3 Stems per seguro.	Succes, To open a door that never closes again	ISA146(107a)
Jamesonia rotundifolia Fée	Trencilla Amarilla, Hierba del Oso, Bonito de Oro, Bastón del Inca	1. Whole plant, dried<br>2. Leaves, fresh	1. Seguro<br>2. Topical	1. According to the size of the portion (almost never the root). 3 little Stems minimum for 1 seguro.<br>2. Bath, 200g per 15l water.	1. Good Luck, Fragrance, Strength (large amount), Adornment<br>2. Adornment, Fragrance	RBU/PL343, ISA132, EHCHL26, TRUVan/Erica11, TRUBH22

Family/Genus/Species	Indigenous name	Plant part used	Admin.	Preparation	Use	Coll. #
Polypodium crassifolium L.	Lengua de Ciervo, Lengua de Servio, Calaguala, Lengua de Ciervo	Stems, fresh	Oral	10g per 1l water boiled with 10g total of Pie de Perro, Amor Seco, Cola de Caballo and rind of pineapple. Drink 1l daily for 1 month. Patient should drink lukewarm solution.	Inflammation of the kidneys, Prostate, Bladder, Internal Inflammation, Inflammation, Liver, Kidneys, Ulcers	EHCHL71, TRUBH38, RBU/PL331, RBU/PL332, JULS52, JULS303
PORTULACACEAE						
Portulaca oleracea L. subsp. *tuberculata* Danin & H.G. Baker	Verdolaga	Whole plant, fresh	Oral	In 1l of water, boil for 3 min. Boldo, Flor de Arena, Cola de Caballo. All these together should add 10g. Drink a cup 3 to 4 times a day for a month. Can also be eaten as salad with olive oil, add lemon and salt, once a week.	Bad Liver, Bad kidneys, Hepatitis, Inflammation of the liver, Cleansing of the liver	JULS268
Portulaca villosa H.B.K.	Verdolaga	Root and Stems, fresh	Topical	Crush Stems and Root and drain the extract to use. Shampoo while showering or bathing.	Hair loss, Hygiene	GER171
PROTEACEAE						
Oreocallis grandiflora (Lam.) R.Br.	Rumilanche, Bunbun, Huaminga	Leaves and Stems, fresh or dried	1. Oral<br>2. Topical	1. 5g per 1l water and mixed with Flor Blanca, Flor de Arena. 4 cups per day for 1 month.<br>2. One handful per 3l water. Can combine with Chingue, Polea de Zanahoria Gentil, Conchalalay Blanco, Apostema and Trebol. Bathe once a week.	1. Inflammation of the ovaries, Inflammation of uterus, Inflammation of the kidneys, Inflammation of the liver, Arthritis, Blood<br>2. Daño de Brevaje, Fright / Susto	EHCHL127, JULS31, ISA28, ISA70
PUNICACEAE						
Punica granatum L.	Granada	Peel of the Fruit, fresh	Oral	In 1l of water boil for 3 - 5 min 3/4 of the Fruit Peel and mix with 10g Hinojo and grated Palta rallada Seeds. Drink a glass 3 -4 times a day for 2 weeks, lukewarm.	Diarrhea	JULS159

Family/Genus/Species	Indigenous name	Plant part used	Admin.	Preparation	Use	Coll. #
RANUNCULACEAE						
Laccopetalum giganteum (Wedd.) Ulbrich	Huamanripa, Pacra, Flor de Guarmarya	1., 2. Leaves, fresh or dried<br>3. Whole plant, fresh	1., 2., 4. Oral<br>3. Topical	1. 2 small Leaves per 1/2l water, boil. Drink 1l per day, until 3 months.<br>2. 1 bottle of wine and add 5 to 6 Leaves of Pacra, 1 Ajo, Huevo de Anjelote, 3-4 spoonfuls of honey and pollen (bee), 2 Cholitos (1 Hembra and 1 Macho), 1 Huanarpo (Hembra), 1 piece of Palo Sangre, and a skull of a Pejesapo. Let sit for 1 week. Drink 3 times a day until the bottle is finished.<br>3. Macerate in alcohol or Cañazo with 10g of Ajo de Sacha, 1 plant of Pacra, 3 to 4 of Ajo Macho, 10g of Eucalypto and Molle. Let mixture sit for a week. Rub on affected area as needed.<br>4. Boil 10g of Huamanripa and 10g of Congona with 1/2 cup of water for 10 minutes. Patient should drink cold solution. 1/2 cup 1 time a day in the morning while fasting for 25 days.	1. Cough, Bronchitis, Asthma, Flu, Cold<br>2. Fertilization (Heat Ovaries)<br>3. Rheumatism<br>4. Epilepsy, Heart disease, Palpitation	VFCHL53, RBU/PL321, EHCHL42, JULS284, GER162
Thalictrum decipiens Boivin	Chontilla (Chica)	Whole plant, dried	Topical	Combine with Ajenco, Salva Real, Lailambo, 7 Espiritus, and Agua del Susto, Twice per month or as illnes requires.	Fever, Papera in Children, Mumps	ISA15
ROSACEAE						
Alchemilla nivalis H.B.K.	Hierba del Oso	Leaves and Stems, fresh	Seguro	3 Stems per flask.	To have spiritual strength and power	ISA97
Cydonia oblonga Miller	Membrillo	1. Fruit Peel, fresh<br>2. Fruit pulp, fresh<br>3. Leaves, dresh	Oral	1. Boil 1l water, then add 1 Membrillo Fruit Peel. Add 10g Manzanilla, Toronjil, Hinojo, Madre Selva, and Romero. Patient should drink warm solution. 1 cup 3-4 times a day for 1 month.<br>2. Chop the Fruit pulp and place in 2 cups of water. Boil for 3-5 minutes. Patient should take solution slowly, with a teaspoon. 1 cup every 6 hours. finish the 2 cups. Use for children and pregnant women.<br>3. Boil 1l water, then add 10g Membrillo leaf. Add Manzanilla, Toronjil, Pimpinella, Borraja, Pensamiento, Mejorana, and Romero. Place mixture in hot water and let it sit covered for 2-3 minutes. Drink 1 cup 3-4 times per day for 1 month.	1., 3. Depression, Nerves, Insomnia, Heart problems<br>2. Vomiting, Nausea	JULS194
Fragaria vesca L.	Fresa	Leaves, fresh	Oral	Boil 1l of water, then add 10g of Fresa. Mix with 10g total of Hinojo, Manzanilla and Pimpinella. Let sit for 2-3 minutes. Drink warm, 1 cup 2-3 times a day for 1 month.	Nerves, Insomnia, Heart disease	JULS158
Geum peruvianum Focke	Valeriana	Stems and Fruits, dried	Oral	Boil 1 cup of water and 10g of Valeriana for 3 minutes. drink cold, 1/2 cup 1 time a day, before bed, for 15 days or as needed.	Nerves, Insomnia	GER200

Family/Genus/Species	Indigenous name	Plant part used	Admin.	Preparation	Use	Coll. #
Polylepis racemosa R. & P.	Quinual	Leaves, fresh or dried	1. Oral<br>2. Topical	1. Boil water, then add 5g Quinual per 2 cups hot water. Do not mix with other plants. Administer drink to the mother. No more than 2 cups.<br>2. Boil 1 bundle of Quinual with 4-5l water. Do not mix with other plants. Patient should bathe with tepid water. 2 or 3 times, as needed.	1. Bloating, After birth detoxifyer<br>2. Bad Air / Mal Aire	JULS2
Prunus serotina Ehrh.	Helialiso	Leaves, fresh or dried	Topical	1. Mixed with Altamisa, Ajenco, Ruda, Romero and boil for 20 minutes. Bath 3 times per week.<br>2. Crush 300g, macerate in alcohol, put moist in the gauze and use as poultice for one week.	1., 2. Arthritis, Fractures, Bone pain, Twists	EHCHL94
Prunus serotina Ehrhart subsp. *capuli* (Cav.) McVough	Capuli	Whole plant, fresh	Topical	1l water with 20g Capuli boiled 3 min, or 10g Capuli per 1/2l steeped, mixed with Verbena and Cola de Caballo. Do Not Ingest! Wash with only the water, not with the herbs. 2-3 times per day as needed.	Wounds of the skin	JULS51
Rosa centifolia L.	Rosa de Castilla, Rosa	Flowers, fresh or dried	1. Oral<br>2. Topical	1. Add 10g of plant material with 1/2l water. Add Senn and boil the mixture for 1-2 minutes. Drink warm, 1 cup in one day, only once.<br>2. Should collect the plant late in the afternoon. Soak in 3l of water 100g of roses of each color, 100g of Margaritas. Let sit overnight and add one bottle of Agua Florida (12 oz) and one bottle of perfume (12 oz.) Rub body with Flowers and Leaves. Rinse and air dry. 2 times a week for 7 days.	1. Laxative<br>2. Improvement of health, love, economy	JULS240, GER97
Rubus robustus C. Presl.	Zarzamora, Moyaca, Zarza, Zarza Parrilla, Mora, Cushai	Flowers and Leaves, fresh or dried	1. Topical<br>2., 3. , 4. Oral	1. 5l of water boil for 30min. Bathe 3 times per week.<br>2. 3 Flower buds per cup boiled water, mixed with Llatama. Drink 1l per day, 1 month. Can also be inhaled.<br>3. Chew like gum.<br>4. 1 Tbsp per 3l water. can combine with Moradilla, Sanguinaria, Hierba del Apostema, mix with Chante and Chote. Can use with all most all of the other herbs. Drink 1l daily, 2-3 months.	1. Fright / Susto, Pain of the body<br>2. Diabetes, Cough, Cholesterol (high), Bronchitis<br>3. Thoat (dried), Cannot speak<br>4. Kidneystones, Inflammation of the kidneys, Inflammation of uterus, Arthritis	EHCHL132(a), ISA41, ISA48, JULS47, EHCHL132(b)

Family/Genus/Species	Indigenous name	Plant part used	Admin.	Preparation	Use	Coll. #
Sanguisorba minor Scop.	Pimpinela, Flor de Overa	Whole plant, fresh	1. Oral<br>2., 4. Topical<br>3. Seguro	1. 5g per 1l water, mix with Cadillo, Hierba del Apostema, Esencia de Rosa, Lancetilla, Toronjil, Congona, Clavela, Manzanilla, and Azares. Drink 3 times per day 6-12 months.<br>2., 3. Standard Seguro mixture, see below. Spray the mixture and rub the patient's body with the liquid for good luck. Spray the mixture every Tuesday and Thursday, as needed.<br>4. 50g of all: Hierba del Lucero, Hierba del Este, Ambrocilla, Senorita, Caballero, Pega Pega, Siempre Viva, Carpintero, Waime Waime, Piri Piri (Hembra y Macho), Hierba del Buen Querer, Hierba del Oro, Hierba de la Plata, Hierba del Halago, Sigueme Sigueme, Hierba del Negocio boil into 5-7L water and boil for 20 minutes then add a bit of the following perfumes: Cariño, Dios de la Huaringa, Dios de la Felicidad, San Antonio, Macumba Pusanga, Gran Jefe, Mil Flores, Llama Plata, and Ekeko and let it cool before bathing. Bathe 2 times (Tuesdays and Fridays only) every 3 months.	1. Heart, Nervous system, Nerves, Insomnia, Depression, Heart, Pain of love, Anxiety, Menstrual regulation, Arthritis, Blood, Mal de susto<br>2., 3., 4. Spiritual Flowering, Good luck	EHCHL117, TRUBH35, RBU/PL262, ISA57, JULS25, ISA147(103a), VFCHL20, GER170
RUBIACEAE						
Arcytophyllum nitidum (H.B.K.) Schlecht.	Hierba de la Madrugera	Whole plant, fresh	Seguro	Seguro, 1/5 of the plant per flask.	Do not spend too much money, So that you do not spend on unnecesary items	ISA144(94a)
Cinchona officinalis L.	Cascarilla, Quinuagiro	1. Flowers and Leaves, dried<br>2., 4. Bark, dried<br>3. Root, fresh	1., 2., 3., 4. Oral	1. 1 Tbsp per 1l boiling water, mixed with Flor Blanca, Grama Dulce and Rose essence, 1l daily for 2 months or more.<br>2. Add to a bottle of Abuelo wine or Aguardiente or Cañazo 10g of Cascarilla plus 10g of Palo de Sangre, Palo Huaco, Pacea, Piri Piri and Huanaco. Add some honey. 1 cup 3 times a day as needed Always finish the bottle.<br>3. Boil with 3 tin pans of water, wait until it evaporates, leaving 1 tin. Can be combined with Chumbiauria, Zarzaparrilla, Hierba de la Postema, Poleo de la China. 1 Tbsp per day.<br>4. Boil 50g of Cascarilla in 1 cup of water for 10 minutes. Drink lukewarm 1/4 cup 1 time a day for 15 days.	1. Cough<br>2. Fertility, Sexual potency<br>3. Cancer<br>4. Colds, Rheumatism	RBU/PL314, JULS127, ISA19, GER167
Coffea arabica L.	Café	Seeds, dried	Oral	Boil 1/2l of water. Filter 2 to 3 oz of coffee into the water. Drink whenever needed.	Pain (physical), Alertness	JULS118

Family/Genus/Species	Indigenous name	Plant part used	Admin.	Preparation	Use	Coll. #
Uncaria tomentosa (Willdenow ex Roemer & Schultes) DC.	Uña de Gato, Uncaria Tormentosa, Una de Gato de la Selva	Leaves and Stems, fresh or dried	Oral, Topical	Grind material. Better used dried. Boil 10g per 1l water, 10 min combined with Chanca Piedra, Linaza, Boldo, Flor de Overo, Bolsa de Pastor. Drink 1l daily, three times per day for 15 days at least or as needed. Drink lukewarm. Solution can also be used in a poultice. Wash wound and apply soaked Leaves.	Bronchitis, Kidneys, Asthma, AIDS, Allergies, Rheumatic infections, Cancer, Contraceptive, Ulcers, Prostate, Bladder, Arthritis, Bones, Blood circulation, Hemorrhages (internal), Wounds (internal), Kidney Inflammation	VFCHL11, RBU/PL263, EHCHL103, JULS275, GER230
RUTACEAE						
Citrus aurantium L.	Hojas de Naranja	Small Leaves and Stems, dried	Oral	5g per 1l water, boil and mix with Bolsilla de Menta, Anís. Drink 3 times a day for 1 week.	Nerves, Stomach	EHCHL105
Citrus grandis (L.) Osbeck	Toronja	Fruit, fresh	Oral	Squeeze extract and drink. Take 1 glass in the morning, and 1 glass at night when needed.	Cholesterol, Losing weight, Burning fat	JULS260, GER181
Citrus limetta Riso	Lima	1. Flowers, fresh<br>2., 3. Fruit, fresh	1., 2. Oral<br>3. Topical	1. Boil 1l of water, then add 10g of the Lima Flowers. Combine with Manzanilla, Hinojo, Toronjil, Romero, Borraja, Madre Selva, and Violeta. 1 glass 3 to 4 times a day for 1 month.<br>2. Squeeze juice and remove the Seeds. 1 glass 2 times a day for 2 days.<br>3. Once the limes are cut, the shaman sucks out the lime juice and sprays toward patient. 2 limes per person. The mixture is used during rituals to calm angry patients by sweetening their hearts, taking their bitternes away, and leaving them refreshed.	1. Nerves<br>2. Inflammation of the stomach, Gastritis, Heart disease, Heartburn, Refreshing the stomach<br>3. Spiritual Flowering, Sucking the pain away, Refreshing the patient, Taking bitternes away	JULS182, GER177

Family/Genus/Species	Indigenous name	Plant part used	Admin.	Preparation	Use	Coll. #
Citrus limon (L.) Burm. f.	Limon	1. Flowers, fresh<br>2. Fruit without Seeds, fresh<br>3., 4. Fruit and Fruit Peel, fresh	1., 3. Oral<br>2., 4. Topical	1. Boil 1l of water, then add 5g of the Limon flower. Combine with Manzanilla, Toronjil, Pimpinella, Violeta, and Claveles. Let the mixture sit for 2-3 minutes. Take 1 glass, 3-4 times a day for 1 month, or as needed.<br>2. Prepare a Limon Suazado by removing the Peel and Seeds from 3 limes, adding the limes into a can with a bit of salt and heating the can over a fire for several seconds until limes become sweet. Squeeze the lemon juice onto the affected area (area of stomach, kidney, or ovaries) and cover with a piece of cloth. Apply 3-4 times day, for 2-3 days, as needed.<br>3. Boil 1l of water with 1 lime for 2-3 minutes. Combine with Cola de Caballo, Pie de Perro, Chacur, Amor Seco, and Verbena. Take 1 cup, 3-4 times a day, for 1 month. Solution can also be used to gargle and to wash the hair.<br>4. Place 7 green limes in a pot with 4l of water. Boil the mixture until limes turn yellow, then remove the water and let it cool down until it becomes lukewarm. Add 1 tablespoon of sugar to the temperate water. Apply mixture as a Baño de Florecimiento and a rub. Take 2 limes that were submerged in the water and pray while rubbing limes over patient's body. Repeat until 1 lime is left. With 1 lime, pray the phrase “Que salga lo negativo y entre lo positivo para mi hogar, trabajo, amor, etc.". Finish by rubbing the final lime over the patient's body. Discard of all the limes. After bath you may rinse with cinnamon water. Bathe 3 times: that Tuesday, Friday and the following Tuesday.	1. Nerves, Inflammation of internal ulcers<br>2., 3. Inflammation (general), Inflammation of the kidneys, Inflammation of the ovaries, Inflammation of the stomach, Throat inflammation, Hair loss, Dandruff<br>4. Good luck	JULS183, GER11
Citrus reticulata Blanco	Mandarina	1. Flowers, fresh<br>2. Fruit Peel, fresh	1., 2. Oral	Boil 1l of water for 2-3 min. Add Mandarina and 10g of Mejorana, Toronjil, Pimpinela, Poraja and Manzanilla. 3 times a day for 1 month (1 cup).	Nerves	JULS191
Citrus sinensis (L.) Osbeck	Naranja	1. Flowers, fresh<br>2. Fruit, fresh<br>3. Fruit Peel, fresh	1., 2. Oral	1. 1l water + 10g herbs, 5g of Orange flower in boiling water. Let it sit covered for 3 min. Mix with Melissa, Claveles, Manzanilla, Mejorana, Chancas de Comida and Romero. Drink lukewarm, 3 cups per day for 1 month.<br>2. Squeeze 2 Oranges + 2 Tbl. spoons of milk of Magnesium or cows milk. 1 small glass once a month. Drink while fasting.<br>3. Whole Peel of an orange + 1l water, boil 3-4min. Drink lukewarm, 1 cup 3x per day for one week.	1. Depression, Nerves, Insomnia, Anxiety<br>2. Laxative, especially for children<br>3. Stomach ache	JULS202, GER178

Family/Genus/Species	Indigenous name	Plant part used	Admin.	Preparation	Use	Coll. #
Gardenia augusta (L.) Merr.	Jasmin, Margarita	Leaves, Stems, Flowers, fresh	1. Oral<br>2. Topical	1. 1l of water and 10g of the flower. Place together and boil water over and leave for 2 to 3 minutes. Drink and gargle the solution.2 to 3 times a day for 2 to 3 days or gargle 3 times a day for 2 days.<br>2. In 3l of water soak 100g of Margarita and 100g of Rose petals, let it soak for about a day then add 1 bottle of Agua Florida and 1 bottle of your favorite perfume. Rub body with Flowerss, rinse with perfumed water and air dry. Do not use soap or towel. 2 times a week for 7 days. A commercially available perfume of Jasmin can also be used.	1. Nerves, Inflammation of the throat, Clearing of the voice<br>2. Improvement of health, Love, Economy	JULS175, GER98, GER105
Ruta graveolens L.	Ruda, Ruda (Macho y Hembra), Hierba del Quinde	Whole plant, fresh	1., 4. Oral<br>2. Topical<br>3. Seguro	1. Boil 1l of water with one whole Ruda Hembra plant and Agenciana, Corpus Way, Salvia, Oregano, Molle, Eucalipto, Altamisa, Ajenco and Culantrillo. Allow to boil until there are 3 cups of solution left. For Abortyion use pure Ruda. Patient should drink hot solution. Drink in the morning and at night for 2 days. Be careful when ingesting because herb is extremely hot.<br>2. Bundle the herbs together with Gallinazo, Flor de Retama, Flor de Chochos, Clavel Blanco, Manzanillon, Romero, Agua del Susto, Rosas Blancas, Rosas Amarillas, Rosas Rojas, white sugar, Hierba de la Justicia, Hierba de la Plata, Hierba de la Fortuna, Hierba de Oro, Juice of 3 limes, Perfume "Tabu", Agua Florida and crystallized rock. Rub patient 3 times per month on Tuesday, Friday, and the following Tuesday.<br>3. Whole plant per seguro.<br>4. Crush 20 Leaves and drain the extract. Drink extract at room temperature or mix it with a glass of water. 3-4 drops 1 time only.	1. Abortion, Strong colics, Good luck, Fright / Susto, Heart, Menstrual regulation, Depression, Bad Air / Mal Aire, Rheumatism, Nerves, Vomiting, Nausea<br>2. Fright / Susto, Success, Aphrodisiac, Envy, Improve business, Bad luck, Improve life<br>3. So that all goes well for you, To open a door that never closes again, To obtain success<br>4. For babies who cry to much and/or can not be tranquil.	ISA152, JULS1, TRUVan/Erica20, EHCHL128, VFCHL16, ISA145(108a), GER24
SALICACEAE						
Populus deltoides Bartram	Alamo	Leaves, fresh or dried	Oral	Boil 1l of water, with 10g of material. Add Manzanilla, Toronjil, Pimpinella, Hinojo, Chancas de comida and Cascara de Membrillo. Let mixture sit for 2-3 minutes. Drink warm, 1 cup, 3-4 times a day for 1 month.	Heart, Nerves, Anxiety	JULS93
Salix chilensis Molina	Sauce	Leaves, fresh	1. Topical<br>2. Oral	1. Smash Leaves for juice, apply as enema once. Do not ingest. Use only when the patient is very sick.<br>2. Boil 10g of Sauce and 10 Fruits of Capuli in 1l of water for 30 minutes. Drink warm, 1/2 small cup every time the patient has chills.	1., 2. Hangover fever, Fever, Malaria, Colds	TRUBH25, JULS82, GER39

Family/Genus/Species	Indigenous name	Plant part used	Admin.	Preparation	Use	Coll. #
SAPOTACEAE						
Pouteria lucuma (R. & P.) Kuntze.	Lucuma	Fruit, fresh	Oral	Cut 2 Fruits into pieces and boil in 2 cups of water. Boil for 4 to 5 minutes. Drink warm, 1 cup 2 times a day for 3 days.	Promoting lactation on women after giving birth	JULS186
SAXIFRAGACEAE						
Escallonia pendula (R. & P.) Pers.	Chuque	1. Leaves, dried<br>2. Leaves, fresh	1., 2. Topical	1. 1 bundle with 3l water. Can combine with Huaminga, Chingue, Ishpinguillo, Ajenco, 7 Espiritus. Bath, once a week.<br>2. Poultice, do not mix with other plants, 3 times per week.	1., 2. Arthritis, Bone pain, Sorcery, Rheumatism, Susto of Death	ISA23, ISA63
SCROPHULARIACEAE						
Calceolaria rugulosa Edwin	Potito	Whole plant, fresh	Oral	Add 10g of plant material, Vervena, Cola de Caballo, Pie de Perro, Amor Seco, Llanten and 1l water. Boil the mixture for 3 minutes. Drink warm. Take 1 cup, 3-4 times a day, for 1 month.	Inflammation	JULS232
Caprania peruviana Bentham	Flor Arenilla, Te de Indio	Whole plant, fresh or dried	Oral	Boil 5g per 1l water. Drink 3 times per day.	Urine retention, Inflammation of the urinary tract, Colic, Kidney, Dissolving acids	RBU/PL374, EHCHL170
Escobedia grandiflora (L.f.) Kuntze	Azafran	Flowers, dried	Oral	Boil 1/2l of water for 3 mins with 20g of Azafran. Drink hot, 1 cup in the morning, 1 cup in the night for a week.	Bronchitis, Pneumonia, Chills (general)	JULS110
Galvesia fruticosa J. Gmelin	Curil, Macacha	Flowers, Leaves and Stems, fresh or dried	1 Topical<br>2. Oral	1. 50g per 1/2l of cane alcohol, rub 1 cup daily on affected areas for 1-6 months.<br>2. In 1l of water add 10g of the Flowers and the Stems plus Zarzamora and Matico, Nogal. 3 to 4 times a day for 2 weeks.	1. Arthritis, Rheumatism, Nerve pain<br>2. Cold, Bronchitis, Asthma	VFCHL37, JULS289
SMILACACEAE						
Smilax kunthii Killip & Morton	Palo de la China (Blanco)	Bark, Root and Stems, fresh	Oral	Boil in 6 cans of water and wait until it evaporates, leaving 2 cans. can combine with Quinuagiro, with bee's honey. Mix with Hierba de la Postema. Take 1 Tbsp per day, in the evening.	Cancer (all types)	ISA20
Smilax medica M.Martens & Galeotti	Zarzaparilla, Zarza Parilla	Stems, dried	Oral	In 1 cup of water boil 20g of Zarzaparrilla plus 20g of Congona, Chajur, Matico and Cola de caballo for 5 minutes. Drink in the morning while fasting. 1 cup 3 times a day for 1 month in the morning before breakfast. Repeat if necessary.	Bad Air / Mal Aire, Heart, Inflammation of the kidneys, Inflammation (general)	GER218, JULS273

Family/Genus/Species	Indigenous name	Plant part used	Admin.	Preparation	Use	Coll. #
SOLANACEAE						
Brugmansia arborea (L.) Lagerheim	Mishia Colambo, Misha Morada, Mishia Blanca, Mishia Rastrera, Floripondio, Datura, Misha Toe	1., 5. Leaves, dried<br>2. Whole plant, fresh<br>3. Flowerss and Leaves, fresh<br>4. Leaves, fresh<br>6. Flowerss, fresh	1., 3., 4. Oral<br>2., 5., 6. Topical	1. Boil 3 leaves of Mishia Colambo and 10 leaves of Toro Maique in 1 cup of water until water is reduced to 1/2 cup. Drink cold. Patient should be kept in a dark and quiet room and on a diet of no seafood or spices for 3 days. Afterwards the patient may leave the room, but should rest indoors for 3 more days. 1 small cup 1 a day for 3 days.<br>2. Bath mixture for Protection from Evil, see below. 3 times a day Tuesday - Friday - Tuesday.<br>3. 30g per 8L, boil 1/2 hour, seems like Misha Blanca, Misha Roja, Misha Tigre, and Misha Ganadera, but the hallucinations are weaker. Use with San Pedro, and Hornamo. 1 cup per day. Alternatively chew 1/4 of a leaf. Overdosae is lethal.<br>4. Plant must be gathered at 6 in the morning. Add 2 leaves of the plant material, 1 leaf of MishaAmarilla, 1 leaf of Misha Blanca, 1 leaf of Misha Rosada, 1g of Toromaique and 1g of Toromisha into 1/2 cup of water. Boil the mixture for 5 minutes. Drink the mixture cold. Patient must stay in a dark room for 3 days while maintaining a diet without spices. Three days afterwards, rest. Exceeding the doseage is lethal. Take 1/8 of a small glass.<br>5. Grind leaves and put powder on affected area as needed.<br>6. Place 4 flowers under the pillow in form of a cross.	1. Untangle sorcery, Heal maldad<br>2. Protection from evil<br>3. Hallucinogen, Vision enhancement<br>4. Bad Air / Mal Aire<br>5. Ulcers, Cysts, Wounds on the heel, Ulcers caused by sorcery<br>6. Insomnia	GER64, VFCHL18, GER50, JULS157, GER52
Brugmansia candida Persoon	Mishia Rosada, Miaha, Misha Blanca, Misha Amarilla, Huargua	1. Leaves, fresh<br>2. Leaves, dried<br>3. Whole plant, fresh	1. Oral<br>2., 3. Topical	1. Add 2 leaves of the plant material, 1 leaf of Misha Amarilla, 1 leaf of Misha Blanca, 1 leaf of Rosada, 1g of Toromaique and 1g of Toromisha to 1/2 cup of water. Boil the mixture for 5 minutes. Drink the mixture cold. Patient must stay in a dark room for 3 days while maintaining a diet without spices. Three days afterwards, rest. Exceeding the doseage is lethal. Take 1/8 of a small glass.<br>2. Grind and pulverize the leaves. Place the powder on affected area until healed.<br>3. Bath mixture for Protection from Evil, see below. Bathe once. Mixture can also be taken orally.	1. Bad Air / Mal Aire, Diarrhea, Hallucinogen to see<br>2. Ulcers, Cysts, Wounds on the heel, Ulcers caused by Sorcery<br>3. Protection from evil	GER54, RBU/PL316, RBU/PL327, RBU/PL328, GER51, GER77

Family/Genus/Species	Indigenous name	Plant part used	Admin.	Preparation	Use	Coll. #
Brugmansia sanguinea (R. & P.) D. Don.	Mishia Galga, Misha Rastrera, Misha Guargan, Misha Roja, Guar Guar Rojo, Floripondio, Mishia Roja	1., 2. Leaves and Stems, fresh<br>3. Flowers and Leaves, fresh	1., 3. Oral<br>2., 3. Topical	1. 1/2 cup of water and 50g of Mishia Galga and boil for 3 minutes. Drink cold, on time only.<br>2. Boil 6l of water with 10g each of: Mishia Blanca, Mishia Colambo, Mishia Galga, Mishia Morada, Mishia Roja, Mishia Rosada, Agua de Susto, Hierba del Gallinazo, Flor de Choclo, and Toro Maique for 5 minutes. Recite a prayer. Bathe the patient in the mixture while rubbing him/her with the herbs. Afterwards, rinse the patient in water, and allow him/her to air dry. 3 times a day Tuesday - Friday - Tuesday.<br>3. Boil 6l of water with 10g each of: Mishia Blanca, Mishia Colambo, Mishia Galga, Mishia Morada, Mishia Roja, Mishia Rosada, Agua de Susto, Hierba del Gallinazo, Flor de Choclo, and Toro Maique for 5 minutes. Recite a prayer. Bathe the patient in the mixture while rubbing him/her with the herbs. Afterwards, rinse the patient in water, and allow him/her to air dry. 3 times a day Tuesday - Friday - Tuesday.	1., 2. Bad Air / Mal Aire, Protection from sorcery, Daño, Nervous tension, Susto of spirits/ Susto de espíritus, Negative spirits<br>3. To look into the other world	GER103, EHCHL10, VFCHL23, RBU/PL250, GER53
Capsicum chinense L.	Aji Panca	Fruit, fresh	Incense	1kg of Aji Panca plus 1/2kg sulfur mix and place on top of hot burning charcoal and let the smoke spread. At this time do the spiritual prayers. Smoke should spread around the house, room by room. None should be in the house but the shaman alone doing the spiritual prayers.	Bad Air / Mal Aire	GER203
Capsicum rhomboideum (Dunal) Kunze	Aji Colorado	Whole Fruit, fresh	1. Charm<br>2. Topical	1. Place 3 peppers (green, yellow and red), tie with red ribbon and Ruda (female and male). To be used for protection against envy. Place bunch behind the door of the house. When no longer needed, dispose into the ocean or river. Hang 1 bunch behind door until no longer needed. If in 2 days the peppers in the bunch turn bad, there is a "mal" in the house.<br>2. Wrap one Aji and one whole egg in Algodon Pardo. Rub/ Frotar, Rub the body with water and herbs. Use the prepared bundle to rub the patient from head to toes. Then take Aji and Algodon Pardo far from the house and burn. Crack the egg, and let content fall in a glass with water for further diagnosis.	1., 2. Evil eye/ Mal ojo, Protecting the patient from envy	JULS91

Family/Genus/Species	Indigenous name	Plant part used	Admin.	Preparation	Use	Coll. #
Cestrum auriculatum L'Herit	Hierba Santa, Agrasejo	Leaves, fresh or dried	1., 2., 3. Topical<br>4. Oral<br>5. Incense	1. Boil 10g Hierba Santa per 1l water for 3 minutes. Combine with Cola de Caballo. Wash with herbs and water. Use the plants to clean the wound. 3 times a day until the wound heals.<br>2. Children: Boil 5g per 1/2l water for 2 minutes. Adults: Boil 10g per 1l water for 2 minutes. Add 7 Espiritus and Yonque. Do not mix with other plants. Immerse body in the leaves and bath water or apply as enema. 3-4 times per month. More limpia for higher blood pressure.<br>3. Boil 1 bundle (20g) Hierba Santa per 3l water. Combine with Quinual, Eucalipto, and Romero de Campo. Wash with herbs and bathwater or use for limpia. 3 times per month as needed.<br>4. 5g per 1l with Corpus Way, Carqueja, and Flor de Overo. Drink 1l per day<br>5. 200g of herb placed over hot charcoal. Inhale the smoke produced. 1 time or as needed.	1. Wounds (cleansing)<br>2. Fever<br>3. Relaxant, Fright / Susto, Pain of the body, High blood pressure, Typhoid fever, Preventing spasms after giving birth, Warming women<br>4. Cough, Fright / Susto, Bronchitis, Colic of the stomach, High blood pressure, Typhoid fever, Diabetes, Liver, Cholesterol<br>5. Bad Air / Mal Aire, Colds, Sending away bad shadows	JULS166, RBU/PL281, EHCHL172, ISA122, GER174, EHCHL102
Cestrum nocturnum L.	Flor de Azares	Flowers, fresh	Oral	Boil 1 Tbsp with 1l water and mix with Pimpinela and Cadillo. 1l per day, 1 month.	Heart	ISA142
Cestrum strigilatum R. & P.	Santa María	Flowers, leaves and Stems, fresh or dried	Oral	Boil 1l water, then add 10g of Santa María, Ruda and Orégano and let sit for 2-3 minutes. Patient should drink hot solution. Drink 1 cup, 2 times a day for 2 days.	Control and regulate menstrual cycle	JULS245
Cestrum undulatum R. & P.	Santa María	Flowers, leaves and Stems, fresh or dried	Oral	Boil 1l water, then add 10g of Santa María, Ruda and Orégano and let sit for 2-3 minutes. Patient should drink hot solution. Drink 1 cup, 2 times a day for 2 days.	Control and regulate menstrual cycle	JULS245
Datura ferox L.	Chamico	Leaves, dried	Oral	Ground and boiled. Buy after 6pm so that it is fresh. Dry 2 leaves, then grind into a powder. Add 1 cup of boiling water. Let sit for 3 minutes. Drink hot to lukewarm as needed. Too much could kill someone. Mix with tea, chocolate, or coffee to disguise product.	Bewitching men, Lowering moral	JULS131
Jaltomata sp.	Gato Simuro	Leaves and Stems, fresh or dried	1. Oral<br>2. Topical<br>3. Seguro	1. Boil 10g of Hierba del Tigre, 10g of Hierba del Oso, and 10g of Semora Negra, 3 Leaves of Toro Simuro, and 3 Leaves of Mishia Amarilla in 1/2 cup of water for 5 minutes. Very strong compound. do not exceed the dosage. 1/8 cup 1 time only. Drink cold. Patient should stay inside the house without any light or noise for 3 days. Should also observe a diet (no spices or seafood).<br>2. Bath mixture for Protection from Evil, see below. Only once.<br>3. 2 small branches per seguro.	1., 3. Bad Air / Mal Aire, Fragrance, Good luck for work, Daño (prevention), Undo bad things done to you, Strength, Maldad (cure), Evil eye/Mal ojo<br>2. Protection from evil	GER58

Family/Genus/Species	Indigenous name	Plant part used	Admin.	Preparation	Use	Coll. #
Juanulloa ochracea Cuatrecasas	Cuya Cuya	Seeds, dried	Toical	Grind and grate 1 Seed with Agua Forida, Timolina, and Alcohol. As needed.	Bad Air / Mal Aire, Pain anywhere on the body	EHCHL154
Lycopersicon esculentum Mill.	Tomate	Fruit, fresh	Oral	Squeeze tomato juice out of the Fruit. Drink cool. Drink1 glass a day, for 1 month.	Preventing joint deformation from arthritis	JULS258
Lycopersicon hirsutum Dunal	Ambulluco de Muerto	Whole plant, fresh or dried	Topical	Mix with Flores de Muerto, Zanahoria de Zomo, Poleo Gentil, Bully Vinegar, 7 Espiritus, Agua del Susto. Limpia, 2 times a week.	Susto of Death/ Susto de muerte	ISA31
Lycopersicon peruvianum (L.) Mill.	Tomate de Monte	Whole plant, fresh	Oral	Boil for 5 minutes 100g of the plant material with 1l of water. Drink cold, 1/2 cup, fasting for 5 days.	Inflammation (internal), Urinary infections	GER237
Nicotiana tabacum L.	Tabaco	Leaves, dried	1. Oral<br>2. Topical	1. Mix 1g of Tabaco with Agua Florida, lime juice, Ramillete de Novia, white sugar, Agua Bendita, Cañazo, Agua Florida and Agua Cananga. Let mixture sit for 2 hours. Both shaman and patient inhale mixture through nose during rituals.<br>2. Soak leaf with Yonque and warm up. Mix with a small amount of Trementina. Spread and leave. Place emplasto on affected area and cover with a piece of cloth. 1 time only for 3 days.	1. Improving vision and insight during rituals, Suspend the patient (te voy levantando), Raise the energy of the patient<br>2. Bone fractures	JULS251, GER92
Solanum americanum Mill.	Hierba Mora, Hierba del Susto, Baja del Espanto, Semora	1., 3. Fruits fresh<br>2., 5. Whole plant, fresh<br>4. New shoots, fresh	1., 3., 5. Topical<br>2. Topical<br>4. Oral	1. Crush 20 Fruits to extract juice, 2 drops per nostril.<br>2. Adult: 1l of water per 10g. Children: 1/2l of water per 10g. 3 times per day until all mucus is released.<br>3. Squeeze juice out of the Fruit. Apply Fruit juice on top of the affected area. 1 time a day until healed.<br>4. Boil 1/2 cup of water with 100g of plant material for 5 minutes, mix with Toronjil, Mejorana. Drink 1/4 cup, 1 time only.<br>5. Bath, Boil 5l of water with 100g of: Hierba del Susto, Hierba del Gallinazo, Romero, Paja del Aire, Ashango, Ishpingo, Samalas, Flor de Muerto and Ruda. Boil for 10 minutes. First rub your body with the leaves. Second, rinse with the water. Do not use soap or water to dry. One bath Tuesday or Fridays only.	1. Sinusitis, Flu, Cold, Involuntary urination<br>2. Fever<br>3. Cold sores, Mouth blisters, Herpes<br>4., 5. Fright / Susto	EHCHL125, JULS76, EHCHL87, GER85, GER159
Solanum mammosum L.	Macumamuna, Toro si Muere, Toro Mishia Negro, Toro Mishia Amarillo, Toro Simuro	1. Fruit, fresh<br>2. Leaves, fresh<br>3. Whole plant, fresh	1., 3. Topical<br>2. Oral	1. Use whole Fruit with Agua Florida. Boil for 20 minutes. 1 bath per week, for 1 month. Plant is highly toxic.<br>2. Boil 100g of Toro Mishia Amarillo in 1 cup of water for 10 minutes. Drink 1 cup, cold.<br>3. Bath mixture for Protection from Evil, see below.	1. Good Luck, To gain weight, Bathing livestock<br>2. Bad Air / Mal Aire<br>3. Protection from evil	VFCHL45, GER56, GER55, GER153
Solanum melongena L.	Berenjena	Whole Fruit, fresh	Oral	Blend 2 Berenjena with 1/4 pineapple. Drink 1 glass a day while fasting as needed.	Burn fat, Lose weight	JULS112

Family/Genus/Species	Indigenous name	Plant part used	Admin.	Preparation	Use	Coll. #
Solanum tuberosum L.	Chuno de Papa	Tuber, dried	Oral	1/2kg of Chuño de Papa in 1/2l of water. Add Chancaca, Angamacha, Valeriana Estrella and boil for 10 to 15 minutes or until the starch comes out. Remove it from the flame. Serve hot as a pudding or a candy 3 times a day for 2 days within 10 days of the baby's birth. The preparation makes a kind of candy and should be served hot. Oral it while blowing on it because it should be consumed freshly cooked. Take the last dose in bed so not to go outside in the cold.	After childbirth complications, Bronchitis, Respiratory problems	JULS140, JULS141
Solanum sp.	Tutapure Chico	Leaves and Stems, fresh	Topical	1 handful boiled with 3l water. Mix with Agua del Susto and 7 Espiritus. Can combine with Tutapure grande, Tutapure blanco, Zanahoria. Bathe, twice per month or 1-2 times a week, depending on severity. More often used for children.	Susto of Death, Haunting of a ghost	ISA3
Solanum sp.	Hornamo Simuro	Whole plant, fresh or dried	Topical	Bath mixture for Protection from Evil, see below. Only once.	Protection from evil	GER238
**STERCULIACEAE**						
Theobroma cacao L.	Cacao	Fruit Peel, dried	Oral	1l of water, add 10g of cacao. Boil 2 to 3 minutes. Drink warm, 1 cup 3 times a day for 1 month.	Inflammation of the kidneys	JULS117
**THEACEAE**						
Camellia sinensis (L.) Kuntze	Te	Leaves and Stems, dried	Oral	Roast 20g of rice, grind, and mix with 20g of tea. Boil 1 cup of water for 5 minutes and add the juice of 3 limes after boiling. Drink cold, 1/2 cup 2 times a day until the pain is gone.	Colic, Diarrhea, Stomachache	GER194, JULS256
**THELYPTERIDACEAE**						
Thelypteris cf. *scalaris* (Christ.) Alton	Helecho Macho	Whole plant, fresh or dried	Oral	Plant should be collected at time of need. Boil 10g Helecho Macho with 10g Pata de Gallina, and10g Perejil in 1l of water. Take during the dangerous days (days when the woman is likely to get pregnant).	Contraceptive	JULS291
**THYMELEACEAE**						
Daphnopsis weberbaueri Domke	Los Cholitos, Cholitos	Seeds, dried	Oral	1 wine bottle (Abuelo), with 1 (hembra) Seeds and 1 (macho) Seeds of Cholitos, with Pacea, Huanarpo (Hembra and Macho), honey, pollen, Huevo de Angelote, Chuchuhuasi, Palo Sangre, Palo Huanco, Cascarilla (10g of each) Let it sit for a week in the bottle. Drink 1-2 small cups per day for one week. Drink temperate in the morning while fasting, and in the evening before bed.	Infertility in women	EHCHL153, JULS137, GER216
**TILIACEAE**						
Mutingia calabura L.	Cerezo Cimarron	Fruit, fresh	Oral	Liquify/blend 200g of the Fruit with 1/2 cup of water. Drink cold, 1 glass 1 time a day for 6 days.	Gastritis, General internal infections	GER168

Family/Genus/Species	Indigenous name	Plant part used	Admin.	Preparation	Use	Coll. #
Tilia platyphyllos Scop.	Tilo	Flowers and Leaves, fresh	Oral	Boil 1l of water, then add 10g of Sauco. Add Manzanilla, Hinojo, Coleo, Ajenjo, Toronjil, Pimpinela and Claveles. Cover and let it sit for 2-3 minutes. Patient should drink warm solution, 3-4 cups per day for 1 month.	Nerves, Cough, Cold, Fever, Insomnia	JULS257
TROPAEOLACEAE						
Tropaeolum minus L.	Mastuerzo	1. Flowers, fresh<br>2. Whole plant, fresh	1. Topical<br>2. Oral	1. Rub Flowers on affected area (usually the face). Make sure Flowers are not wet. 3 times per day, as needed.<br>2. Boil 10g of Mastuerzo with 1l of water. Combine with Amor Seco, Chacur, Cola de Caballo, Verbena, and Espiga de Maiz. Drink 3 cups a day for 1 month.	1. Sun spots<br>2. Inflammation of the stomach	JULS81
TYPHACEAE						
Typha angustifolia L.	Chante	Stems, dried	Oral	1 Tbsp per 1l water. Can combine with Aguilla, Achote. Drink 1l daily.	Prostate	ISA45
ULMACEAE						
Celtis loxense C.C. Berg	Palo Huaco, Palo Blanco	Bark, Stems and Leaves, dried	1. Oral<br>2. Topical	1. Add plant material, Palo Sangre, Chuchuasi, Huanaco, Huevo Angelote, Pacra, Pollen, Miel de Palo, Honey, Chuchuwasi, Cascarilla and Huanarpo Macho into a mixture with 1 bottle of Abuelo wine or Tequila. Let mixture sit for 1 week. Drink cold, 1 small wine glass 3 times a day until bottle is finished. Patient can repeat the treatment.<br>2. Bath, Boiled for 5 minutes. 1 handful in 3l water. Can combine with Conchalay Blanco, Tutapure de Estrella, Timolina and Agua del Susto. 3 baths per week.	1. Fertility, Sexual potency, Arthritis, Bronchitis, Muscle pain, Blood circulation, Hemorrhages (healing)<br>2. Susto of animals, Susto of water, Susto in adults	JULS208, EHCHL65, GER87, ISA7
URTICACEAE						
Pilea microphylla (L.) Lieberman	Contra Hierba	Whole plant, fresh	Oral	3-5 minutes mixed with Cola de Caballo, Hoga de Achote, Chanca Piedra. Drink 1l per day for 15 days.	Bladderstones, Inflammation of the kidneys, Prostate, Cysts	RBU/PL282, EHCHL33
Urtica magellanica A. Jussieu ex Poiret	Ortiga, Ortiga (Chica), Ortiga de Obeja, Ortiga Negra, Hortiga	Whole plant, fresh or dried	1. Oral<br>2. Topical	1.Boil 10g Ortiga Negra per 1l water. Combine with Huamanripa, Veronica, Corpus Way, Karqueja, Nogal, Ocalito, Molle, and Ruda and Matico. 4 cups per day for 15 days.<br>2. Use same mixture for bath and rub leaves on parts afflicted with rheumatism. 3 times per week.	1., 2. Blood purification, Happiness, Fever, Rheumatism, Arthritis, Blood circulation, Hemorrhages, Hair loss, Asthma, Hemorrhoids, Inflammation (general)	RBU/PL251, ISA119, JULS11, EHCHL50, VFCHL9, GER161

Family/Genus/Species	Indigenous name	Plant part used	Admin.	Preparation	Use	Coll. #
Urtica urens L.	Ortiga	Stems and Leaves, fresh	1. Topical	1. 7 Stems with their Leaves boiled with 3-5l water, combined with Agua del Susto, Ajenco, and Llatama for 20 minutes. Bath, 2-3 times per week, at 7,9, and 11PM.	1. Bad Air / Mal Aire, Prostate, Fright / Susto, Vaginal cleansing, Business, Casting away bad luck, Freight in children / Susto en niños	RBU/PL299,
VALERIANACEAE						
Phyllactis rigida (R. & P.) Persoon	Hornamo Estrella, Siete Sabios, Valeriana Estrella, Valeriana, Hierba de la Estrella	Stems, fresh	1. Seguro<br>2., 4., 5., 6. Topical<br>3. Oral	1. Mix with other herbs of strength, herbs of luck for seguro.<br>2. 20g per 5l, boil 20 min; mix with other herbs of strength and herbs of luck.. Bathe 3 times a week.<br>3. Boil 1l water, then add 10g Valeriana Estrella. Drink 4 times per day, as needed. Children can't take it very often (start taking at 6 years).<br>4. Combine with Timolina, Bully vinegar, Agua Florida, and Arnica. Put on the back of the head, or afflicted area. Put it on daily through the night.<br>5. Alternatively in a bottle place 1g of each of the following: Hierba del Lucero, Hierba Este, Ambrocilla, Senorita, Caballero, Pega Pega, Siempre Viva, Carpintero, Waime Waime, Piri Piri (Hembra y Macho), Hierba del Buen Querer, Hierba del Oro, Hierba de la Plata, Hierba del Halago, Sigueme Sigueme, Hierba del Negocio. 1 bottle of perfume "Cariño" and a bit of the following perfumes: Dios de la Huaringa, Dios de la Felicidad, San Antonio, Macumba Pusanga, Gran Jefe, Mil Flores, Llama Plata, and Ekeko. Some spiritual prayer invoking the name of the patient, owner of the seguro. Fogear. Spray and rub the patient with the mixture for good luck. Tuesdays and Fridays. 6. 50g of all: Hierba del Lucero, Hierba del Este, Ambrocilla, Senorita, Caballero, Pega Pega, Siempre Viva, Carpintero, Waime Waime, Piri Piri (Hembra y Macho), Hierba del Buen Querer, Hierba del Oro, Hierba de la Plata, Hierba del Halago, Sigueme Sigueme, Hierba del Negocio boil into 5-7L water and boil for 20 minutes then add a bit of the following perfumes: Cariño, Dios de la Huaringa, Dios de la Felicidad, San Antonio, Macumba Pusanga, Gran Jefe, Mil Flores, Llama Plata, and Ekeko and let it cool before bathing. Bathe 2 times (Tuesdays and Fridays only) every 3 months.	1. Fragrance, Good luck, To always shine<br>2., 5., 6. Spiritual Flowering, Good luck, Bad Air / Mal Aire, Success<br>3. Insomnia, Relaxant, Sleep aid, Nerves, Headache, Menopause<br>4. Contusions, Mental disorders, Schizophrenia, Cerebral pain	EHCHL163, TRUBH30, JULS57, EHCHL44, JULS46, ISA137, RBU/PL365 , RBU/PL355, GER187

Family/Genus/Species	Indigenous name	Plant part used	Admin.	Preparation	Use	Coll. #
Belonanthus aff. *hispidus* (Wedd.) Graebn.	Boton de Oro	Whole plant, fresh or dried	1. Topical<br>2. Seguro	1. 3l water with 10g of Boton de Oro, and 10g each of Hierba de la Justicia, Hierba del Halago, Hierba de la Plata, Hierba de la Fortuna, Dolar, Sigueme Sigueme, boil 3min, add Agua Florida, Agua Tabu, white sugar, and Lima juice. Bathe 3 x a week, on Tuesday, Friday and Tuesday.<br>2. Prepared with perfumes and the typical Seguro herbs. One Seguro, refill perfumes as needed, keeps its power as long as filled.	1., 2. Good luck	JULS299
Valeriana bonplandiana Wedd.	Fortuna	Whole plant, fresh or dried	Topical	10g per 1l boiling water, 2 baths per month, during the evening.	Fragrance, Good Luck	RBU/PL350
Valeriana plantaginea Kunth	Hornamo Morado, Hornamo Caballo, Horno Morado	Leaves and Stems, fresh	1. Oral<br>2. Topical	1. Boil 10g Hornamo Morado with 1/2 cup water for 2 minutes. Patient should drink cold solution. 1/2 cup 1 time only.<br>2. Boil 3l water for 10 minutes with 100g Hornamo Morado, with 10g each of: Mishia Blanca, Mishia Colambo, Mishia Galga, Mishia Morada, Mishia Roja, Mishia Rosada and Toro Maique. Recite a prayer. Patient should rub with herbs. When the bath is finished, the patient should not rinse or use a towel, but air dry only.	1. Bad Air / Mal Aire, Purgative, Laxative<br>2. Protection	GER193, EHCHL91, EHCHL120
VERBENACEAE						
Aloysia triphylla (L. Her.) Britt.	Cedron, Pepas de Cedron, Sidrón,	1. Whole plant, fresh<br>2., 3., 4. Seeds, fresh or dried	1., 3., 4. Oral<br>2. Topical	1. Boil 1l water, then add Cedron, and mix with Chancas de Comida, Toronjil, Hinojo, Madre Selva, Claveles, and Pensamiento. Use a total of 10g for all the material. Let the mixture sit for 2-3 minutes. Patient may drink solution at all temperatures, but it is recommended to drink while lukewarm. Take 1 cup, 3-4 times a day for one month.<br>2. Grind 4-5 Seeds and boil in 5l water. Bathe.<br>3. Boil 1 Seeds per 1l, drink 4 cups per day, 7 days.<br>4. Mix the grind of 10g of Seeds, 1/4 of a small cup of pisco, 1g of Alucema, 1g Oregano and 1g of Pimienta. Warm up. Drink 1 tablespoon a day for 6 days.	1. Depression, Nerves, Insomnia, Stomach<br>2., 3. Bad Air / Mal Aire<br>4. Sharp pain in the body	JULS130, RBU/PL384, EHCHL161, RBU/PL305, GER90
Clerodendron sp.	Brochamelia	Flowers, fresh or dried	Oral	1l of water and add 10g of the herb. Boil for 3 to 5 minutes. Can be mixed with 10g of Huamanripa and Veronica. Drink 1 cup 3 times a day for 2 weeks. Toz Ferina indicates a condition, where a baby can't breathe and turns blue and makes a "rooster like" noise.	Bronchitis, Asthma, Whooping cough	JULS115
Lantana scabiosaefolia H.B.K.	Mastrando, Mastrante	Leaves and Stems, fresh or dried	Oral	20-100g per 1l water, boil 3 min. mix with Canchalagua, Culantrillo, Purenrosa, Panisara, and Salvia Real. 1l per day, 3 days. Patient should drink lukewarm solution. This treatment is only for women.	Cold, Cold of the ovaries, Menstruation, Women after childbirth to avoid colds	VFCHL51, GER6

Family/Genus/Species	Indigenous name	Plant part used	Admin.	Preparation	Use	Coll. #
Lippia integrifolia (Grieseb.) Hieron	Poleo del Inca	Leaves and Stems, fresh	Oral	5g per 1l water, 1l daily, 1 month.	Cold, Colic, Inflammation of the kidneys, Bronchitis, Rheumatism, Gases	EHCHL76
Verbena littoralis H.B.K.	Verbena, Berbena	Whole plant, fresh or dried	1., 2. Topical<br>3. Oral	1. Adults: 10g of Verbena per 1l of water. Children: 10g of Verbena per 1/2l of water. Apply enema when water is lukewarm. Once only. Alternatively Boil 10g per 2l of water for 30 minutes, combined with Matico, Malva, Llantén, and Para Para. 3 times per day for 8 days.<br>2. Boiled for 20 min with 5-10g of herb per liter of water, mixed with Matico, Malva, and Manzanilla. Bathe 3 times per week.<br>3. Boil 30g per 1l for 3 min., mix with Cerraja, Moradilla, and Verdolaga. 2 glasses per day for 4 days. Take one in the morning and one at night.	1. Fever, Fungus<br>2. Hyperactivity, ADHD, Tranquility<br>3. Inflammation, Wounds (cleansing), Blood purification, Cholera, Lower strong character, Colic, Colds	RBU/PL369, JULS77, EHCHL69, VFCHL28, GER138
	Llatama Blanca	Leaves and Stems, dried	Oral	Boil 1 cup of water in 100g of plant material for 10 minutes. Drink cold, 1/4 cup 2 times a week.	Quema rastro, Feet blisters	GER82
VIOLACEAE						
Viola tricolor L.	Pensamiento Amarillo, Hierba del Pensamiento, Hierba del Tacón	Whole plant, fresh or dried	Oral	Boil 1l water, then add 10g Pensamiento Amarillo, used with plants for the heart, including Toronjil. Drink 3 cups per day as needed.	Heart, Pain of love, Nerves, Insomnia, Forgetting pain, Fright / Susto, Bad Air / Mal Aire, Nerves, Epilepsy	JULS36, VFCHL19
VITACEAE						
Vitis vinifera L.	Uva	Fruits, dried	Oral	Add 1/2l of fresh milk with 10g of dried grape (raisin). Boil the mixture for 3-4 minutes. Drink hot. Take 1 glass, 3 times a day for 2 weeks.	Bronchitis, Laxative	JULS266
XYRIDACEAE						
Xyris subulata R. & P.	Hierba del Caballero, Chupa Flor	Stems, fresh	1. Seguro<br>2. Topical	1. Standard Seguro mixture, see below.<br>2. 2. Standard mixture for Spiritual Flowering, see below.	1., 2. Asking that a woman become your love, Fragrance, Good luck, Good business, Protection, Good fortune, Good health	ISA103, RBU/PL349, JULS300, GER132, JULS306
ZINGIBERACEAE						
Zingiber officinale Roscoe	Kion, Quion, Gengibre, Gengible	Root, fresh	Oral	Cut Kion into small pieces. Add 10g of this, along with Matico, Nogal and Veronica. Boil in 1/2l of water. Take 1 cup, 3 times a day for 1 week.	Cold, Cough, Bronchitis	JULS237, GER206
	Chima Pampana	Tuber, fresh	Topical	Use the red or purple tuber. Grate to create a light perfume, 1 half a tuber per ointment container. Mix with Cariño perfume. Place perfume on the body as needed.	To get a female sexually interested, To get a male sexually interested, To trap a man	JULS55

Family/Genus/Species	Indigenous name	Plant part used	Admin.	Preparation	Use	Coll. #
ZYGOPHYLLACEAE						
Tribulus terrestris L.	Abrojo, Cadillo	Whole plant, fresh	Oral	Boil 100g Abrojo, Amor Seco, Lampazo, Trinozo into 1/2 cup of water for 3 minutes. Drink 1/4 cup 1 time a day for 3 days.	Sharp pain in any part of the body, Inflammation (general), Skin, Intestine, Liver disease, Gallbladder disease, Tumors, Urinary disease	GER137
LICHENES						
Siphula sp.	Palalio, Papelillo, Papelilla	Leaves, fresh	Oral	5g per 1l water.	Liver	EHCHL115, JULS216
ALGAE						
Giartina chamissoi	Cochayuyo, Mococho	Whole plant, fresh	Oral	Steam 20g of plant material in hot water. Blend the steamed material with 3-4 oranges. Take 1 glass 2 times a day, for 1 month.	Lose weight, Cholesterol	JULS146
Giartina glomerata	Cochayuyo, Mococho	Whole plant, fresh	Oral	Steam 20g of plant material in hot water. Blend the steamed material with 3-4 oranges. Take 1 glass 2 times a day, for 1 month.	Lose weight, Cholesterol	JULS146
Giartina paitensis	Cochayuyo, Mococho	Whole plant, fresh	Oral	Steam 20g of plant material in hot water. Blend the steamed material with 3-4 oranges. Take 1 glass 2 times a day, for 1 month.	Lose weight, Cholesterol	JULS146
INDET.						
	Samala, Asmala, Amara	Seeds, dried or fresh	1. Topical<br>2. Oral<br>3. Blown on patient	1. Bath, 20 Seeds per 5l water, ground and boiled. 3 times per week, or 2 a month, Tuesday and Friday.<br>2. Beverage: 7-15 Seeds per 1l water, crushed and macerated for 8 days. 3-4 small cups per day, 7 days. Seeds can be also macerated in alcohol for 5 days, then 1 Tbsp, 3 times per day.	1., 2., 3. Fright / Susto, Bad Air / Mal Aire, Nervous system, Enchantment, Sorcery, Getting rid of daño, Epilepsia	RBU/PL378, EHCHL158
	Anti Ajo	Stems, fresh	Charm	2l of water with 40cm of the Anti Ajo Stems. Boil 3 hours. let it sit 5 minutes. Use Ruda (Hembra and Macho) and dip into tizana. Use Ruda to distribute the solution to every corner of the house, always going from right to left. Splash the remainder of the solution in front of the house.	Sorcery within a house	JULS103
	Hierba del Hongo	Whole plant, fresh or dried	Topical	Boiled, 5 mins, with 1l water or 5g with 3l, 2 Tbsp Bully Vinegar, more lemon juice, mixed with Manzanilla Blanca, Laurel, Llantén and 1 Tsp, washing for for feet, hands, and other parts, 1 wash per day, until symptoms are alleviated, afternoons.	Concussions, Anemia, Build up of liquid in tissues or wounds, Kidneys	ISA94

Family/Genus/Species	Indigenous name	Plant part used	Admin.	Preparation	Use	Coll. #
	Huarate	Stems, dried	Oral	Boil 1l of water, then add 10g total of Manzanilla, Toronjil, Pimpinela, Hinojo, and the Huarate Stems. Let mixture sit for 2 minutes. Patient should drink lukewarm solution. 1 cup 3 to 4 times a day for one month. Also used by bad shamans for daño and burn anything that can trail it back to them.	Diabetes, Nerves	JULS173
Trichilia ?	Pucho	Leaves and Seeds, dried	Oral	Gather Jan-Feb, when there is 'pollen'	Nervous system	RBU/PL380
NON PLANT MATERIAL						
	Polen de Zapote, Polen de Espina Negra, Polen de Arboles, Polen de Ciachon (Insect feces)	Insect feces	Oral	Insect larvae bore into the root of the tree. Use the feces of the larvae ('pollen'). 4g per 1l water. Is very strong, so use a small amount. 1l daily, 1 month	Bronchitis, Asthma, Tuberculosis	ISA124
Standard Seguro mixture:				1 small Stems per seguro. Mix all of the of the following in a bottle of perfume: 3 Stems and Leaves of Hierba de la Señorita Hierba del Buen Querer, Palmerilla, Destrencilla, Lanzetia, Hierba del Carpintero, Pega-Pega, a bit of the following:, Palmerilla, Destrencilla, Lanzetia, Siempre Viva, Hierba de la Fortuna, Hierba del Tesoro, Hierba de la Plata, Hierba del Cariño, Guaime-Guaime, Piri- Piri, Hierba del Caballero, Hierba de la Justicia (amount depends on the size of the bottle). Add perfume "Cariño" and a bit of the following perfumes: Dios de la Huaringa, Dios de la Felicidad, San Antonio, Macumba Pusanga, Gran Jefe, Mil Flores, Llama Plata, and Ekeko. The Shaman will then bless it and Fogear. Keep the seguro bottle at home, or if is small enough carry it with you at all times.		
Standard mixture for Spiritual Flowering:				3l water, boil for 30 minutes with 10g Chupaflor, and 10g each of Hierba del Buen Querer, Palmerilla, Destrencilla, Lanzetia, Hierba del Carpintero, Pega Pega, Siempre Viva, Hierba de la Fortuna, Hierba del Tesoro, Hierba de la Plata, Hierba del Cariño, Guaime Guaime, Piri Piri, Hierba de la Señorita, Hierba de la Justicia, Hierba de la Fortuna, El Dolar, Hierba de la Plata, Hierba del Halago, Tabu, Petalo de Rosas Roja, Blanca, and Roja Amarilla. Also add Agua Florida, white sugar, and Lima juice. After boiling add a bottle of your favorite perfume. Hierba de la Plata, Hierba de la Fortuna, white/yellow and red Roses, Agua Tabu, Agua Florida, white sugar, limas juice. Bathe 3 times, that Tuesday, Friday and the following Tuesday. Patient may repeat when needed. Rub the entire body with all the herbs, then rinse with the water and air dry. Do not use soap nor a towel.		

Alternative mixture for Spiritual Flowering:				In 3l of water boil for 30 minutes 3 Stems and leaf of Hierba de la Señorita plus a bit of the following: Hierba del Buen Querer, Palmerilla, Destrencilla, Lanzetia, Hierba del Carpintero, Pega-Pega, Siempre Viva, Hierba de la Fortuna, Hierba del Tesoro, Hierba de la Plata, Hierba del Cariño, Guaime-Guaime, Piri-Piri, Hierba del Caballero, Hierba de la Justicia after boiling add a bottle of your favorite perfume, Rub the entire body with all the herbs, then rinse with the water and air dry. Do not use soap nor a towel, only once.		
Bath mixture for Protection from Evil				Boil 6l of water with 10g each of: Mishia Blanca, Mishia Colambo, Mishia Galga, Mishia Morada, Mishia Roja, Mishia Rosada and Toro Maique for 5 minutes. Recite a prayer. Bathe the patient in the mixture while rubbing him/her with the herbs. Afterwards, rinse the patient in water, and allow him/her to air dry.		
